# Supplementary material for: Structures of Respiratory Supercomplex I+III2 Reveal Functional and Conformational Crosstalk
Source: Mol Cell. 2019 Sep 19;75(6):1131–1146.e6. doi: 10.1016/j.molcel.2019.07.022 (PMC6926478; doi:10.1016/j.molcel.2019.07.022)
Supplement: Document S1. Figures S1–S7 and Tables S1–S5 [file mmc1.pdf]

Molecular Cell, Volume *75*

Supplemental Information

Structures of Respiratory Supercomplex I+III$_2$ Reveal Functional and Conformational Crosstalk

James A. Letts, Karol Fiedorczuk, Gianluca Degliesposti, Mark Skehel, and Leonid A. Sazanov

SUPPLEMENTAL INFORMATION

Structures of Respiratory Supercomplex I+III_2 Reveal Functional and Conformational Crosstalk

James A. Letts, Karol Fiedorczuk, Gianluca Degliesposti, Mark Skehel and Leonid A. Sazanov

LIST OF SUPPLEMENTAL INFORMATION

Supplemental Figures

Figure S1. Initial isolation and separation of SC I+III_2, Related to Figure 1
Figure S2. Effect of PO_4 buffer on isolated SC I+III_2 oxidoreductase activity, Related the Figure 2
Figure S3. Inhibition of SC I+III_2 activities by known CI and $CIII_2$ inhibitors, Related to Figure 3
Figure S4. Initial cryo-EM processing steps, Related to Figure 4
Figure S5. Focused refinements, Related to STAR Methods
Figure S6. Focus-Revert-Classify strategy to separate different classes of CI, Related to Figure 5
Figure S7. Examples of match between local resolution and model ADPs for isolated CI and SC I+III_2, Related to Figure 7 and STAR Methods

Supplemental Tables

Table S1. Mass Spectrometry identification for subunits of ovine SC I+III_2, CI and $CIII_2$, Related to Figure 1
Table S2. Lipid content of isolated SC I+III_2 particles compared to mitochondrial membranes, Related to STAR methods
Table S3. K_{cat} for CI and $CIII_2$ within the SC and Rate Coupling, Related to Figures 2 and 3
Table S4. Cryo-EM data collection, Related to Figure 4
Table S5. SC I+III_2 closed model overview, Related to STAR Methods

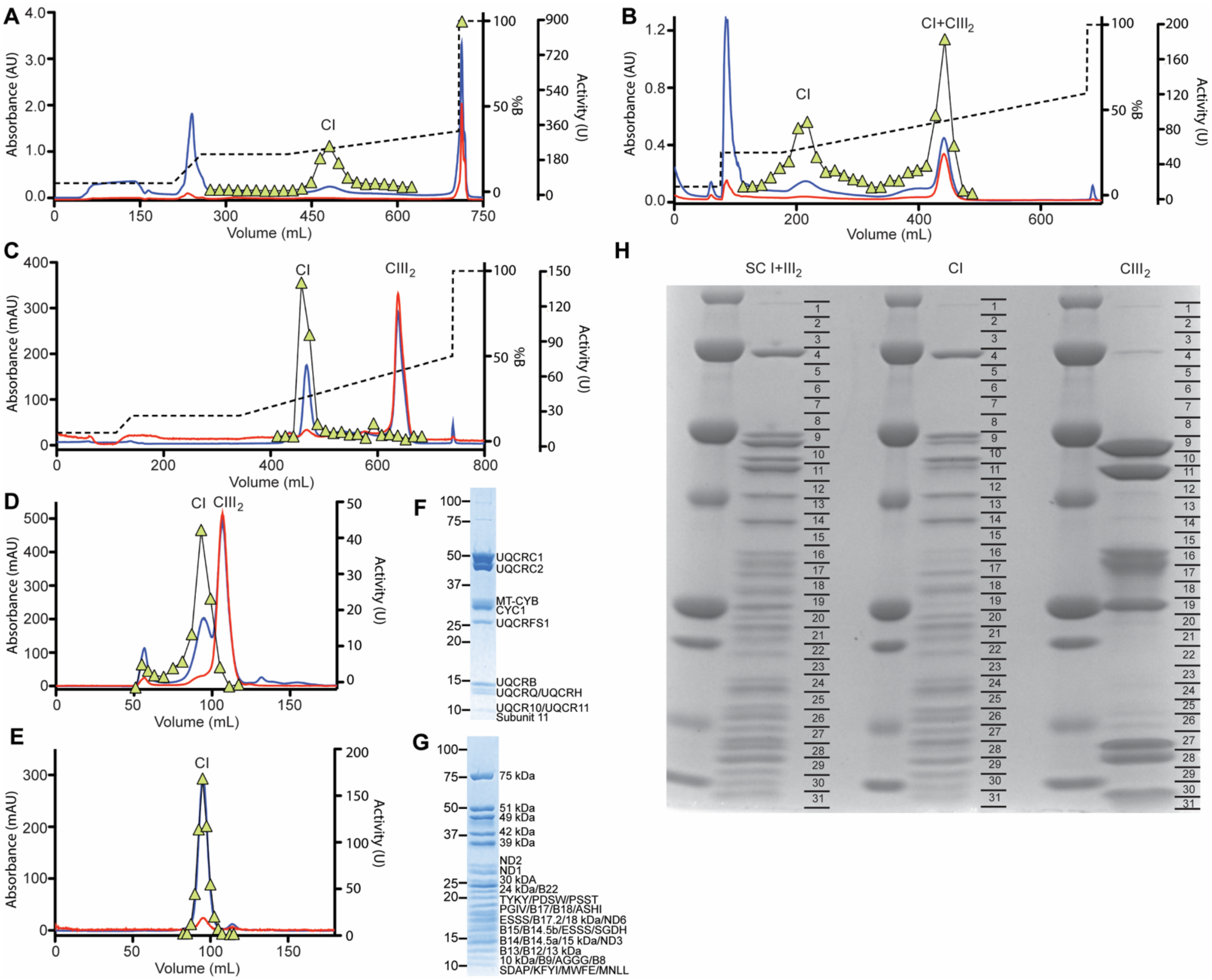

Figure S1. Initial isolation and separation of SC I+III$_2$, Related to Figure 1

(A) Q-Sepharose anion-exchange column chromatogram of LNMG-extracted washed mitochondrial membranes. In all panels the chromatograms show: A_{280}, blue line; A_{420}, red line; percent buffer B gradient (containing 1M NaCl), dashed black line; and CI NADH:FeCy activity, lime green triangles. Unlike purifications in DDM, high CI NADH:FeCy activity was seen in the high-salt wash. (B) Altering the gradient profile resulted in a second peak of NADH:FeCy activity associated with high A_{420} signal, indicating the presence of heme groups. (C) Re-running the second peak from (B) over anion-exchange resulted in the separation of peaks containing NADH:FeCy activity and most of A_{420} signal alone. (D) Running the second peak from (B) over size exclusion chromatography column also resulted in the separation of peaks containing NADH:FeCy activity and most of A_{420} signal alone. (E) Running of peak containing NADH:FeCy activity from (C) resulted in highly enriched sample of CI. (F) SDS-PAGE gel of A_{420} containing peak from (C) with CIII$_2$ subunits identified by mass spectrometry (MS). (G) SDS-PAGE gel of NADH:FeCy activity containing peak from (E) with CI subunits identified by MS. (H) SDS-PAGE showing SC I+III$_2$ sample in amphipol A8-35 (**Figure 1**C), isolated CI (**Figure S1**E) and isolated CIII$_2$ (**Figure S1**C). Each lane was cut into 31 approximately even bands (indicated on right-hand side of each lane), which were each subjected to MS analysis for protein identification. The results of MS analysis are shown in **Table S1** and the corresponding band for each subunit is indicated. The total amount of protein loaded in the gel was not controlled

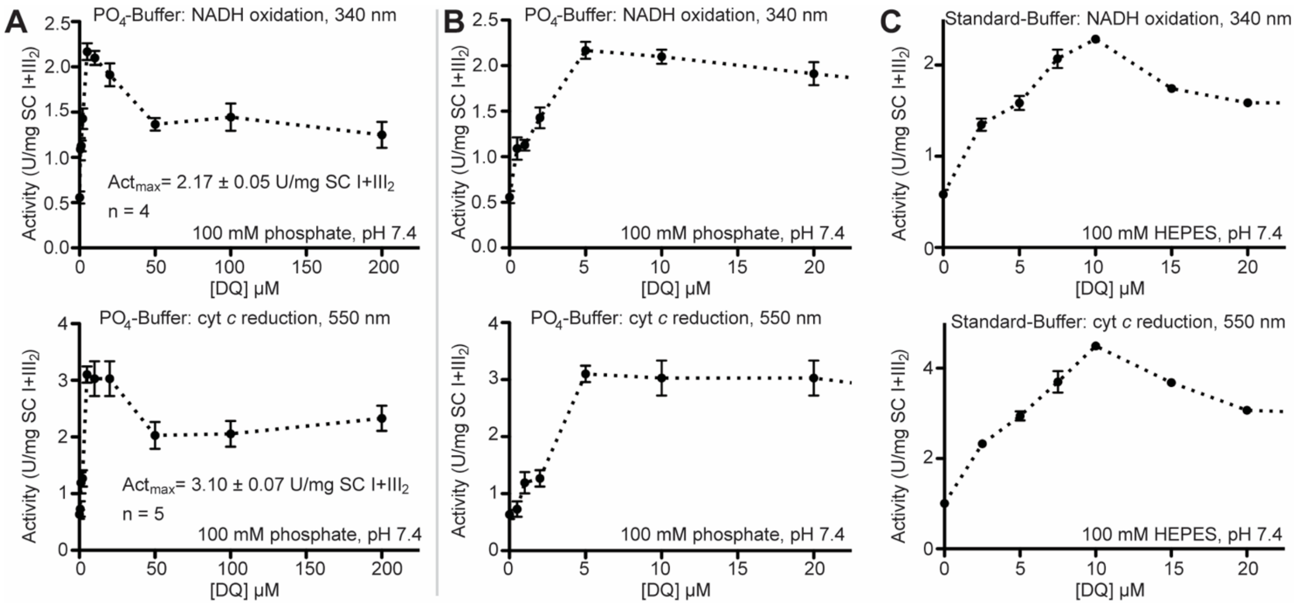

Figure S2. Effect of PO_4 buffer on isolated SC I+III_2 oxidoreductase activity, Related the Figure 2

(A) [DQ]-activity curves monitoring NADH oxidation (top) and cyt *c* reduction (bottom) in 100 mM PO_4 buffer with 100 µM NADH and 100 µM cyt *c*. (B) Expansion of x-axis (0-20 µM DQ) for [DQ]-activity curves from **Figure S2**A, NADH oxidation (top) and cyt *c* reduction (bottom). (C) Expansion of x-axis (0-20 µM DQ) for [DQ]-activity curves from **Figure 2**D, NADH oxidation (top) and cyt *c* reduction (bottom). Activity is reported as Units (1 U = 1 µmol substrate/min per mg of SC I+III_2). Data plotted as mean ± SEM. Regardless of the broader high-activity range in the phosphate buffer, the overall cyt *c* reduction activity was lower compared to HEPES. This is likely due to the known strong dependence of the interaction between $CIII_2$ and cyt *c* on ionic strength. Therefore, HEPES buffer was used throughout for further activity measurements.

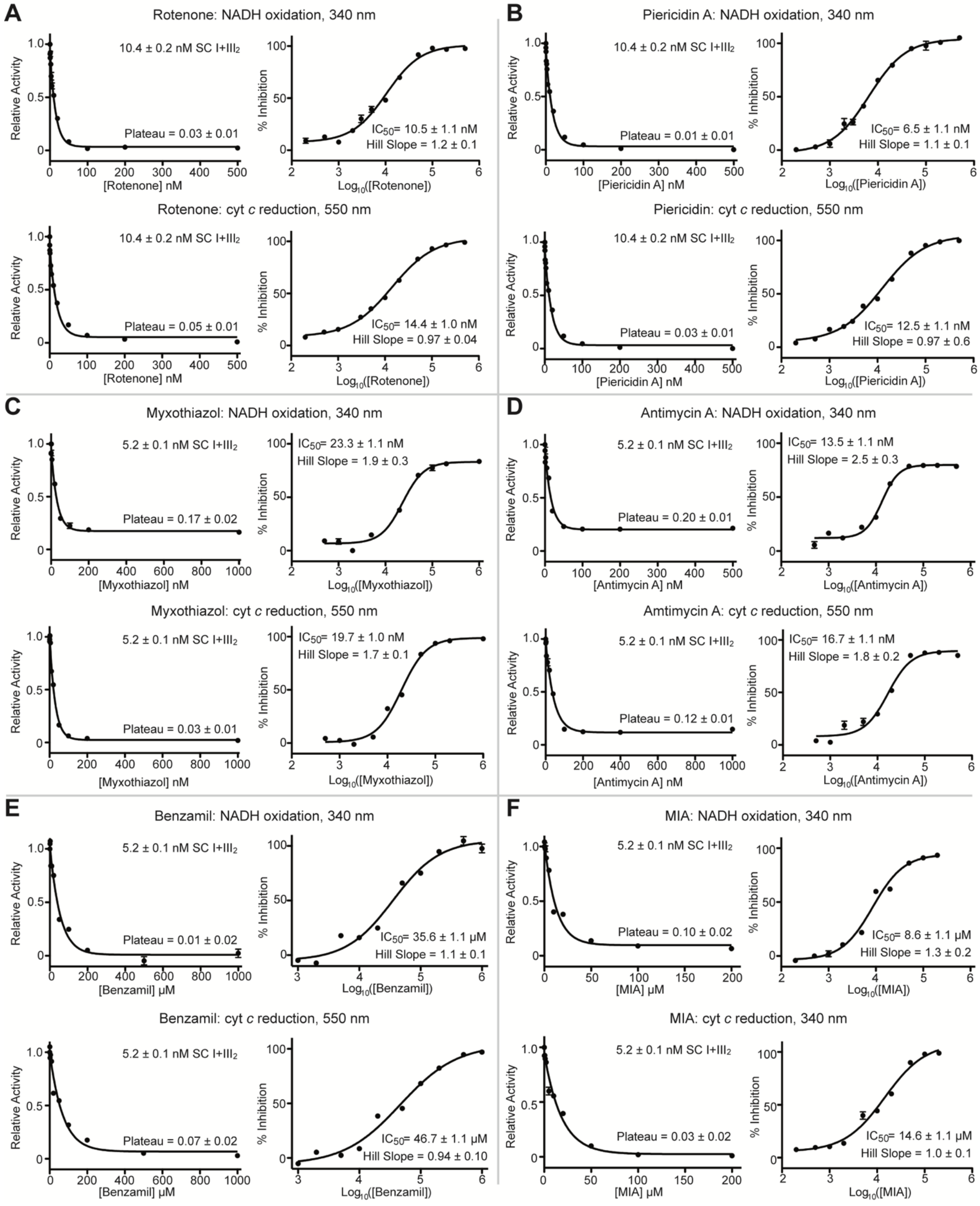
A
Rotenone: NADH oxidation, 340 nm
10.4 ± 0.2 nM SC I+III2
Plateau = 0.03 ± 0.01
[Rotenone] nM
IC50= 10.5 ± 1.1 nM
Hill Slope = 1.2 ± 0.1
Log10([Rotenone])
Rotenone: cyt c reduction, 550 nm
10.4 ± 0.2 nM SC I+III2
Plateau = 0.05 ± 0.01
[Rotenone] nM
IC50= 14.4 ± 1.0 nM
Hill Slope = 0.97 ± 0.04
Log10([Rotenone])
B
Piericidin A: NADH oxidation, 340 nm
10.4 ± 0.2 nM SC I+III2
Plateau = 0.01 ± 0.01
[Piericidin A] nM
IC50= 6.5 ± 1.1 nM
Hill Slope = 1.1 ± 0.1
Log10([Piericidin A])
Piericidin: cyt c reduction, 550 nm
10.4 ± 0.2 nM SC I+III2
Plateau = 0.03 ± 0.01
[Piericidin A] nM
IC50= 12.5 ± 1.1 nM
Hill Slope = 0.97 ± 0.6
Log10([Piericidin A])
C
Myxothiazol: NADH oxidation, 340 nm
5.2 ± 0.1 nM SC I+III2
Plateau = 0.17 ± 0.02
[Myxothiazol] nM
IC50= 23.3 ± 1.1 nM
Hill Slope = 1.9 ± 0.3
Log10([Myxothiazol])
Myxothiazol: cyt c reduction, 550 nm
5.2 ± 0.1 nM SC I+III2
Plateau = 0.03 ± 0.01
[Myxothiazol] nM
IC50= 19.7 ± 1.0 nM
Hill Slope = 1.7 ± 0.1
Log10([Myxothiazol])
D
Antimycin A: NADH oxidation, 340 nm
5.2 ± 0.1 nM SC I+III2
Plateau = 0.20 ± 0.01
[Antimycin A] nM
IC50= 13.5 ± 1.1 nM
Hill Slope = 2.5 ± 0.3
Log10([Antimycin A])
Amtimycin A: cyt c reduction, 550 nm
5.2 ± 0.1 nM SC I+III2
Plateau = 0.12 ± 0.01
[Antimycin A] nM
IC50= 16.7 ± 1.1 nM
Hill Slope = 1.8 ± 0.2
Log10([Antimycin A])
E
Benzamil: NADH oxidation, 340 nm
5.2 ± 0.1 nM SC I+III2
Plateau = 0.01 ± 0.02
[Benzamil] μM
IC50= 35.6 ± 1.1 μM
Hill Slope = 1.1 ± 0.1
Log10([Benzamil])
Benzamil: cyt c reduction, 550 nm
5.2 ± 0.1 nM SC I+III2
Plateau = 0.07 ± 0.02
[Benzamil] μM
IC50= 46.7 ± 1.1 μM
Hill Slope = 0.94 ± 0.10
Log10([Benzamil])
F
MIA: NADH oxidation, 340 nm
5.2 ± 0.1 nM SC I+III2
Plateau = 0.10 ± 0.02
[MIA] μM
IC50= 8.6 ± 1.1 μM
Hill Slope = 1.3 ± 0.2
Log10([MIA])
MIA: cyt c reduction, 340 nm
5.2 ± 0.1 nM SC I+III2
Plateau = 0.03 ± 0.02
[MIA] μM
IC50= 14.6 ± 1.1 μM
Hill Slope = 1.0 ± 0.1
Log10([MIA])
Relative Activity
% Inhibition

Figure S3. Inhibition of SC I+III$_2$ activities by known CI and CIII$_2$ inhibitors, Related to Figure 3

Relative inhibition vs. concentration (left) and percent inhibition vs. $\log_{10}$([inhibitor]) (right) are shown for inhibition of both NADH-oxidation (top) and cyt *c*-reduction (bottom) for each inhibitor. Inhibition was measured with either ~10 nM or ~5 nM SC I+III$_2$ as indicated for each panel. (A) The CI inhibitor rotenone gave an IC_{50} of 10.5 ± 1.1 nM for NADH oxidation and 14.4 ± 1.0 nM for cyt *c* reduction, with Hill coefficients of 1.2 ± 0.1 and 0.97 ± 0.04 respectively, consistent with the single known rotenone binding site. (B) The CI inhibitor piericidin A gave an IC_{50} of 6.5 ± 1.1 nM for NADH oxidation and 12.5 ± 1.1 nM for cyt *c* reduction, with Hill coefficients of 1.1 ± 0.1 and 0.97 ± 0.6 respectively. (C) The CIII$_2$ Q_P-site inhibitor myxothiazol inhibited 97 % of the cyt *c*-reduction activity at 1 μM with an IC_{50} of 19.7 ± 1.0 nM. However, myxothiazol only inhibited ~83 % of the NADH-oxidation activity, indicating that slow oxidation of NADH is possible under these conditions likely via exchange of DQH_2 and DQ between the SC particles and bulk pool (**Figure 3**L). The IC_{50} for the NADH-oxidation activity is consistent with that for the cyt *c*-reduction activity, at 23.3 ± 1.1 nM and both activities show consistent Hill coefficients of 1.9 ± 0.3 and 1.7±0.1, respectively. (D) The CIII$_2$ Q_N-site inhibitor antimycin A. Although strong inhibition is observed, neither the NADH-oxidation nor the cyt *c*-reduction activities show complete inhibition. Similar to myxothiazol, antimycin A inhibits ~80 % of the NADH-oxidation activity at 500 nM antimycin A, with IC_{50} of 13.5 ± 1.1 nM and a Hill coefficient of 2.5 ± 0.3. Antimycin A blocks less of the cyt *c*-reduction activity compared to myxothiazol, with ~88 % inhibition at 1 μM antimycin A, but the IC_{50} of 16.7 ± 1.1 nM and Hill coefficient of 1.8 ± 0.2 are consistent with the inhibition of NADH-oxidation. Inhibition of CIII$_2$ with antimycin A is known to result in increased ROS production at the Q_P site. Due to the large amount of SOD present in our reaction buffer (50 U/mL), it is unlikely that the source of residual cyt *c* reduction would be via direct reduction of cyt *c* by superoxide. More likely an electron from $CoQH_2$ is able to transfer through CIII$_2$, via UQCRFS1 and CYC1, to cyt *c*, reducing cyt *c* at the native CYC1 binding site. The $CoQ^{\bullet-}$ thus generated at the Q_P-site would be converted back to CoQ through reaction with O_2. However, more experiments are needed to confirm this hypothesis. One limitation of our isolated system, due to the lack of sealed membrane, is the absence of any membrane potential ($\Delta\psi$) or proton gradient (ΔpH). Therefore, although we can measure the oxidase activity of CI we are unable to measure its coupled H^+-pumping activity. This precludes direct demonstration that CI in the amphipol-stabilized SC I+III$_2$ maintains strong coupling between electron transfer and H^+-pumping. However, coupling can be indirectly measured using known amiloride Na^+/H^+-antiporter inhibitors that also inhibit CI. (E) The ameloride inhibitor benzamil gave an IC_{50} for NADH-oxidation of 35.6 ± 1.1 μM and (F) the ameloride inhibitor MIA gave an IC_{50} of 8.6 ± 1.1 μM. While these data are indicative of coupling in CI, they must be interpreted with caution as the binding site of the amilorides has not been definitively determined. Data are represented as mean ± SEM.

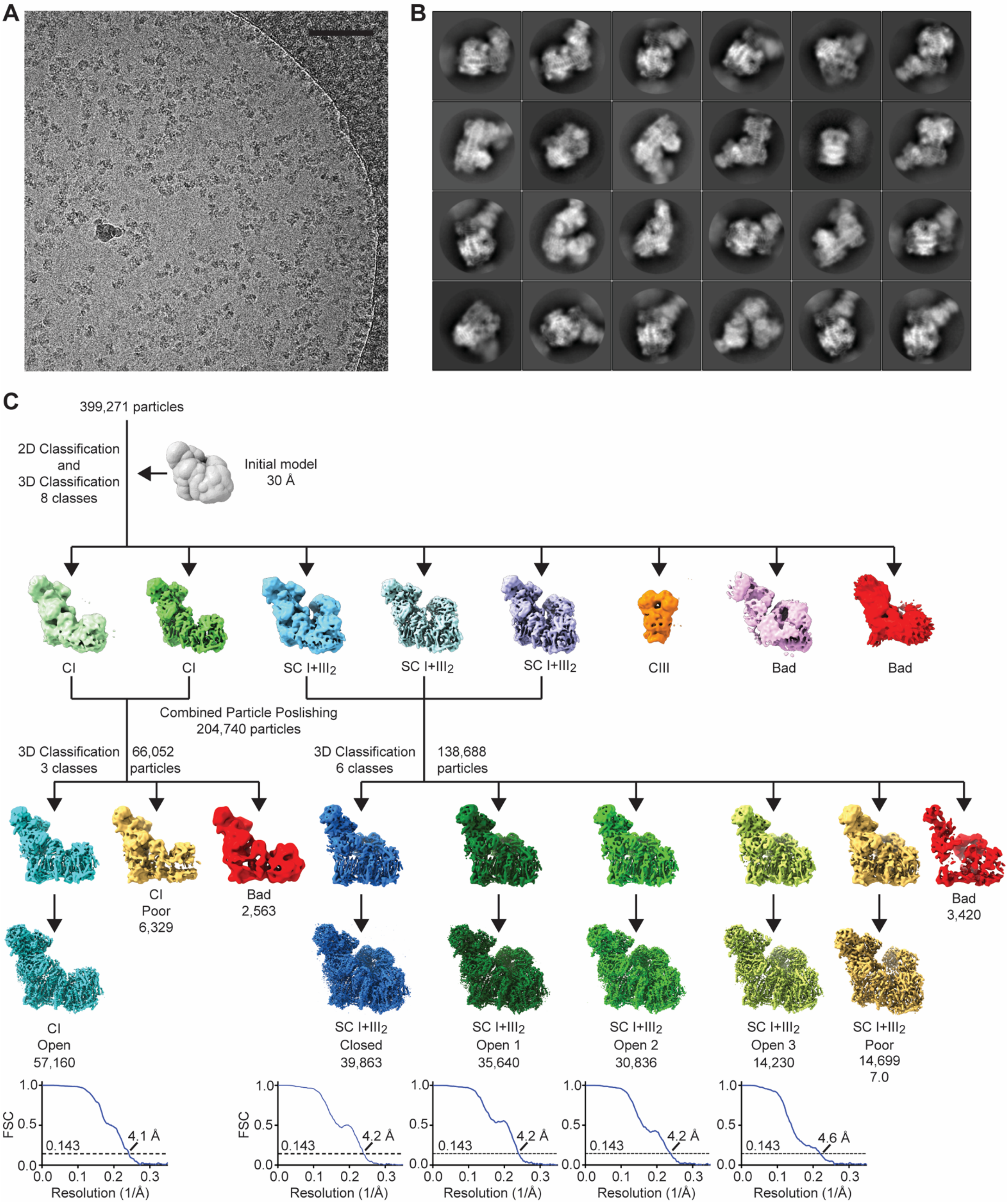
A
B
C
399,271 particles
2D Classification
and
3D Classification
8 classes
Initial model
30 Å
CI
CI
SC I+III2
SC I+III2
SC I+III2
CIII
Bad
Bad
Combined Particle Poslishing
204,740 particles
3D Classification
3 classes
66,052
particles
3D Classification
6 classes
138,688
particles
CI
Poor
6,329
Bad
2,563
Bad
3,420
CI
Open
57,160
SC I+III2
Closed
39,863
SC I+III2
Open 1
35,640
SC I+III2
Open 2
30,836
SC I+III2
Open 3
14,230
SC I+III2
Poor
14,699
7.0
FSC
0.143
4.1 Å
4.2 Å
4.2 Å
4.2 Å
4.6 Å
Resolution (1/Å)

Figure S4. Initial cryo-EM processing steps, Related to Figure 4

(A) Representative micrograph of the 1,788 micrographs collected. Scale bar is 100 nm (B) Representative 2D class averages obtained from reference-free classification in Relion (Kimanius et al., 2016). (C) Initial classification and refinement procedures used in this study. The gold-standard FSC curves are shown below their respective final reconstructions.

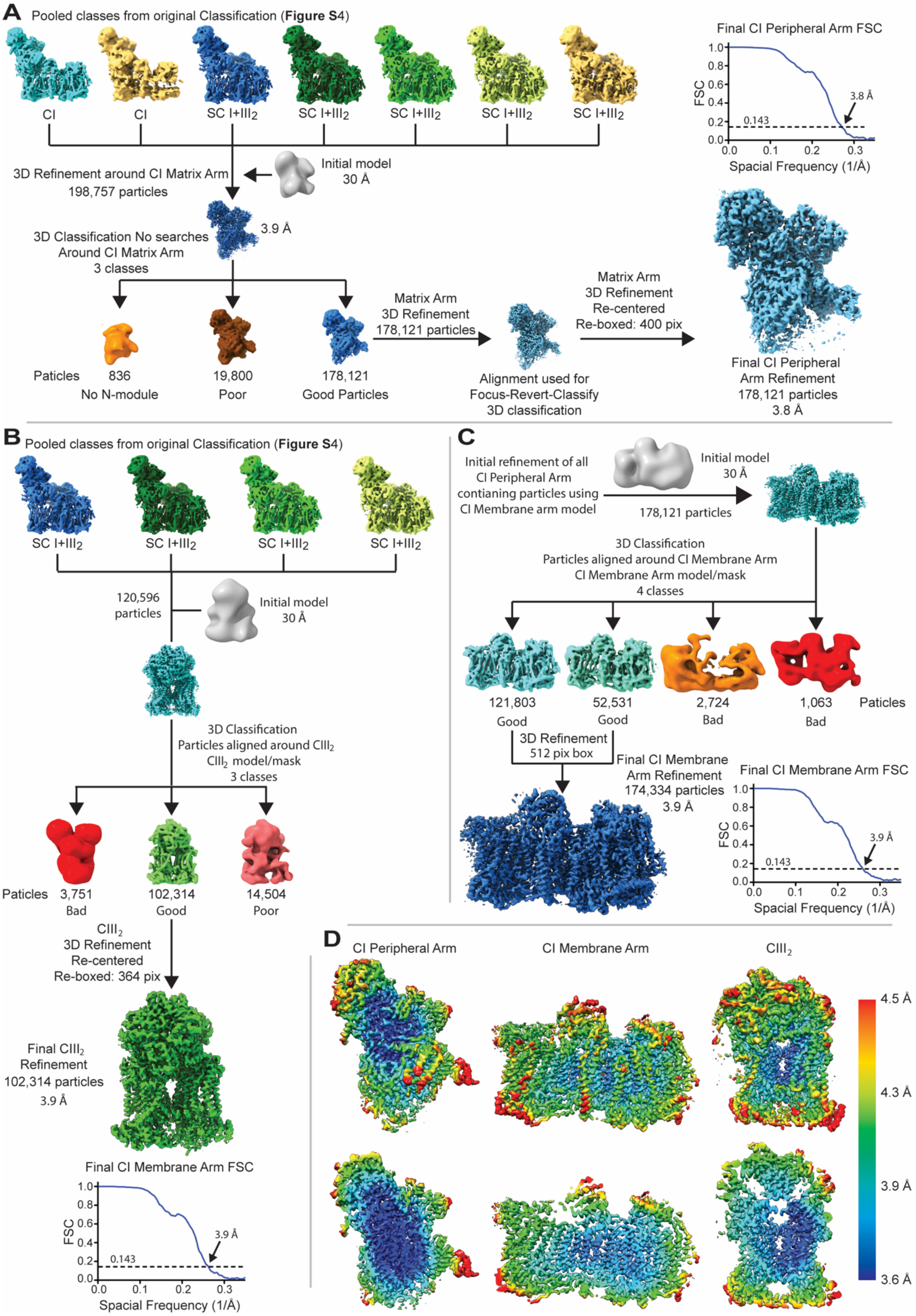
A
Pooled classes from original Classification (Figure S4)
CI
CI
SC I+III2
SC I+III2
SC I+III2
SC I+III2
SC I+III2
Initial model
30 Å
3D Refinement around CI Matrix Arm
198,757 particles
3.9 Å
3D Classification No searches
Around CI Matrix Arm
3 classes
Paticles
836
No N-module
19,800
Poor
178,121
Good Particles
Matrix Arm
3D Refinement
178,121 particles
Alignment used for
Focus-Revert-Classify
3D classification
Matrix Arm
3D Refinement
Re-centered
Re-boxed: 400 pix
Final CI Peripheral Arm FSC
FSC
1.0
0.8
0.6
0.4
0.2
0.0
0.143
3.8 Å
0.0
0.1
0.2
0.3
Spacial Frequency (1/Å)
Final CI Peripheral
Arm Refinement
178,121 particles
3.8 Å
B
Pooled classes from original Classification (Figure S4)
SC I+III2
SC I+III2
SC I+III2
SC I+III2
120,596
particles
Initial model
30 Å
3D Classification
Particles aligned around CIII2
CIII2 model/mask
3 classes
Paticles
3,751
Bad
102,314
Good
14,504
Poor
CIII2
3D Refinement
Re-centered
Re-boxed: 364 pix
Final CIII2
Refinement
102,314 particles
3.9 Å
Final CI Membrane Arm FSC
FSC
1.0
0.8
0.6
0.4
0.2
0.0
0.143
3.9 Å
0.0
0.1
0.2
0.3
Spacial Frequency (1/Å)
C
Initial refinement of all
CI Peripheral Arm
contianing particles using
CI Membrane arm model
Initial model
30 Å
178,121 particles
3D Classification
Particles aligned around CI Membrane Arm
CI Membrane Arm model/mask
4 classes
121,803
Good
52,531
Good
2,724
Bad
1,063
Bad
Paticles
3D Refinement
512 pix box
Final CI Membrane
Arm Refinement
174,334 particles
3.9 Å
Final CI Membrane Arm FSC
FSC
1.0
0.8
0.6
0.4
0.2
0.0
0.143
3.9 Å
0.0
0.1
0.2
0.3
Spacial Frequency (1/Å)
D
CI Peripheral Arm
CI Membrane Arm
CIII2
4.5 Å
4.3 Å
3.9 Å
3.6 Å

Figure S5. Focused refinements, Related to STAR Methods

(A) All CI-containing particles from the initial classifications (Figure S4) were pooled and aligned by the CI peripheral arm. An additional round of 3D-classification around the CI peripheral arm was performed to remove any remaining poorly aligning particles. The final set of 178,121 particles was then aligned, followed by re-centering and re-boxing of the particles focused on the CI peripheral arm using REP (D'Imprima et al., 2017). The final 3D-reconstruction had a nominal resolution of 3.8 Å according to the gold-standard FSC method. (B) All good $CIII_2$ containing particles (SC) from the initial 3D-classifiactions (Figure S4C) were combined and aligned around $CIII_2$. An additional round of 3D classification was performed in order to remove any remaining poorly aligning particles. The final set of 102,314 particles was then aligned, followed by re-centering and re-boxing of the particles focused on $CIII_2$ peripheral arm using REP. All alignments were performed locally, so that overall orientation of $CIII_2$ *vs* SC was preserved. The final 3D-reconstruction had a nominal resolution of 3.9 Å according to the gold-standard FSC method. (C) The set of good CI peripheral arm containing particles were aligned by the CI membrane arm and then further classified to remove any remaining poorly aligning particles. The good CI membrane arm classes were combined into a final set containing 174,334 particles; re-centering failed to improve resolution or density features and hence was not performed for the final reconstruction. The final CI membrane arm reconstruction had a nominal resolution of 3.9 Å according to the gold-standard FSC method. (D) Local resolution maps of the CI peripheral arm (left), membrane arm (middle) and $CIII_2$ (right) showing side views (top) and slices into the protein cores (bottom).

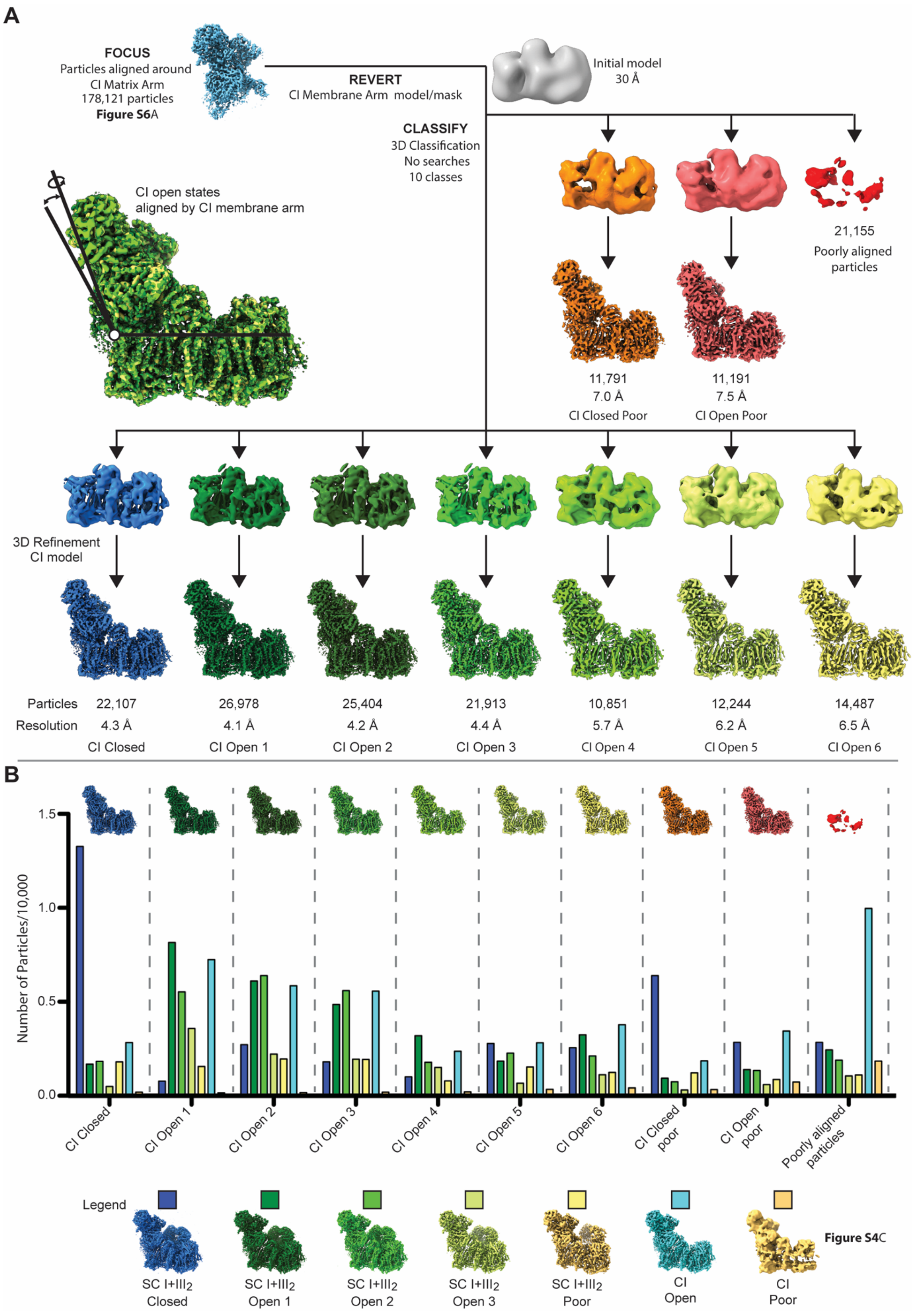

A
FOCUS
Particles aligned around
CI Matrix Arm
178,121 particles
Figure S6A
REVERT
CI Membrane Arm model/mask
Initial model
30 Å
CLASSIFY
3D Classification
No searches
10 classes
CI open states
aligned by CI membrane arm
21,155
Poorly aligned
particles
11,791
7.0 Å
CI Closed Poor
11,191
7.5 Å
CI Open Poor
3D Refinement
CI model
Particles
Resolution
22,107
4.3 Å
CI Closed
26,978
4.1 Å
CI Open 1
25,404
4.2 Å
CI Open 2
21,913
4.4 Å
CI Open 3
10,851
5.7 Å
CI Open 4
12,244
6.2 Å
CI Open 5
14,487
6.5 Å
CI Open 6
B
Number of Particles/10,000
1.5
1.0
0.5
0.0
CI Closed
CI Open 1
CI Open 2
CI Open 3
CI Open 4
CI Open 5
CI Open 6
CI Closed poor
CI Open poor
Poorly aligned particles
Legend
SC I+III2 Closed
SC I+III2 Open 1
SC I+III2 Open 2
SC I+III2 Open 3
SC I+III2 Poor
CI Open
CI Poor
Figure S4C

Figure S6. Focus-Revert-Classify strategy to separate different classes of CI, Related to Figure 5

(A) Particles aligned by the CI peripheral arm (**Figure S5**A) were classified into 10 classes using a mask for the CI membrane arm without rotational or translational searches. This resulted in the separation of seven classes of CI, which differed mainly in the angle between the membrane arm and peripheral arms (inset). The nominal resolution of these classes ranged from 4.1-6.5 Å according to the gold-standard FSC method. (B) Comparison of the original SC and isolated CI classes (**Figure 4**C) to focus-revert-classify classes with a histogram of particle counts shared between the original classes (see legend schematic) and each focus-revert-classify CI class (top, colored as in A).

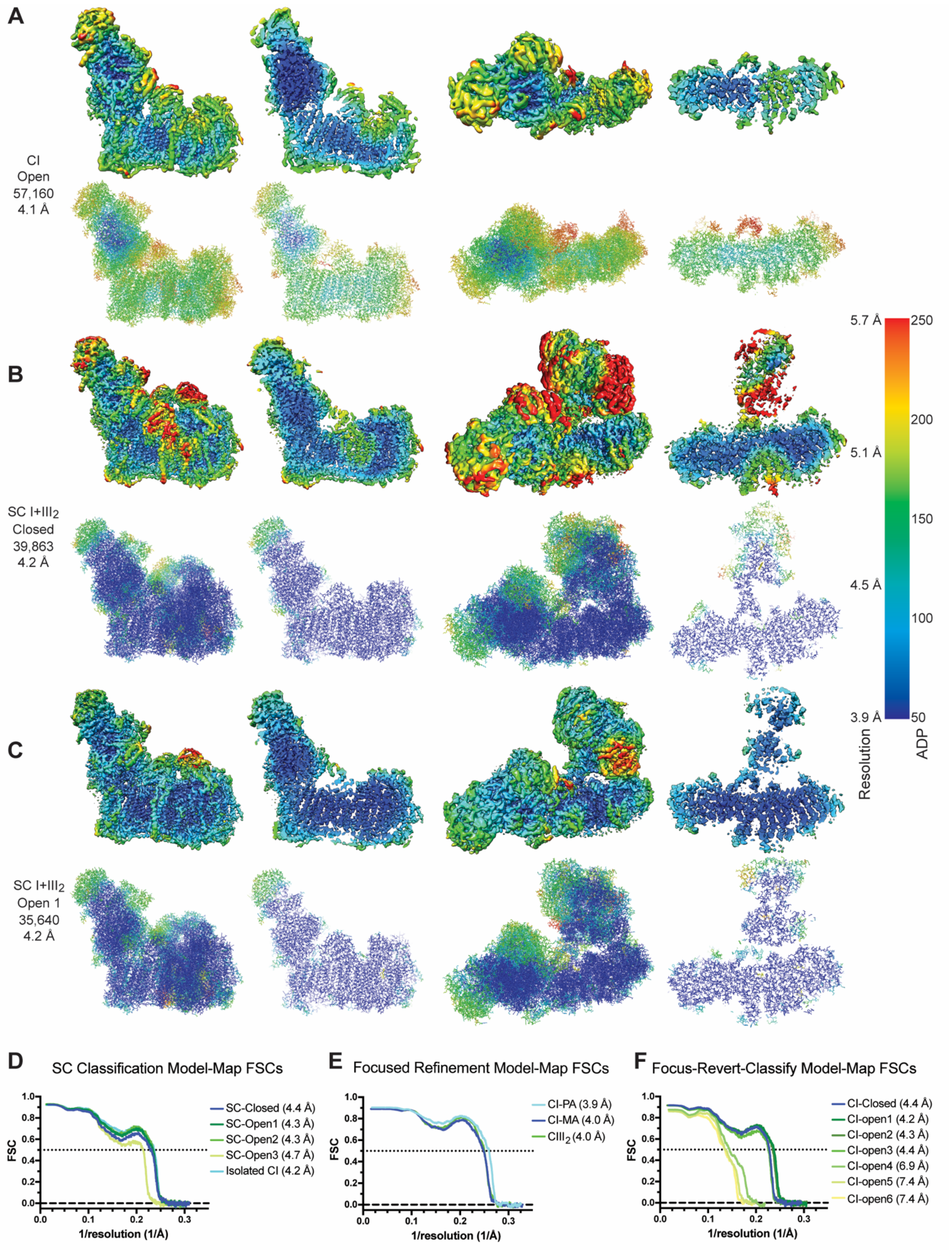
A
CI
Open
57,160
4.1 Å
B
SC I+III2
Closed
39,863
4.2 Å
C
SC I+III2
Open 1
35,640
4.2 Å
5.7 Å
5.1 Å
4.5 Å
3.9 Å
Resolution
250
200
150
100
50
ADP
D
SC Classification Model-Map FSCs
SC-Closed (4.4 Å)
SC-Open1 (4.3 Å)
SC-Open2 (4.3 Å)
SC-Open3 (4.7 Å)
Isolated CI (4.2 Å)
E
Focused Refinement Model-Map FSCs
CI-PA (3.9 Å)
CI-MA (4.0 Å)
CIII2 (4.0 Å)
F
Focus-Revert-Classify Model-Map FSCs
CI-Closed (4.4 Å)
CI-open1 (4.2 Å)
CI-open2 (4.3 Å)
CI-open3 (4.4 Å)
CI-open4 (6.9 Å)
CI-open5 (7.4 Å)
CI-open6 (7.4 Å)
FSC
1/resolution (1/Å)

Figure S7. Examples of match between local resolution and model ADPs for isolated CI and SC I+III_2, Related to Figure 7 and STAR Methods

(A) Local resolution maps (top) and refined models (bottom) for isolated CI. Side view and slice through CI (left) and matrix view and slice through membrane domain (right) are shown. (B) As in (A) for the SC I+III_2 closed state structure. (C) As in (A) for the SC I+III_2 open 1 state structure. The maps are colored by local resolution and the models are shown as sticks and colored by ADP. (D) FSC curves between the models and final maps for the initial SC classification (see **Figure S**4). (E) FSC curves between the models and final maps for the focused refinements (see **Figure S**5). (F) FSC curves between the models and final maps for the focus-revert-classify (FRC) classes (see **Figure S**6). The resolution at FSC = 0.5 is shown for each curve in the legend as well as in **Table** 3. Models for the low-resolution (>5Å) FRC classes (CI-open4, CI-open6, and CI-open7) are all poly-alanine and were only fit into the maps as rigid bodies without additional refinement.

SUPPLEMENTARY TABLES

Table S1. Mass Spectrometry identification for subunits of ovine SC I+III_2, CI and $CIII_2$, Related to Figure 1

Subunit		Mature subunit size		SC I+III_2				CI				$CIII_2$			
Bovine name	Human name	#aa	MW (kDa)	# unique peptides	% coverage	% total spectrum	Slice with highest spectrum count	# unique peptides	% coverage	% total spectrum	Slice with highest spectrum count	# unique peptides	% coverage	% total spectrum	Slice with highest spectrum count
CI core subunits															
ND1	ND1	318	35.9	6	15	0.04	18	4	11	0.04	18	3[a]	11	0.008	18
ND2	ND2	347	39.1	2	8	0.007	18	4	19	0.02	17	3	12	0.01	17
ND3	ND3	115	13.1	1	13	0.03	27	1	13	0.02	27	1	13	0.003	27
ND4	ND4	459	52	6	18	0.04	14	6	17	0.05	14	2	6	0.006	14
ND4L	ND4L	98	10.8	-[b]	-	-	-	-	-	-	-	-	-	-	-
ND5	ND5	606	68.4	17	31	0.19	11	13	28	0.26	11	12	19	0.08	12
ND6	ND6	175	19.1	3	18	0.02	25	2	5	0.02	25	1	5	0.009	25
75 kDa	NDUFS1	704	76.9	49	77	1.5	4	41	59	1.6	4	27	42	0.51	4
49 kDa	NDUFS2	430	49.1	23	51	0.32	10	20	45	0.52	10	19	37	0.13	10
30 kDa	NDUFS3	228	26.4	21	65	0.30	18	16	55	0.33	18	12	40	0.18	18
PSST	NDUFS7	179	20.1	8	36	0.26	22	7	36	0.27	22	4	18	0.07	21
TYKY	NDUFS8	176	20.2	11	51	0.12	21	7	42	0.11	21	7	42	0.05	21
51 kDa	NDUFV1	444	48.5	33	82	0.59	9	24	60	0.67	9	45	30	0.17	8
24 kDa	NDUFV2	217	23.8	15	68	0.14	19	13	58	0.17	20	11	56	0.098	20
CI Supernumerary Subunits															
MWFE	NDUFA1	70	8.2	3	36	0.007	30	2	34	0.002	30	-	-	-	-
B8	NDUFA2	98	10.9	7	43	0.11	29	7	43	0.14	29	6	43	0.08	29
B9	NDUFA3	83	9.2	5	87	0.07	30	2	22	0.06	30	2	22	0.03	29
B13	NDUFA5	115	13.1	7	72	0.09	28	4	31	0.06	29	4	31	0.06	28
B14	NDUFA6	127	14.9	7	39	0.06	27	8	51	0.10	27	4	33	0.05	27
B14.5a	NDUFA7	112	12.4	10	54	0.13	27	10	54	0.15	27	6	53	0.07	27
PGIV	NDUFA8	171	20	8	52	0.14	24	2	43	0.061	24	7	14	0.16	23
39 kDa	NDUFA9	344	39	23	59	0.48	14	24	59	0.64	14	21	56	0.22	14
42 kDa	NDUFA10	320	36.8	24	68	0.39	12	22	58	0.42	12	15	42	0.18	12
B14.7	NDUFA11	140	14.6	7	64	0.11	27	7	64	0.11	27	3	30	0.02	27
B17.2	NDUFA12	145	17.1	15	88	0.30	25	11	66	0.19	26	7	41	0.05	25
B16.6	NDUFA13	143	16.6	15	79	0.36	26	13	79	0.18	26	9	50	0.10	25
SDAP	NDUFAB1	88	10.1	4	36	0.01	30	2	24	0.003	30	-	-	-	-
MNLL	NDUFB1	57	6.9	1	30	0.002	31	1	30	0.001	31	-	-	-	-
AGGG	NDUFB2	72	8.5	2	8	0.003	29	2	8	0.003	29	1	7	0.001	Am[c]
B12	NDUFB3	97	11	4	45	0.06	28	3	28	0.02	29	3	28	0.02	28
B15	NDUFB4	128	15	11	70	0.11	26	11	70	0.08	27	7	51	0.05	26
SGDH	NDUFB5	143	16.7	10	37	0.17	25	9	37	0.21	25	6	26	0.07	25
B17	NDUFB6	127	15.4	7	51	0.07	24	7	45	0.07	24	3	29	0.01	24
B18	NDUFB7	136	16.3	11	72	0.17	24	10	60	0.23	24	9	59	0.05	23
ASHI	NDUFB8	158	18.8	9	60	0.07	24	7	53	0.1	23	5	44	0.01	23
B22	NDUFB9	178	21.6	8	56	0.13	20	5	28	0.06	21	4	20	0.05	20
PDSW	NDUFB10	175	20.8	11	59	0.19	21	11	54	0.19	20 (21)	9	53	0.13	21
ESSS	NDUFB11	125	14.4	8	52	0.20	24	6	45	0.15	24	3	23	0.03	24
KFYI	NDUFC1	49	5.8	1	20	0.006	30	1	20	0.001	29	1	20	0.004	31

B14.5b	NDUFC2	120	14.2	9	60	0.09	26	8	59	0.12	27	5	32	0.05	26
18 kDa (AQDQ)	NDUFS4	133	15.3	11	62	0.20	25	7	46	0.21	25	5	41	0.03	25
15 kDa (PFFD)	NDUFS5	105	12.4	6	47	0.05	27	6	44	0.06	27	3	14	0.007	26
13 kDa	NDUFS6	96	10.6	8	79	0.09	28	4	44	0.02	27	6	54	0.01	28
10 kDa	NDUFV3	75	8.4	1	23	0.002	29	1	23	0.007	29	1	23	0.002	28
CIII$_2$															
Core 1	UQCRC1	446	49.3	27	59	1.0	9	18	46	0.34	9	28	57	1.6	9
Core 2	UQCRC2	439	46.6	31	61	0.69	11	23	59	0.39	11	34	91	1.2	11
Cyt *b*	MT-CYB	379	42.8	2	9	0.02	16	1	2	0.004	16	2	5	0.07	16
Cyt *c1*	CYC1	241	27.3	12	39	0.18	18	7	32	0.12	16	12	43	0.84	17
Rieske	UQCRFS1	196	21.6	14	30	0.25	19	10	29	0.09	19	15	35	0.42	20
14 kDa	UQCRB	110	13.4	11	57	0.22	27	9	57	0.13	27	11	57	0.53	27
QP-C	UQCRQ	81	9.6	7	84	0.05	28	1	16	0.03	Am[c]	9	78	0.25	28
11 kDa	UQCRH	78	9.2	4	38	0.008	28	-	-	-	-	4	48	0.02	28
8 kDa	Subunit 11	78	7.9				31				31				31
7.2 kDa	UQCR10	63	7.3	3	50	0.04	31	2	38	0.04	31	4	52	0.06	31
6.4 kDa	UQCR11	56	6.5	1	21	0.001	31	-	-	-	-	1	21	0.002	31

[a]Subunits that are not expected in the sample are shown with data in grey, but in most cases are still detectable at lower levels.
[b]A dash indicates that no peptides were detected for the subunit.
[c]Ambiguous

Table S2. Lipid content of isolated SC I+III$_2$ particles compared to mitochondrial membranes, Related to STAR methods

SC I+III$_2$ Preparation	[Protein] mg/mL (n = 3-6)	[SC I+III$_2$] μM	[PO_4]$_{org}$ μM (n = 3-6)	[Lipid] μM[a]	nmole Lipid / mg Protein	Lipid molecules / SC I+III$_2$
1	9.1 ± 0.3[b]	6.3 ± 0.2	874 ± 85	728 ± 71	80 ± 8	116 ± 12
2	8.1 ± 0.4	5.6 ± 0.3	897 ± 55	748 ± 45	92 ± 7	133 ± 11
3	12.5 ± 0.3	8.6 ± 0.2	1298 ± 49	1082 ± 41	87 ± 4	126 ± 6
4	16.0 ± 0.2	11.0 ± 0.1	1537 ± 25	1281 ± 21	80 ± 2	116 ± 2
5	10.5 ± 0.6	7.2 ± 0.4	1023 ± 80	853 ± 67	81 ± 8	118 ± 12
				**Average:**	**84 ± 6**	**122 ± 8**
Suspended Mitochondrial Membranes	14.1 ± 0.6	---	2597 ± 198	2164 ± 165	153 ± 13	---

[a]Assuming ratio of 4:1 phospholipid:cardiolipin
[b]Mean ± SEM, throughout

Table S3. K_{cat} for CI and $CIII_2$ within the SC and Rate Coupling, Related to Figures 2 and 3

Condition	K_{cat} (s^{-1}) NADH oxidized per CI	Cyt *c* reduced per $CIII_2$	$CoQH_2$ oxidized per $CIII_2$	Rate coupling[a] (cyt *c* reduced / NADH oxidized)
No DQ	9.9 ± 0.2	17.9 ± 0.2	8.9 ± 0.1	1.80 ± 0.05
10 μM DQ	53.4 ± 1.5	102 ± 2	50.8 ± 0.7	1.90 ± 0.06
100 μM DQ	45.2 ± 1.2	80.3 ± 1.7	40.1 ± 0.8	1.78 ± 0.06
100 μM DQ, 10 μM BSA	59.0 ± 1.5	70.6 ± 3.4	35.3 ± 1.7	1.20 ± 0.06
100 μM DQ, Lipid-Detergent (LD) buffer	188 ± 3	226 ± 2	113 ± 1	1.21 ± 0.02
Ratio of activity LD-buffer/10 μM DQ	3.52 ± 0.11	2.23 ± 0.04	1.11 ± 0.02[b]	

[a]The expected ratio for perfect rate coupling is 2.0 cyt *c* reduced / NADH oxidized

[b]This ratio was calculated between LD-buffer per Q_P site and 10 μM DQ per $CIII_2$ indicating that the increase in $CIII_2$ activity in LD-buffer may result from increased access to the $CIII_2$ active sites already present

Table S4. Cryo-EM data collection, Related to Figure 4

Data Collection and processing	
Microscope	FEI Titan Krios (at eBIC, Diamond UK)
Camera	FEI Falcon II
Magnification	100,000x
Voltage (kV)	300
Electron exposure ($e^-/Å^2$)	51
Defocus range (μm)	~-1.5 to -3.0
Pixel size (Å)	1.4
Software	RELION 2.1

Table S5. SC I+III$_2$ closed model overview, Related to STAR Methods						
Subunit Name Human / Bovine	Chain ID	#aa built / #aa total	Un-modelled residues	% atomic model	TMHs	Co-factors / Notes
**CI core subunits**						
ND1	D1	318 / 318	-	100	8	-
ND2	D2	347 / 347	-	100	11	antiporter-like
ND3	D3	115 / 115	-	100	3	TMH1-TMH2 loop becomes ordered in open/closed transition
ND4	D4	459 / 459	-	100	14	antiporter-like
ND4L	4L	98 / 98	-	100	3	-
ND5	D5	606 / 606	-	100	16	antiporter-like
ND6	D6	175 / 175	-	100	5	TMH3 undergoes π-to-α helix conversion in open-closed transition
NDUFS1 / 75 kDa	S1	688 / 704	1-5, 694-704	97.7	-	N1b (2Fe[75])<br>N4 (4Fe[74]C)<br>N5 (4Fe[75]H)
NDUFS2 / 49 kDa	S2	430 / 430	-	100	-	N1a (2Fe[24])<br>β1- β2 loop undergoes conformational change in open-closed transition
NDUFS3 / 30 kDa	S3	208 / 228	1-6, 215-228	91.2	-	-
NDUFS7 / PSST	S7	156 / 179	1-23	87.1	-	-
NDUFS8 / TYKY	S8	176 / 176	-	100	-	N6a (4Fe[TY]1)
NDUFV1 / 51 kDa	V1	429 / 444	1-9, 439-444	96.6	-	N6b (4Fe[TY]2)
NDUFV2 / 24 kDa	V2	212 / 217	1-4, 217	97.7	-	FMN<br>N3 (4Fe[51])
**CI Supernumerary Subunits**						
NDUFA1 / MWFE	A1	70 / 70	-	100	1	-
NDUFA2 / B8	A2	82 / 98	1-12, 95-98	83.7	-	Thioredoxin fold
NDUFA3 / B9	A3	74 / 83	1-9	89.2	1	-
NDUFA5 / B13	A5	111 / 115	1-4	96.5	-	-
NDUFA6 / B14	A6	114 / 127	1-13	89.8	-	LYR protein
NDUFA7 / B14.5a	A7	96 / 112	73-88	85.7	-	-
NDUFA8 / PGIV	A8	171 / 171	-	100	-	-
NDUFA9 / 39 kDa	A9	298 / 344	187-194, 253-277, 332-344	86.6	-	NADPH
NDUFA10 / 42 kDa	AJ	319 / 320	320	99.7	-	Nucleoside kinase family
NDUFA11 / B14.7	AK	140 / 140	-	100	4	-
NDUFA12 / B17.2	AL	123 / 145	1-22	84.8	-	-
NDUFA13 / B16.6	AM	139 / 143	1-4	97.2	1	Identical to GRIM-19
NDUFAB1 / SDAP	AA	80 / 88	1-4, 85-88	90.9	-	acyl carrier protein, phosphopantetheine acyl-chain
NDUFAB1 / SDAP	AB	87 / 88	1	98.9	-	acyl carrier protein, phosphopantetheine acyl-chain
NDUFB1 / MNLL	B1	52 / 57	1-5	91.2	1	-
NDUFB2 / AGGG	B2	64 / 72	1-4, 69-72	88.9	1	-

NDUFB3 / B12	B3	73 / 97	1-10, 84-97	75.3	1	-
NDUFB4 / B15	B4	128 / 128	-	100	1	-
NDUFB5 / SGDH	B5	139 / 143	1-4	97.2	1	-
NDUFB6 / B17	B6	95 / 127	37-66, 126-127	74.8	1	-
NDUFB7 / B18	B7	119 / 136	120-136	87.5	-	Double Cx_9C CHC domain
NDUFB8 / ASHI	B8	157 / 158	1	99.4	1	-
NDUFB9 / B22	B9	176 / 178	1, 178	98.9	-	LYR protein
NDUFB10 / PDSW	BJ	171 / 175	1-2, 174-175	97.7	-	-
NDUFB11 / ESSS	BK	102 / 125	1-21, 124-125	81.6	1	-
NDUFC1 / KFYI	C1	46 / 49	47-49	93.9	1	-
NDUFC2 / B14.5b	C2	119 / 120	1	99.2	2	-
NDUFS4 / 18 kDa (AQDQ)	S4	126 / 133	1-7	94.7	-	-
NDUFS5 / 15 kDa (PFFD)	S5	99 / 105	100-105	94.4	-	Quadruple CX_9C 2 CHCH domains
NDUFS6 / 13 kDa	S6	89 / 96	1-7	92.7	-	Zn^{2+}
NDUFV3 / 10 kDa	V3	41 / 75	1-34	54.7	-	-
**CI Total**		8117 / 8514		95.3		
**CIII Protomer 1**						
UQCRC1 / Core 1	a1	439 / 446	1, 223-228	98.4	-	Peptidase M16 fold
UQCRC2 / Core 2	a2	414 / 439	1-19, 228-333	94.3	-	Peptidase M16 fold
MT-CYB / Cyt *b*	b1	378 / 379	1	99.7	8	Two *b*-type hemes
CYC1 / Cyt *c1*	c1	240 / 241	241	99.6	1	covalently bound *c*-type heme
UQCRFS1 / Rieske	f1	196 / 196	-	100	1	2Fe2S cluster
UQCRB / 14 kDa	d1	100 / 110	1-10	90.9	-	-
UQCRQ / QP-C	q1	73 / 81	74-81	90.1	1	-
UQCRQH / 11 kDa	h1	65 / 78	1-12, 78	83.3	-	-
UQCRFS1N / 8 kDa	x1	78		0.0	-	poly-Ala
UQCR10 / 7.2 kDa	i1	55 / 63	1-4, 60-63	87.3	1	-
**CIII Protomer 2**						
UQCRC1 / Core 1	a3	444 / 446	1, 446	99.6	-	Peptidase M16 fold
UQCRC2 / Core 2	a4	413 / 439	1-19, 228-334	94.1	-	Peptidase M16 fold
MT-CYB / Cyt *b*	b2	378 / 379	1	99.7	8	Two *b*-type hemes
CYC1 / Cyt *c1*	c2	239 / 241	1, 240	99.2	1	covalently bound *c*-type heme
UQCRFS1 / Rieske	f2	195 / 196	1	99.5	1	2Fe2S cluster
UQCRB / 14 kDa	d2	101 / 110	1-8, 110	91.8	-	-
UQCRQ / QP-C	q2	75 / 81	1, 77-81	92.6	1	-
UQCRQH / 11 kDa	h2	65 / 78	1-12, 78	83.3	-	-
UQCRFS1N / 8 kDa	x2	78		0.0	-	poly-Ala
UQCR10 / 7.2 kDa	i2	57 / 63	1-2, 59-63	90.5	1	-
**$CIII_2$ Total**		3927 / 4222		93.0		
