# Supplementary material for: Structures of Respiratory Supercomplex I+III2 Reveal Functional and Conformational Crosstalk
Source: Mol Cell. 2019 Sep 19;75(6):1131–1146.e6. doi: 10.1016/j.molcel.2019.07.022 (PMC6926478; doi:10.1016/j.molcel.2019.07.022)
Supplement: Document S2. Article plus Supplemental Information [file mmc2.pdf]

Article

Molecular Cell

Structures of Respiratory Supercomplex I+III$_2$ Reveal Functional and Conformational Crosstalk

Graphical Abstract

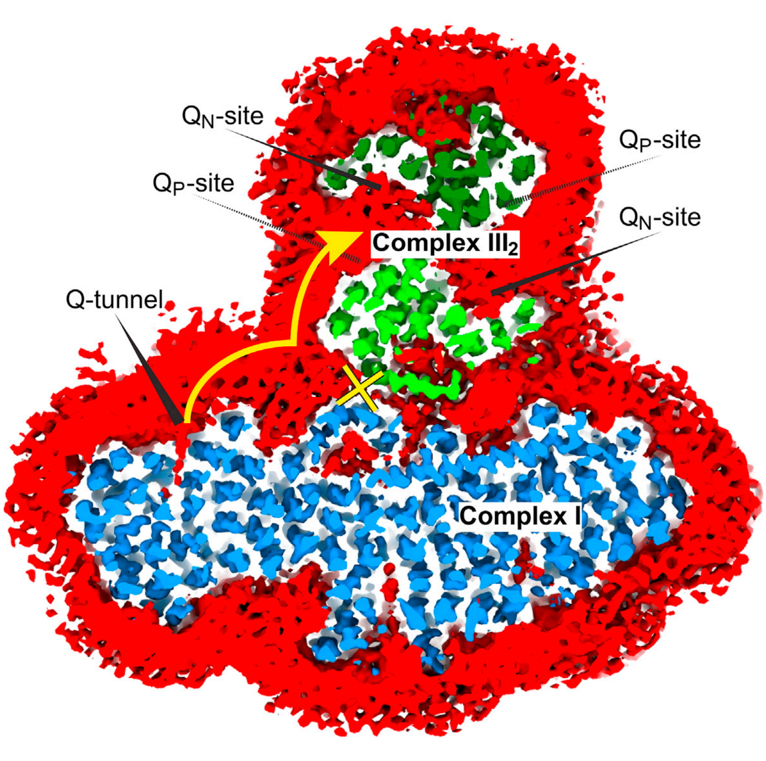

Authors

James A. Letts, Karol Fiedorczuk, Gianluca Degliesposti, Mark Skehel, Leonid A. Sazanov

Correspondence

sazanov@ist.ac.at

In Brief

The first chromatographic isolation of functional mammalian mitochondrial respiratory supercomplex I+III$_2$ (SC I+III$_2$) provides a new framework for studying the structure, function, and physiology of respiratory supercomplexes.

Highlights

- CoQ trapping within isolated respiratory supercomplex I+III$_2$ limits complex I turnover
- CryoEM structures of multiple 3D classes show crosstalk between complex I and III$_2$
- Key transmembrane helix in complex I rotates upon “closed” to “open” state transition
- CoQ density at only three complex III$_2$ sites indicates symmetry breaking

Data Resources

6Q9D 6Q9B 6QE9 6QBX 6QC3 6QC2 6QC4 6QA9 6QC5 6QC6 6QC8 6QC7 6QC9 6QCA 6QCF

Letts et al., 2019, Molecular Cell *75*, 1131–1146
September 19, 2019 © 2019 Elsevier Inc.
https://doi.org/10.1016/j.molcel.2019.07.022

CellPress

Molecular Cell
Article

CellPress

Structures of Respiratory Supercomplex I+III$_2$ Reveal Functional and Conformational Crosstalk

James A. Letts,[1,2] Karol Fiedorczuk,[1,3] Gianluca Degliesposti,[4] Mark Skehel,[4] and Leonid A. Sazanov[1,5,*]
[1]Institute of Science and Technology Austria, Klosterneuberg 3400, Austria
[2]Department of Molecular and Cellular Biology, University of California, Davis, Davis, CA 95616, USA
[3]Laboratory of Membrane Biophysics and Biology, The Rockefeller University, New York, NY 10065, USA
[4]MRC Laboratory of Molecular Biology, Cambridge CB2 OQH, UK
[5]Lead Contact
*Correspondence: sazanov@ist.ac.at
https://doi.org/10.1016/j.molcel.2019.07.022

SUMMARY

The mitochondrial electron transport chain complexes are organized into supercomplexes (SCs) of defined stoichiometry, which have been proposed to regulate electron flux via substrate channeling. We demonstrate that CoQ trapping in the isolated SC I+III$_2$ limits complex (C)I turnover, arguing against channeling. The SC structure, resolved at up to 3.8 Å in four distinct states, suggests that CoQ oxidation may be rate limiting because of unequal access of CoQ to the active sites of CIII$_2$. CI shows a transition between "closed" and "open" conformations, accompanied by the striking rotation of a key transmembrane helix. Furthermore, the state of CI affects the conformational flexibility within CIII$_2$, demonstrating crosstalk between the enzymes. CoQ was identified at only three of the four binding sites in CIII$_2$, suggesting that interaction with CI disrupts CIII$_2$ symmetry in a functionally relevant manner. Together, these observations indicate a more nuanced functional role for the SCs.

INTRODUCTION

Aerobic cellular respiration, the process by which cells transfer electrons from sugars, fats, and proteins to molecular oxygen (O_2), is central to the energy metabolism of all eukaryotes and many prokaryotes. The final stages of aerobic respiration are carried out in the mitochondria. The mitochondrial electron transport chain (ETC) complexes of the inner mitochondrial membrane (IMM) catalyze the terminal electron transfer reactions. The ETC is composed of four large membrane protein complexes: (1) a H^+-pumping NADH-coenzyme Q (CoQ; ubiquinone) oxidoreductase (complex [C]I), (2) a succinate-CoQ oxidoreductase (CII), (3) an obligatorily dimeric H^+-pumping CoQH$_2$ (reduced CoQ; ubiquinol)-cytochrome *c* (cyt *c*) oxidoreductase (CIII$_2$; cytochrome *bc$_1$* complex), and (4) a H^+-pumping cyt *c* oxidase (CIV) responsible for O_2 reduction. We now have several atomic models for all of the isolated ETC complexes (Baradaran et al., 2013; Iwata et al., 1995, 1998; Sun et al., 2005; Tsukihara et al., 1996). Most recently, structures of mammalian mitochondrial CI, the largest and least well characterized ETC complex, have been reported from multiple sources (Agip et al., 2018; Fiedorczuk et al., 2016; Guo et al., 2017; Wu et al., 2016; Zhu et al., 2016). CI is an ~1 MDa membrane protein complex with 45 protein subunits arranged into two "arms": a peripheral arm that extends into the mitochondrial matrix and a membrane arm that is embedded in the IMM (Hirst, 2013; Sazanov, 2015).

Although each ETC complex is capable of functioning in isolation (Hatefi et al., 1962), the individual complexes form supercomplexes (SCs) of defined stoichiometry (Schägger and Pfeiffer, 2000). In mammalian heart mitochondria, the majority of CI is found in association with CIII$_2$ and CIV (SC I+III$_2$+IV; respirasome) or in association with CIII$_2$ alone (SC I+III$_2$). Recent structural work has defined the arrangement of the individual complexes within the mammalian mitochondrial respirasome (Gu et al., 2016; Guo et al., 2017; Letts et al., 2016b; Sousa et al., 2016; Wu et al., 2016). Recent electron cryo-tomography on mitochondria from mammals, plants, and yeast revealed that it is not the respirasome that is structurally most conserved across kingdoms but SC I+III$_2$ (Davies et al., 2018). However, the subunits shown to be responsible for the interactions between CI and CIII$_2$ in mammalian mitochondria (Letts et al., 2016b) are either completely absent or largely truncated in plants and yeast (Letts and Sazanov, 2015; Subrahmanian et al., 2016), suggesting convergent evolution and, in turn, an important but still undefined physiological role for SC I+III$_2$ in energy metabolism (Davies et al., 2018).

The possible physiological functions of the SCs remain controversial (Letts and Sazanov, 2017; Milenkovic et al., 2017), with proposals including roles in the stability of the individual complexes (Acín-Pérez et al., 2004; Calvaruso et al., 2012; Schägger et al., 2004), CoQ substrate channeling between CI and CIII$_2$ (Bianchi et al., 2004; Lapuente-Brun et al., 2013; Lenaz et al., 2016), the reduction of reactive oxygen species (ROS) production (Lopez-Fabuel et al., 2016), and the prevention of non-specific protein aggregation in the IMM (Blaza et al., 2014). Evidence has been mounting against the hypothesis that substrate channeling is a significant function of the respirasome, especially against the notion that there are two pools of CoQ in the IMM, one associated with SCs and one freely diffusing (Enríquez, 2016; Lapuente-Brun et al., 2013). Recent respirasome structures (Gu et al., 2016; Guo

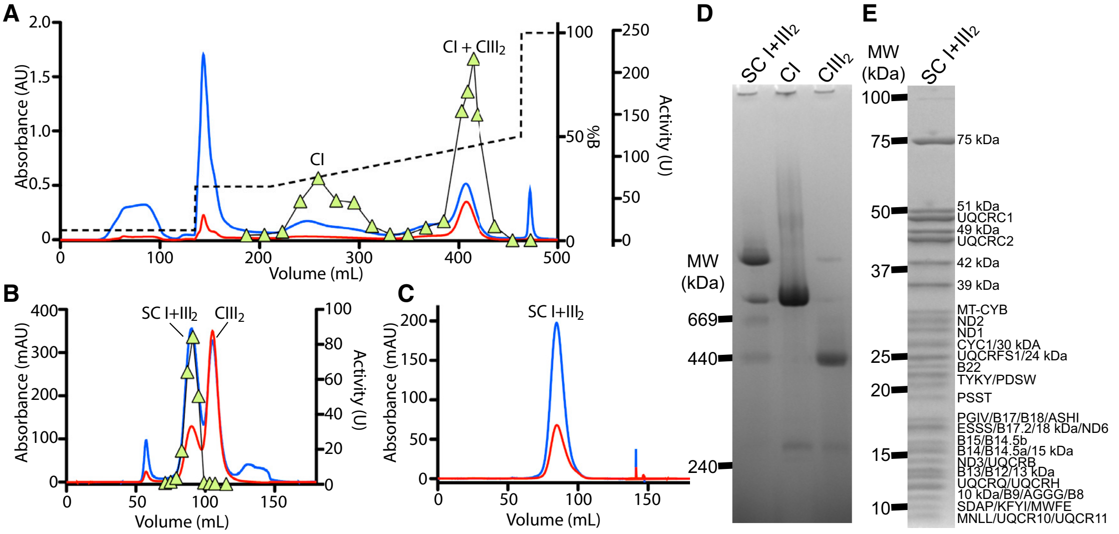

Figure 1. Purification of SC I+III$_2$ from Ovine Mitochondria

(A) Q-Sepharose anion-exchange column chromatogram of LNMG-extracted washed mitochondrial membranes. Chromatograms show A_{280} (blue line), A_{420} (red line), percentage buffer B (containing 1M NaCl) (dashed black line), and CI NADH:FeCy activity (lime green triangles) throughout. Complex (C)I elutes in two distinct peaks: the first peak is isolated CI, and the second peak is SC I+III$_2$ and excess CIII$_2$.

(B) Superose 6 size exclusion column chromatogram of amphipol-(A8-35)-exchanged SC from the second peak fraction of the anion-exchange step shown in (A) in the absence of detergent.

(C) Superose 6 size exclusion column chromatogram of amphipol-stabilized SC I+III$_2$ from peak fraction containing SC I+III$_2$ in (B).

(D) BN-PAGE gel of the purified SC I+III$_2$ with isolated CI and CIII$_2$ shown for comparison.

(E) SDS-PAGE of purified SC I+III$_2$ with labels for some subunits identified by mass spectrometry (MS). Some labels were excluded for clarity.

See also Figure S1 and Table S1.

et al., 2017; Letts et al., 2016b; Sousa et al., 2016; Wu et al., 2016) do not show any protein subunits blocking the free exchange of CoQ from CI or CIII$_2$ within the membrane pool. Moreover, kinetic analyses indicate that only a single pool of CoQ exists in the membrane (Blaza et al., 2014; Fedor and Hirst, 2018; Gupte et al., 1984; Kröger and Klingenberg, 1973a, 1973b). To settle this debate, new experimental frameworks are needed to further explore the physiological roles of SCs.

Here, we report the isolation of functional SC I+III$_2$ from ovine heart mitochondria. The preparation was highly active when isolated in amphipols, providing a soluble functional respiratory unit eminently suitable for detailed functional studies. Our results indicated that limiting amounts of CoQ-10 were co-purified with the SC and that CoQ trapping by SC particles in fact decreased rates of electron transport. Structural characterization of the SC particle by cryo-electron microscopy (cryoEM) resulted in reconstructions at 4.2–4.6 Å overall resolution of multiple structural three-dimensional (3D) classes and up to 3.8 Å resolution in focused refinements. In contrast to the isolated ovine CI (Fiedorczuk et al., 2016), subunit NDUFA11 (B14.7), the traverse helix from ND5 subunit, and a few other peripheral areas of the complex were well ordered within the SC, confirming the stabilizing role of CIII$_2$ on CI (Letts and Sazanov, 2017). The structures here allowed us to improve the completeness and accuracy of the ovine CI model and to obtain an atomic model of ovine CIII$_2$, with clear density for endogenous CoQ bound to three of the four possible sites. Comparison of local map resolution between SC structures revealed a CI state-dependent conformational flexibility in CIII$_2$'s cytochrome *b* (MT-CYB) subunit, indicating crosstalk between the two complexes. Using a focus-revert-classify strategy to separate distinct states of CI, we resolved six open and one major closed conformation. These additional states expand on the structural states previously observed in the bovine, ovine, and mouse CI (Agip et al., 2018; Blaza et al., 2018; Fiedorczuk et al., 2016; Zhu et al., 2016) and add an intriguing possibility that a key transmembrane (TM) helix rotates during the catalytic cycle. Our approach presents a new experimental framework for structure-function analysis of the physiological roles of mitochondrial SCs. Our biochemically defined system supports a stabilizing rather than a substrate-channeling function for SCs and suggests a more subtle functionally relevant interaction between CI and CIII$_2$.

RESULTS

Preparation of Chromatographically Pure SC I+III$_2$

When purifying CI, we observed that the yield from the first chromatographic step (anion exchange Q-column) was lower when the membranes were extracted with the branched chain detergent lauryl maltose neopentyl glycol (LMNG) than when using the single-chain detergent dodecyl-maltoside (DDM) (Letts et al., 2016a). In LMNG, a significant proportion of CI did not elute from the column until the high salt wash (Figure S1A). By modifying the gradient, two clear peaks of CI NADH:FeCy activity could be identified (Figures S1B and 1A).

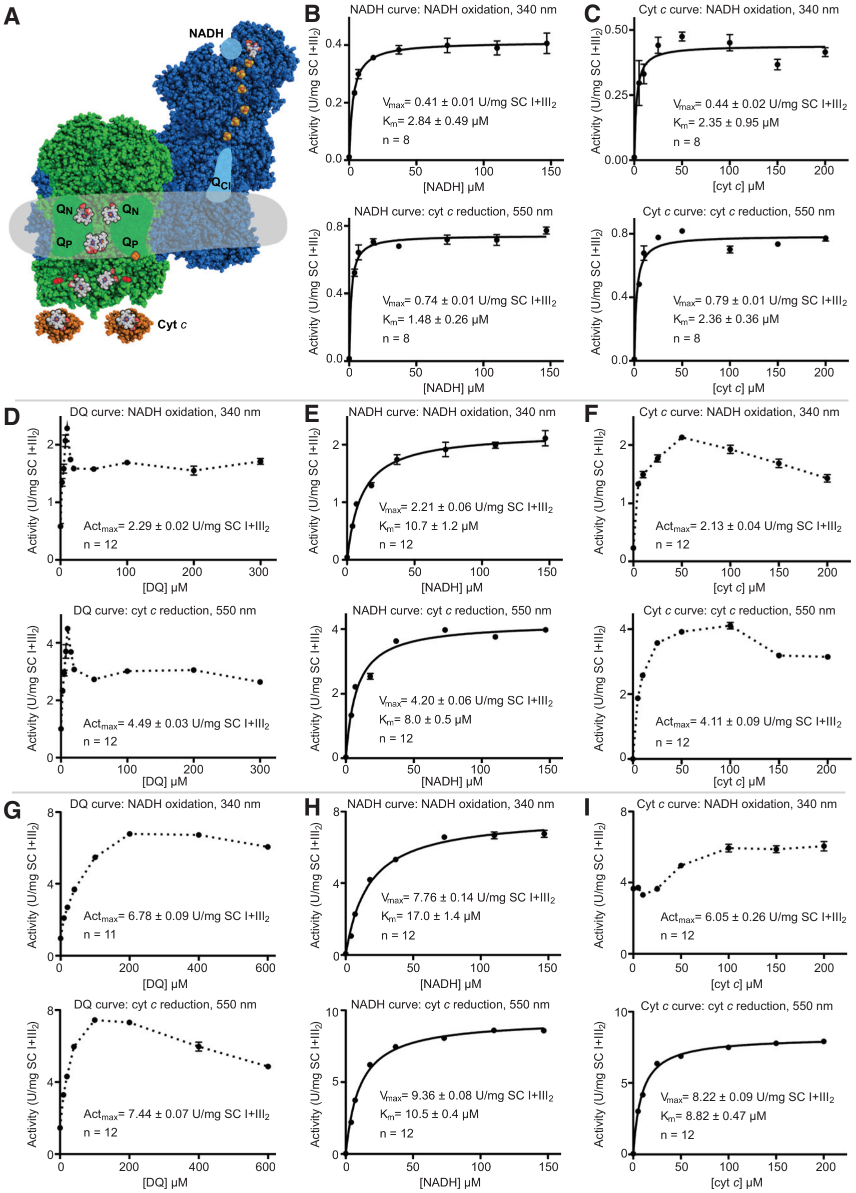

Figure 2. NADH:cyt *c* Oxidoreductase Activity of Isolated SC I+III_2

(A) Schematic shows positions of CI (blue) and $CIII_2$ (green) within the SC and the different catalytic sites. The FMN, FeS clusters, and heme groups are shown colored by atom: carbon in gray, nitrogen in blue, oxygen in red, sulfur in yellow, and iron in orange. The two major conformations of the Rieske FeS domain of the CIII subunit UQCRFS1 are indicated with Q_p-proximal in orange and c_1-proximal in red. The gray area indicates the approximate extent of the amphipol-lipid belt.

(B) [NADH]-activity curves, NADH oxidation (top), and cyt *c* reduction (bottom) throughout, in standard buffer (SB) plus 100 µM cyt *c*.

(C) [cyt *c*]-activity curves in SB plus 100 µM NADH.

(D) [DQ]-activity curve in SB plus 100 µM NADH and 100 µM cyt *c*.

(E) [NADH]-activity curves in SB plus 10 µM DQ and 100 µM cyt *c* added.

(F) [cyt *c*]-activity curves in SB plus 10 µM DQ and 100 µM NADH.

(G) [DQ]-activity curves in lipid-detergent (LD) buffer plus 100 µM NADH and 100 µM cyt *c*.

(H) [NADH]-activity curves in LD buffer plus 100 µM DQ and 100 µM cyt *c*.

(I) [cyt *c*]-activity curves in LD buffer plus 100 µM DQ and 100 µM NADH.

Data are mean ± SEM.

See also Figure S2 and Table S3.

The second peak contained both CI NADH:FeCy activity and high absorption at 420 nm, indicating the presence of heme groups (Figures S1B and 1A). When this peak was concentrated and run over either another round of anion exchange (Mono Q) or a size exclusion chromatography (SEC) column, it separated into two distinct peaks, one with NADH:FeCy activity and the other with the majority of the A_{420} signal (Figures S1C–S1E). Mass spectrometry demonstrated that these peaks corresponded to isolated CI and $CIII_2$ (Figures S1F–S1H; Table S1). By adding the amphipathic polymer (amphipol) A8-35 directly following elution of the CI + $CIII_2$ peak on the first anion exchange step, a peak containing both CI NADH:FeCy activity and A_{420} signal was isolated by SEC (Figures 1B and 1C). Blue Native (BN)-PAGE indicated that this peak was SC I+III_2 (Figure 1D). This was confirmed by mass spectrometry, which identified the presence of all expected CI and $CIII_2$ subunits (Figures 1E and 1H; Table S1).

In the absence of amphipol, the complexes dissociated over time and separated during subsequent purification steps (Figures S1C–S1E). Given that lipids are important to stabilize SCs (Mileykovskaya and Dowhan, 2014), de-lipidation is a possible cause for the separation of the SC I+III_2 during purification in the absence of amphipols. We determined that the isolated SCs were lipid-protein particles containing on average 122 ± 8 lipid molecules per SC (Table S2). Given the ~4:1 phospholipid/cardiolipin ratio of the IMM (Horvath and Daum, 2013), this suggests that each SC particle contains ~24 cardiolipin molecules and ~98 standard phospholipids, mainly phosphatidylcholine and phosphatidylethanolamine with minor amounts of phosphatidylinositol and phosphatidylserine.

Amphipol-Stabilized SC I+III_2 Is a Functional NADH:cyt *c* Oxidoreductase

The amphipol-stabilized SC I+III_2 displayed all expected enzymatic activities (see Figure 2A for a schematic of SC I+III_2 substrate binding sites), that is, CI NADH:CoQ oxidoreductase activity and $CIII_2$ $CoQH_2$:cyt *c* oxidoreductase activity via the Q-cycle mechanism (Cramer et al., 2011). Independent of whether NADH oxidation or cyt *c* reduction was monitored, clear hyperbolic concentration-activity curves for both NADH and cyt *c* were

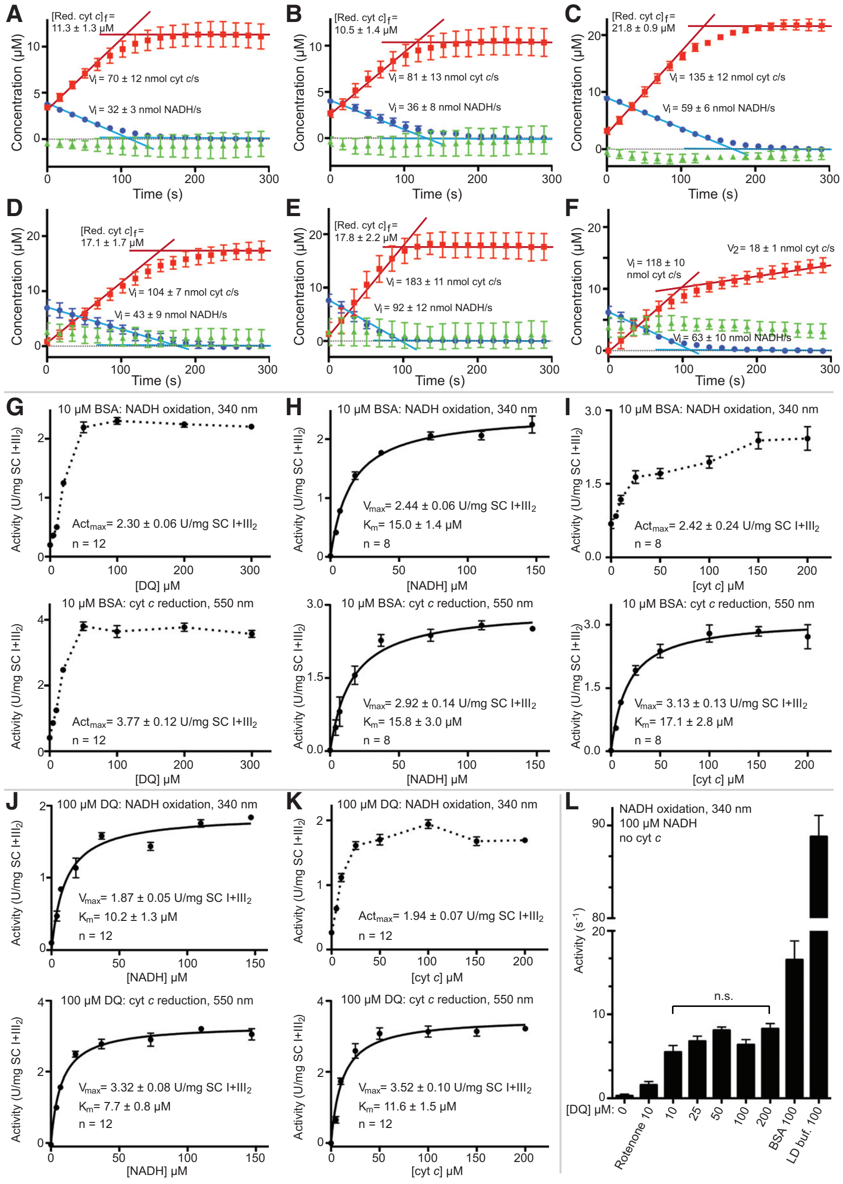

Figure 3. BSA Uncouples CI and $CIII_2$ Activity in SC I+III_2, but [DQ] Alone Has Little Effect

(A–F) SC I+III_2 activity time courses after addition of 5 μM NADH to SB plus 100 μM cyt *c* (A); 5 μM NADH to SB plus 100 μM cyt *c* and 10 μM DQ (B); 10 μM NADH to SB plus 100 μM cyt *c* and 10 μM DQ (C); 10 μM NADH to SB plus 100 μM cyt *c* and 100 μM DQ(D); 10 μM NADH to SB plus 100 μM cyt *c*, 100 μM DQ, and 10 μM BSA (E); and 10 μM NADH to LD buffer plus 100 μM cyt *c* and 100 μM DQ (F). In each panel, the [NADH] (blue circles) and [reduced cyt *c*] (red squares) are shown at each time point (n = 8, data are represented as mean ± SEM). Initial rates and final concentrations are denoted by blue lines for [NADH] and red lines for [cyt *c*] and indicted in the panels (mean ± SEM). Green triangles represent calculated values of DQH_2 at each time point assuming [DQH_2](t) = {[NADH](initial)-[NADH](t)}-{[Red. cyt *c*](t)/2} and plotted as mean ± SEM.

(G) [DQ]-activity curves, NADH oxidation (top), and cyt *c* reduction (bottom) throughout, in SB plus 10 μM BSA, 100 μM NADH, and 100 μM cyt *c*.

(H) [NADH]-activity curves in SB plus 10 μM BSA, 100 μM DQ, and 100 μM cyt *c*.

(I) [Cyt *c*]-activity curves in SB plus 10 μM BSA, 100 μM DQ, and 100 μM NADH.

(J) [NADH]-activity curves in SB plus 100 μM DQ and 100 μM cyt *c*.

(K) [Cyt *c*]-activity curves in SB plus 100 μM DQ and 100 μM NADH.

(L) NADH oxidation activity in the absence of cyt *c* at the indicated DQ concentrations in SB: rotenone, 2.5 μM rotenone; BSA, 10 μM BSA; LD buf., lipid-detergent buffer.

Data are mean ± SEM, n = 8–12. Not significant (n.s.) indicates $p > 0.01$.

See also Table S3.

seen in the absence of any added CoQ analogs (Figures 2B and 2C). This indicated that endogenous CoQ-10 co-purified with the SC particles. This co-purification is expected even in the absence of CoQ-10 trapping in the membrane, because of CoQ-10's high hydrophobicity (Persson et al., 2005) and the fact that the SCs, which contain several CoQ-10 binding sites, are being extracted into an aqueous environment.

As NADH is a two-electron donor and cyt *c* is a single-electron acceptor, two molecules of cyt *c* are reduced per NADH oxidized. The observed "rate-coupling" ratio of K_{cat}s (cyt *c* reduction/NADH oxidation) was 1.80 ± 0.05 (Table S3), near the expected ratio of 2.0 for perfect rate coupling, indicating that electrons from NADH are directly transferred to cyt *c* without a significant buildup of reduced $CoQH_2$. Time courses of NADH oxidation and cyt *c* reduction showed an expected ~2-fold greater final concentration of reduced cyt *c* relative to NADH added (Figures 3A–3F). A possible side reaction may be the reduction of cyt *c* by superoxide generated from the reduced flavin of CI (Pryde and Hirst, 2011), bypassing CoQ-10 and $CIII_2$. To prevent this, all reaction buffers contained 50 U/mL superoxide dismutase (SOD). Together, the high rate coupling and expected final concentration of reduced cyt *c* indicated that very few electrons were escaping to ROS. Nevertheless, the activity of the isolated SC I+III_2 was low relative to what was measured in ovine mitochondrial membranes or isolated mammalian mitochondrial CI using various CoQ analogs (Hatefi and Stiggall, 1978; Letts et al., 2016a; Sharpley et al., 2006; Shinzawa-Itoh et al., 2010), suggesting that the activity of the isolated SC I+III_2 may be limited by insufficient levels of co-purified CoQ-10.

To address this, we tested different concentrations of the CoQ analog decyl-ubiquinone (DQ) in the reaction buffer (Figure 2D). The addition of small amounts of DQ (~10 μM) resulted in a large increase in both NADH oxidation and cyt *c* reduction activity; however, further addition of DQ resulted in a reduction of activity

relative to the peak at 10 μM DQ (Figure 2D). The non-hyperbolic nature of the [DQ]-activity curve has been attributed to its hydrophobic "detergent-like" properties (Estornell et al., 1993; Letts et al., 2016a). If DQ solubility dictated the shape of the [DQ]-activity curve, factors such as ionic strength should affect the profile. To test this, we exchanged the buffer from HEPES to potassium phosphate, maintaining the same pH, and re-measured the concentration activity curve (Figure S2A). This buffer change resulted in a significantly different profile at low DQ concentrations, with a broader high-activity peak (5–20 μM DQ) in phosphate compared with HEPES (tightly centered around 10 μM; Figures S2B and S2C). This is consistent with the shapes of the curves resulting from solubility effects and the low activity seen at high [DQ] being due to poor DQ solubility.

Given that maximal NADH:cyt *c* oxidoreductase activity was seen with 10 μM DQ, this concentration was used to evaluate NADH and cyt *c* activity (Figures 2E and 2F). In the case of the [NADH]-activity curve, a hyperbolic profile was seen, with a higher V_{max} (~5-fold increase) and higher apparent K_m than when no DQ was added (Figures 2B, 2C, 2E, and 2F). However, although the activity was increased at all [cyt *c*] when 10 μM DQ was added, the [cyt *c*]-activity curves are no longer hyperbolic, instead showing a pronounced decrease in activity at high [cyt *c*] (Figure 2F). Given that this is not seen at higher [DQ] (see below), this reduction in activity may stem from a non-specific interaction between cyt *c* and DQ. The rate-coupling ratio from the [NADH] activity curves was 1.90 ± 0.06 (Table S3), again reflecting efficient electron transfer from NADH to cyt *c* (Figure 3B).

Although addition of DQ increased the activity of the amphipol-stabilized SC I+III_2 (Figure 2D), the NADH oxidation activity of CI was still lower than that seen in mitochondrial membranes or solubilized in detergent (Letts et al., 2016a), indicating that some aspect of SC I+III_2 was limiting CI turnover. The $CIII_2$ activity observed in SC I+III_2 (102 ± 2 s^{-1}; Table S3) is consistent with that measured for isolated eukaryotic $CIII_2$ (*S. cerevisiae*, 60–270 s^{-1} [Covian and Trumpower, 2008; Gutierrez-Cirlos and Trumpower, 2002; Nett et al., 2000], and *Bos taurus*, ~600 s^{-1} [Brandt and Okun, 1997]) and for mitochondria of living cultured cells (~250 s^{-1} [Kim et al., 2012; Ripple et al., 2013]). However, the rate of ~100 cyt *c* reduced per $CIII_2$ per second is on the low end of the reported range, suggesting that the activity of $CIII_2$ may also be impeded in the amphipol-stabilized SC.

To determine whether CI and $CIII_2$ activities are limited by confinement in the SC or whether isolation by this protocol results in lower activity in general, the activities of the amphipol-stabilized SC were assayed in lipid-detergent (LD) buffer containing 0.25 mg/mL dioleoylphosphatidylcholine (DOPC)/cardiolipin (CL) 4:1, 0.1% CHAPS (w/v), and 0.1% LMNG (w/v). This LD mixture allows maximal activity for isolated ovine CI (Letts et al., 2016a). The addition of lipid and detergent to the reaction mixture resulted in a significant shift in the [DQ] activity curve with maximal activity at higher [DQ] (~100 μM) and marked increases in both NADH oxidation and cyt *c* reduction activity (Figure 2G). The [NADH] activity curves in these conditions were hyperbolic and displayed increased V_{max} and higher apparent K_m values than when measured in the absence of lipid and detergent (Figures 2H and 2E). Addition of lipid and detergent uncoupled NADH oxidation from cyt *c* reduction and resulted in robust NADH oxidation in the absence of any added cyt *c* (Figure 2I). However, the addition of sufficient cyt *c* (≥50 μM) did increase the NADH oxidation activity of CI, indicating that $CIII_2$ activity was still able to influence CI and that their association may not be wholly disrupted (Figure 2I). When cyt *c* reduction was monitored, the [cyt *c*] activity curve was hyperbolic, resulting in a 2-fold increase in V_{max} relative to the maximum activity seen in the absence of lipid and detergent (8.22 ± 0.09 U/mg versus 4.11 ± 0.09 U/mg). This rate of ~230 cyt *c* reduced per second per $CIII_2$ agrees more closely with fluxes measured in the mitochondria of living cells (Kim et al., 2012; Ripple et al., 2013). Given that upon addition of the LD buffer, the CI activity increased by a larger factor than $CIII_2$ activity, the rate-coupling ratio decreased to 1.21 ± 0.02 (Table S3). When a time course of the reaction was examined, a mismatch between the amount of NADH oxidized versus cyt *c* reduced at each time point was observed (Figure 3F). Moreover, the reduction of cyt *c* became bi-phasic with a fast phase of reduction that coincided with the oxidation of NADH by CI and a slow phase of cyt *c* reduction after all the NADH was oxidized (Figure 3F). The two phases of cyt *c* reduction activity again suggest that the amphipol-stabilized SC I+III_2 may not be completely disrupted in the LD buffer. The initial fast phase of cyt *c* reduction likely corresponds to DQH_2 diffusing quickly within the SC between CI and $CIII_2$ and the slow phase to DQH_2 that escapes the SC into the LD buffer and then must re-encounter $CIII_2$ to be oxidized (Figure 3F). Overall, there is a buildup of reduced DQH_2 during the experiment as CI activity outpaces that of $CIII_2$. This is consistent with there being a kinetic advantage to keeping the CI and $CIII_2$ active sites close together, as would be expected for a diffusion-coupled process (Hackenbrock et al., 1986). Nonetheless, diffusion of CoQ has been shown not to be rate limiting during respiration (Chazotte and Hackenbrock, 1988), and hence decreasing the diffusion distance between CI and $CIII_2$ is not likely to be the main physiological role of SCs. Comparing the LD buffer data with those in the absence of detergent indicates that CI activity is limited in the amphipol-stabilized SC I+III_2 particle.

We also note that the NADH oxidation and cyt *c* reduction activities of the amphipol-stabilized SC I+III_2 were sensitive to known CI and $CIII_2$ inhibitors (Figure S3). We tested the CI inhibitors rotenone and piericidin A, as well as Na^+/H^+-antiporter amiloride inhibitors. Also, the Q_P-site $CIII_2$ inhibitor myxothiazol and the Q_N-site inhibitor antimycin A both showed strong inhibition of SC activities, with Hill coefficients close to 2, suggesting that binding to either of the Q_P or Q_N sites in the dimer may be sufficient to inhibit $CIII_2$ activity (Figures S3C and S3D).

CI Activity Is Limited by $CIII_2$ Activity When CoQ Is "Trapped" in SC I+III_2

To better understand what was limiting the activity of the SC, we measured SC activity at different DQ concentrations in the presence or absence of the non-specific hydrophobic carrier protein BSA (Figures 3G–3I). The addition of 10 μM BSA to the reaction mixture shifted the [DQ]-activity curve to higher [DQ], and the curves became more "hyperbolic-like" (Figure 3G). This indicates that higher concentrations of DQ are needed to make the substrate available for electron transport in the SC I+III_2

particles in the presence of BSA and hence that BSA reduces the partitioning of DQ into the SC lipid-protein particles at low [DQ]. When we measured the NADH oxidation activity in the presence of BSA, we saw a slight but significant ($p < 0.01$) increase in V_{max}, whereas there was a significant decrease in the V_{max} of cyt *c* reduction (Figures 2E, 2F, and 3H). This results in a reduction of the rate-coupling ratio similar to that seen in LD buffer (Table S3), indicating that BSA provides an alternate path for DQH_2 out of the SC I+III_2 particle. To test whether the higher concentration of DQ was partially responsible for the differences in LD buffer and in the presence of BSA, we measured [NADH] activity and [cyt *c*] activity curves with 100 μM DQ (Figures 3J and JK). As expected from the [DQ] activity curves (Figure 2D), the V_{max} measured with 100 μM DQ was lower than that using 10 μM DQ (Figures 3J and 3K). However, the decreases in NADH oxidation and cyt *c* reduction activities were proportional, thus maintaining a high rate-coupling ratio between CI and $CIII_2$ (Table S3).

Whether the ability of SC-associated DQ to exchange with the bulk DQ pool is dependent on [DQ] can also be estimated by measuring NADH oxidation in the absence of any added cyt *c* (Figure 3L). In these conditions, reduced DQH_2 cannot be readily oxidized by $CIII_2$, and thus the only source of oxidized CoQ to maintain CI activity is via exchange of DQ from the bulk pool into the SC I+III_2 particles. In the absence of DQ and cyt *c,* NADH oxidation activity was undetectable (Figures 2C and 3L). This shows that the small amount of co-purified CoQ-10 (Figures 2B and 2C) is rapidly reduced and that there is no readily available mechanism for the regeneration of oxidized CoQ-10. When 10 μM DQ was added, some NADH oxidation was maintained even in the absence of added cyt *c* (Figures 2F and 3L), indicating that the more soluble CoQ analog DQ is able to slowly exchange with the bulk pool. Surprisingly, the rate of NADH oxidation in the absence of added cyt *c* was independent of the concentration of DQ, with no significant difference in the rate of NADH oxidation over 10–200 μM DQ (Figure 3L). This low rate of NADH oxidation was nearly completely inhibited by rotenone (Figure 3L), indicating that NADH was not oxidized via ROS production at the flavin site of CI (Pryde and Hirst, 2011) but was dependent on DQ reduction at the Q-tunnel of CI.

The fact that NADH oxidation is independent of [DQ] indicates that not all DQ is accessible to CI and that at 10 μM DQ, the accessible pool of DQ is already saturated. The accessible DQ pool is likely the DQ that has partitioned into the SC I+III_2 particles because of its hydrophobic nature; hence, the low level of activity seen in the absence of cyt *c* would represent the exchange of SC-associated DQH_2 with bulk solvent DQ. In our conditions, the exchange of SC-associated DQH_2 with bulk solvent DQ occurred at a rate of $\sim 7.0 \pm 0.1\ s^{-1}$ (Figure 3L). If the rate-limiting step of NADH oxidation in the absence of cyt *c* were DQ exchange, then we would expect the addition of BSA or LD buffer to significantly increase the rate. As shown in Figures 2I, 3I, and 3L, this is the case. Overall, these data indicate that in the absence of cyt *c*, CI activity is limited by exchange of "local" SC-associated DQ with the bulk solvent pool. Thus, when cyt *c* is present, the increase in CI activity is directly attributable to the ability of $CIII_2$ to re-oxidize local $CoQH_2$.

CryoEM Structures of Isolated SC I+III_2 Reveal Multiple States

We used the amphipol-stabilized SC I+III_2 to solve the atomic structure of the SC to resolutions up to 3.8 Å (in focused refinements) by cryoEM (Tables 1 and S4), using our improved protocol for model refinement (STAR Methods). CI appeared more stable within the SC than previous preparations of isolated ovine CI (Fiedorczuk et al., 2016), allowing us to improve the model in some peripheral areas that were previously disordered. Initial 3D classification identified four classes of SC I+III_2 particles (Figures 4A–4D; Figure S4C). These differed mainly in the relative angles between the CI peripheral arm and membrane arm and in the angle between CI and $CIII_2$ (Figures 4E and 4F). We concluded that the most distinct class with respect to the position of the CI peripheral arm belonged to the closed state (or active form) of CI (Agip et al., 2018; Fiedorczuk et al., 2016). The assignment of the closed state was based on clear density adjacent to the CI Q-tunnel for the NDUFS2 β1-β2 loop (residues Gly52–Gly60) and for the ND3 TM helix 1 (TMH1)-TMH2 loop (residues Pro25–Lys54; Figure 4G). In all other classes of SC I+III_2, these loops were disordered (Figure 4G), suggesting that they are distinct forms of the open state (or deactive form) of CI (Blaza et al., 2018; Fiedorczuk et al., 2016). Previously, only a single open state had been identified for CI (Agip et al., 2018; Blaza et al., 2018). We identified striking changes in TMH3 of ND6 between the open- and closed-state structures. Whereas in the closed state, ND6 TMH3 was a clear α-helix (residues Leu52–Met74), in each of the open states, the helix was interrupted by a π-bulge midway across the membrane (residues Tyr60–Met65; Figure 4H). The result of this π-to-α transition between the open and closed states of CI is a rotation of ~100° for the C-terminal half of ND6-TM3 (residues Met64–Met74; Figures 4H and 4I).

The three major changes between the closed and open states—the ordering of the NDUFS2 β1-β2 loop in the Q-tunnel, the ordering of the ND3 TMH1-TMH2 loop, and the π-to-α transition of ND6-TM3—occur at the interface of the CI peripheral and membrane arms and were accompanied by a significant rotation between the two arms of the complex, bringing NDUFA5 and NDUFA10 into close contact (Figure 4F). As shown in Figure 4I, the ordered ND3 TMH1-TMH2 loop in the closed state crosses directly above ND6 TMH3. This loop, however, was disordered in the open states. Additional large conformational changes were seen in this area: the short three-stranded β sheet of NDUFS2 that harbors the β1-β2 loop was adjacent to ND6 TMH3 in the closed state but rotated away from TMH3 by as much as 10 Å in the open states (Figure 4I). This movement was accompanied by the striking rotation of the side chains of Phe68 and Tyr70 because of the π-bulge-to-α-helix rearrangement. The analogous structural differences were also observed between the active and deactive forms of CI for mouse CI (Agip et al., 2018), indicating that such large conformational transitions are conserved.

Because of the hinge-like motions between the different regions of the SC, we carried out focused refinements on distinct sub-regions to generate higher quality density maps. These focused refinements were performed on the peripheral arm of CI, on the membrane arm of CI and on $CIII_2$, resulting in

Table 1. CryoEM Map and Model Refinement and Validation Statistics

Reconstruction	SC I+III$_2$ Closed	SC I+III$_2$ Open 1	SC I+III$_2$ Open 2	SC I+III$_2$ Open 3	CI Peripheral Arm	CI Membrane Arm	CIII$_2$	CI Isolated	CI Closed	CI Open 1	CI Open 2	CI Open 3
Number of particles	39,863	35,640	30,836	14,230	178,121	174,334	102,314	57,160	22,107	26,978	25,404	21,913
Accuracy of rotations (°)	0.454	0.398	0.430	0.489	1.37	0.640	1.57	0.523	0.521	0.460	0.474	0.551
Accuracy of translations (pixels)	0.310	0.300	0.300	0.376	0.567	0.418	0.611	0.389	0.381	0.311	0.337	0.390
Box size (pixels)	512	512	512	512	400	512	364	512	512	512	512	512
Final resolution (Å)	4.2	4.2	4.2	4.6	3.8	3.9	3.9	4.1	4.3	4.1	4.2	4.4
MAP SHARPENING B FACTOR (Å^2)	−80	−75	−80	−55	−80	−90	−90	−80	−75	−70	−80	−95
PDB ID	6QBX	6QC3	6QC2	6QC4	6Q9D	6Q9B	6Q9E	6QA9	6QC5	6QC6	6QC8	6QC7
EMDB ID	4493	4495	4494	4496	4480	4479	4481	4482	4497	4498	4500	4499
Refinement												
Software	Phenix 1.14 real-space-refine											
Initial model (PDB code)	5LNK and 1PPJ				5LNK	5LNK	1PPJ	5LNK	5LNK	5LNK	5LNK	5LNK
Map/model correlation												
Model resolution (Å)	4.2	4.2	4.2	4.6	3.9	4.0	4.0	4.1	4.3	4.1	4.2	4.4
d99 (Å)	4.3	4.3	4.3	4.7	4.0	4.1	4.1	4.3	4.4	4.2	4.3	4.4
FSC model 0.5 (Å)	4.4	4.3	4.3	4.7	3.8	4.0	4.0	4.2	4.4	4.2	4.3	4.4
Map CC (masked)	0.71	0.75	0.76	0.75	0.82	0.78	0.80	0.78	0.77	0.77	0.76	0.75
Model composition												
Non-hydrogen atoms	97,049	96,897	96,938	96,705	27,662	38,013	31,997	65,403	65,691	65,344	65,393	65,353
Protein residues	12,092	12,059	12,063	12,071	3,463	4,627	3,988	8,091	8,136	8,085	8,092	8,086
Number of chains	65	65	65	65	18	29	20	45	45	45	45	45
Number of ligands and cofactors	21	21	21	21	12	1	11	13	13	13	13	13
Number of lipids	3	7	9	0	0	12	9	6	3	5	5	2
Atomic displacement parameters (ADP)												
Protein average (Å^2)	56.12	66.11	61.49	103.40	78.1	73.9	82.1	123.88	80.83	73.94	53.37	65.28
Ligand average (Å^2)	67.10	64.66	82.11	144.08	60.9	72.7	95.3	140.18	104.94	83.48	80.59	80.71
Rmsds												
Bond lengths (Å)	0.006	0.010	0.009	0.006	0.006	0.007	0.007	0.007	0.008	0.006	0.008	0.007
Bond angles (°)	1.13	1.29	1.22	1.08	1.08	1.24	1.02	1.19	1.26	1.16	1.24	1.18
Ramachandran plot												
Favored (%)	88.62	88.10	88.45	89.04	89.03	88.62	91.34	87.96	87.51	89.64	88.03	87.62
Allowed (%)	11.26	11.77	11.47	10.82	10.77	11.31	8.63	11.98	12.33	10.29	11.91	12.31
Disallowed (%)	0.13	0.13	0.08	0.13	0.20	0.07	0.03	0.06	0.16	0.08	0.06	0.08

(*Continued on next page*)

CellPress

Table 1. *Continued*

Reconstruction	SC I+III_2 Closed	SC I+III_2 Open 1	SC I+III_2 Open 2	SC I+III_2 Open 3	CI Peripheral Arm	CI Membrane Arm	$CIII_2$	CI Isolated	CI Closed	CI Open 1	CI Open 2	CI Open 3
Validation												
MolProbity score	1.98	2.04	2.00	1.98	1.93	1.92	1.78	2.02	2.05	1.90	2.01	2.00
Clash score	7.10	8.13	7.56	7.29	6.37	6.17	5.17	7.66	7.99	6.22	7.59	7.14
Rotamer outliers (%)	0.40	0.75	0.57	0.38	0.24	0.27	0.42	0.44	0.72	0.55	0.57	0.47
EMRinger score	1.21	1.23	1.26	0.33	2.23	1.64	2.31	1.16	1.10	1.42	1.23	0.85

reconstructions at 3.8–3.9 Å resolution and improved atomic models for each region of the SC (Figure S5; Tables 1 and S5).

Focus-Revert-Classify Strategy Reveals at Least Six Distinct Open States for CI

Although we were able to separate three distinct CI open states using the standard 3D classification, we reasoned that because of the systematic worsening map quality along the peripheral arm of CI (Figure 7; Figure S7), averaging of particles with diverse angles between the membrane and peripheral arms was still occurring. Hence, we developed a strategy for the separation of different CI states on the basis of the angle between the peripheral and membrane arms. Termed "focus-revert-classify," an initial focused refinement around the peripheral arm of CI is performed, maximizing the differences in the relative positions between the membrane arms of each CI, followed by "reverting" to a mask around the membrane arm for classification (Figures 5A and S6A).

The focus-revert-classify strategy resulted in the separation of nine distinct structural classes of CI ranging in resolution from 4.1 to 7.5 Å (Figures 5B and S6A). Of these, seven correspond to distinct open states in which the Q-site loops are disordered, one to a closed state in which the loops around the Q site are well ordered and the final class to a closed-like state at 7.0 Å, precluding the assignment of any of the active site loops. All six CI open state structures at $\leq$6.5 Å each had distinct angles between the peripheral and membrane arms (Figures 5B and 5C). The closed state differs from the open states as described above for the closed state of the SC (Figure 5C). When comparing the particles between the classes of the original 3D classifications of the SC, isolated CI (Figure S4C) and focus-revert-classify (Figure S6A), it is clear that the closed state particles are distinct from all other classes (Figure S6B). The majority of particles that we identified as having CI in the closed state in the original classifications were also found in the closed state class after application of the focus-revert-classify strategy. This was not the case for the three original open state classes, which were more distributed between the six focus-revert-classify open classes. These data indicate that the closed state of CI is a distinct conformation and the open state is an ensemble of nearly continuous conformations differing in the relative positions of CI's membrane and peripheral arms.

Overall Arrangements of the Amphipol-Stabilized SC I+III_2 and CoQ Active Sites

The overall arrangement of the SC I+III_2 shown here is similar to that in the full respirasome (Letts et al., 2016b) as well as *in situ* (Davies et al., 2018). $CIII_2$ contacts CI at two main sites: (1) in the IMM, where CI subunit NDUFA11 contacts UQCRB and UQCRQ of the adjacent CIII protomer (Figure 6A), and (2) in the mitochondrial matrix, where CI subunits NDUFB4 and NDUFB9 (B22) contact UQCRC1 of that same CIII protomer (Figure 6B). At the interface of the mitochondrial inner membrane and matrix, only van der Waals contacts can be seen between the top of NDUFA11 (Leu49) and the main chain of the adjacent UQCRB helix (between Lys11 and Trp12). However, a cluster of positively charged residues extending from both NDUFA11 (Arg103 and Arg105) and UQCRQ (Arg42) suggests the possibility of

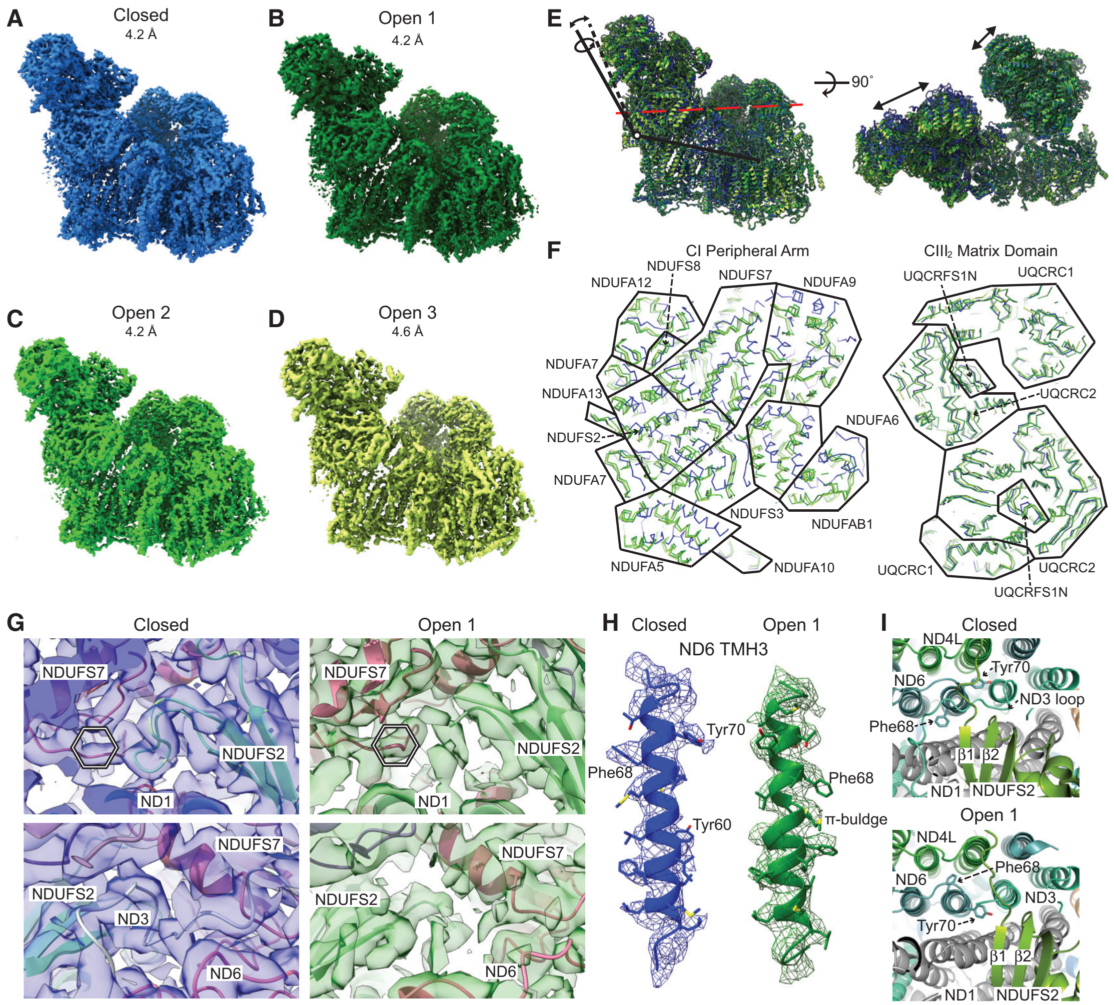

Figure 4. SC I+III$_2$ Structures Reveal State-Dependent Conformational Changes in the CI Membrane Arm

(A–D) CryoEM densities for the (A) closed class, (B) open class 1, (C) open class 2, and (D) open class 3.

(E) Overlay of the models for the different SC classes aligned by the CI membrane arm shown as cartoons. Models colored as in (A)–(D) and viewed from CI side (left) and the mitochondrial matrix (right). Differences in the relative positions of the CI peripheral arm and CIII$_2$ are indicated by arrows.

(F) Slice through the CI peripheral arm and CIII$_2$ at the position indicated by the red dashed line in (E). Models are shown as ribbons and colored as in (E). Approximate boundaries between subunits are indicated by black lines.

(G) CryoEM density for the closed state CI (left, blue density) and open state 1 (right, green density) for the NDUFS2 β1-β2 loop in the Q-tunnel (top) and the ND3 TMH1-TMH2 loop (bottom). Models are shown as cartoons and colored by subunit: green, NDUFS2; red, NDUFS7; light green, ND1; white, ND3; and pink, ND6. The black and white hexagon indicates approximate binding site for CoQ.

(H) ND6 TM3 from the closed state (left, blue) and open state 1 (right, green) viewed from the same side. The π-bulge in the open state 1 is indicated. Models shown as cartoons with side chains as sticks colored by atom, with nitrogen blue, oxygen red, sulfur yellow, and carbon colored as the cartoon helix.

(I) View from the mitochondrial matrix looking at ND6 TMH3 in the closed state (top) and open state 1 (bottom). Models shown as cartoons and colored by subunit: ND4L in green, ND6 in light blue, ND3 in blue-green, ND1 in gray, and NDUFS2 in green. Positions of the ND6 TMH3 side chains Tyr70 and Phe68 are shown. See also Figure S4 and Table S4 for initial processing.

lipid-mediated bridging between CI and CIII$_2$ (Figure 6A). On the inter-membrane space side of the membrane, several charged and polar residues are in close contact (NDUFA11: Glu16, His18, Arg19, and Gln78; UQCRQ: Tyr56, Gln64, and Lys68), which allows the formation of stabilizing hydrogen bonds or salt bridges (Figure 6A). Within the mitochondrial matrix, salt-bridging interactions can be seen between NDUFB9 and NDUFB4 of CI and UQCRC1 of the adjacent CIII protomer (Figure 6B). Possible salt bridges include NDUFB9 Lys54 and UQCRC1 Asp20, NDUFB9 Asp55 and UQCRC1 Arg24, NDUFB4 Glu27 and UQCRC1 Lys51, and NDUFB4 Arg29 and UQCRC1 Glu225 (Figure 6B). Although these interacting residues are conserved in mammals, they diverge quickly in other lineages, likely reflecting different ways of forming SC I+III$_2$ across eukaryotes (Davies et al., 2018).

Given the hydrophobic nature of CoQ-10, it would remain in the fragment of lipid bilayer that is co-purified with the SC I+III$_2$ particles. Hence, even if only limiting amounts of CoQ-10 were

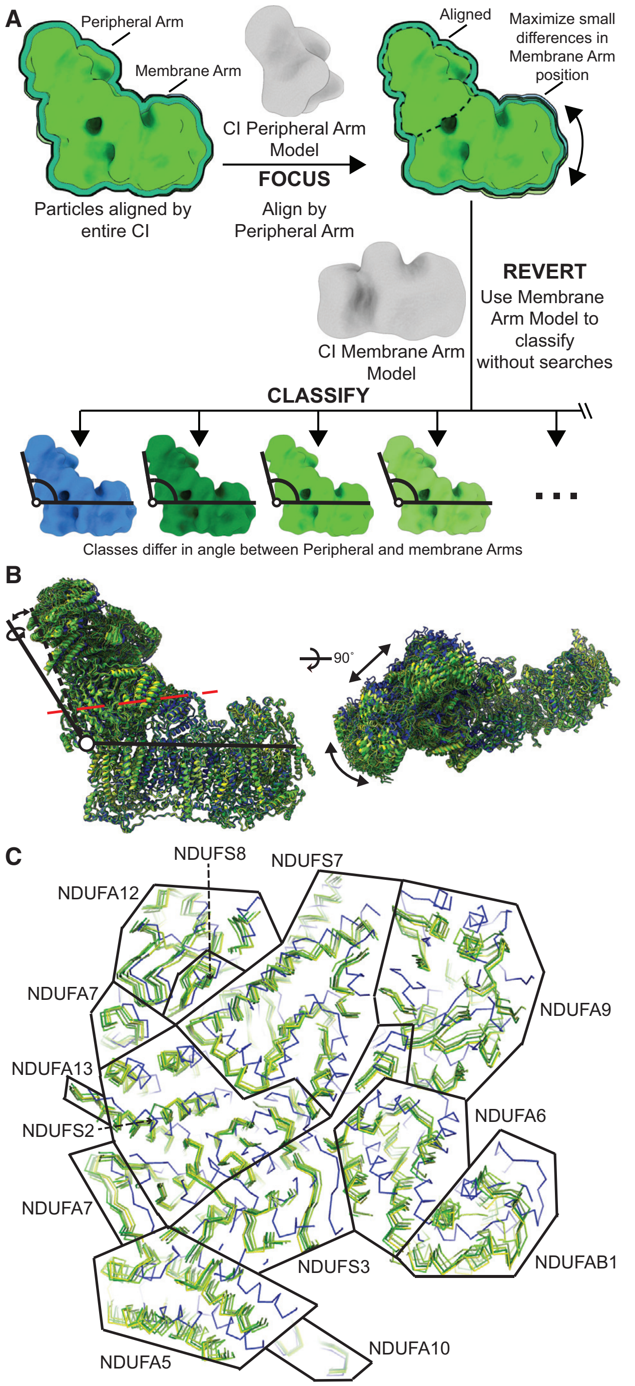

Figure 5. Focus-Revert-Classify Strategy Results in at Least Six Different CI Open Structures

(A) Schematic of focus-revert-classify strategy for separating CI particles on the basis of the angle between the peripheral and membrane arms.

(B) Overlay of CI models generated from the focus-revert-classify strategy aligned by the CI membrane arm. Models colored with the closed state blue and the different open states from dark green to yellow-green according to overall resolution. Viewed from CI side (left) and the mitochondrial matrix (right). Differences in the relative positions of the CI peripheral arm indicated by arrows.

(C) Slice through the CI peripheral arm at the position indicated by red dashed line in (B). Models shown as ribbons and colored as in (B). Approximate boundaries between subunits are indicated by black lines, subunits labeled. See also Figure S6.

co-purified within the SC, its local concentration would be high. Accordingly, although no exogenous CoQ was added to our cryo-EM sample, clear density for the CoQ-10 head group can be seen in the $CIII_2$ focused maps at three of the four CoQ binding sites (Figures 6C–6E). Density could be seen at both Q_N sites, which are expected to have a higher affinity for the oxidized CoQ-10 (Figures 6C and 6D) and the Q_P site distal from the CI Q-tunnel (Figure 6E). However, the Q_P site proximal to the CI Q-tunnel did not display any density for CoQ-10, suggesting different affinities for CoQ-10 in the Q_P sites of the different MT-CYB protomers.

At low contour, the cryoEM maps showed density for a disordered layer of amphipols and lipids forming a belt around the hydrophobic surfaces of the SC (Figure 6F). On the $CIII_2$-proximal side of CI, the four-TMH-containing subunit NDUFA11, which directly interacts with $CIII_2$, is shaped like a lipid-filled arch (Figure 6F), contacting CI with the ends (springers) and $CIII_2$ with the top (crown). The dominance of lipid-mediated contacts between CI and NDUFA11 may explain why this subunit is so easily lost or disordered by detergent when CI is extracted. The rest of the CI-$CIII_2$ interface within the membrane involves two large lipid-filled cavities, made discontinuous by the close interactions among NDUFA11, UQCRB, and UQCRQ. These interactions likely act as a barrier to the free diffusion of $CoQH_2$ as it exits the CI Q-tunnel (Figure 6F).

The two Q cavities of $CIII_2$ penetrate deep into the core of the dimer and are separated from each other by an interaction between the two MT-CYB protomers (Figure 6F). Each Q cavity contains a $CoQH_2$-oxidizing Q_P site and a CoQ-reducing Q_N site; however, the Q_P and Q_N sites within each cavity derive from the opposite CIII protomer (Figure 6F) and, hence, the Q_P and Q_N sites of one MT-CYB subunit face opposite cavities. One of the Q cavities is proximal to the Q-tunnel of CI, while the other is distal on the opposite side of the dimer and much less accessible (Figure 6F). This arrangement led to the hypothesis that symmetry-breaking within $CIII_2$ may result in the specialization of the Q cavities, with the proximal Q cavity specialized for $CoQH_2$ oxidation and the distal cavity specialized for CoQ reduction (Letts and Sazanov, 2017; Letts et al., 2016b). In the amphipol-stabilized SC, trapping of CoQ would limit the diffusion path from the CI Q-tunnel to $CIII_2$ and vice versa to the lipid-amphipol belt surrounding the SC (Figure 6F). Given the overall structure of the SC (Figure 6F) and evidence of poor CoQ exchange in the amphipol-stabilized particle (Figure 3L), functional symmetry-breaking of $CIII_2$ and specialization of the Q-cavities is highly likely.

Local Resolution Analysis Reveals CI State-Dependent Crosstalk

Clear differences can be seen in the local resolution of specific regions of CI and $CIII_2$ between the different reconstructions (Figure 7; Figures S7B and S7C). In the SC I+III_2 class containing

the closed state of CI, the density corresponding to ND4 is of lower resolution compared with the surrounding ND2 and ND5. This lower resolution patch is not seen in the local resolution maps of the open states (Figures 7B and 7D). A major difference between the closed and open states is that contact between the CI peripheral arm and membrane arms via NDUFA5 and NDUFA10 is seen only in the closed state (Figures 4F and 5C). Thus, in the closed state, conformational flexibility in the peripheral arm may be transmitted into the membrane arm. This interaction may also help facilitate coupling of CoQ reduction and H^+ pumping during CI turnover.

Surprisingly, the $CIII_2$ MT-CYB that is in close contact with CI showed lower overall resolution in the SC class containing closed CI than in those with open CI (Figures 7D and 7E). This indicates that this MT-CYB subunit has increased motion or is more disordered in presence of closed CI. The MT-CYB subunit of the CIII protomer in close contact to CI contains the $CoQH_2$ oxidation site most proximal to the Q-tunnel of CI. Hence, this MT-CYB subunit is likely responsible for oxidizing the majority of $CoQH_2$ produced by CI within the SC. The decrease in the local resolution of this subunit only in the closed state of CI strongly suggests that there is crosstalk between the two complexes and that $CIII_2$ may be primed for $CoQH_2$ oxidation upon the closing of CI. Nonetheless, at this resolution it remains unclear how this crosstalk between CI and $CIII_2$ occurs.

DISCUSSION

We present here the first chromatographic isolation of functional SC I+III_2 from mammalian mitochondria, followed by its detailed functional and structural characterization. Previous isolations of this SC from mitochondrial membranes displaying NADH:cyt *c* oxidoreductase activity have been reported (Hatefi and Stiggall, 1978; Hatefi et al., 1961). However, the purity of these preparations is unclear, and their stability is poor. The preparation presented here displays a homogeneous single peak by SEC (Figure 1C) and appears as a single major band

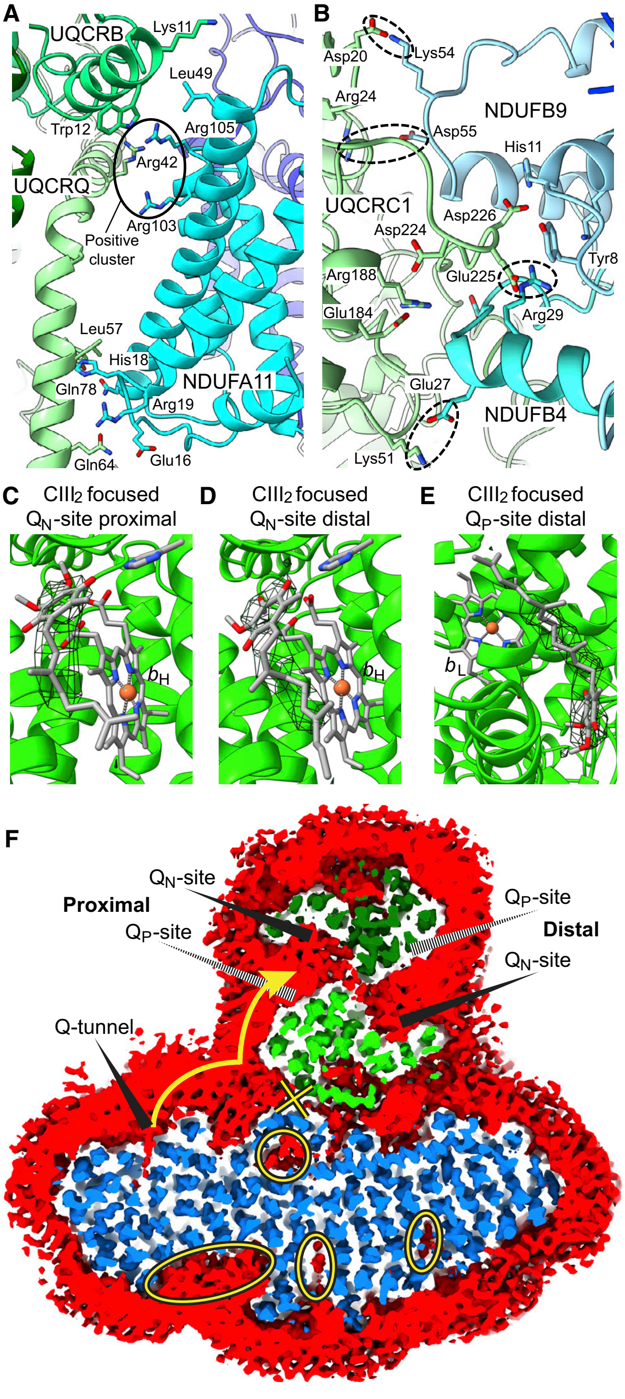

Figure 6. Interactions, CoQ Density, and Overall Arrangement of the Amphipol-Stabilized SC I+III_2 Particles

(A) Interaction between CI and $CIII_2$ in the membrane. CI subunit NDUFA11 in cyan, $CIII_2$ subunits UQCRB in green, and UQCRQ in light green. The putative "positive cluster" lipid-binding site is circled and labeled. Side chains of important residues are shown as sticks and colored by atom, with nitrogen blue, oxygen red, and carbon the same color as the subunit. Models shown as cartoons throughout.

(B) Interaction between CI and $CIII_2$ in the mitochondrial matrix. CI subunits NDUFB4 are in cyan and NDUFB9 in light blue, and $CIII_2$ subunit UQCRC1 are in light green. Important side chains are shown and colored as in (A). Putative salt-bridging interactions indicated by dashed ovals.

(C) Density for CoQ-10 binding in the proximal Q_N-site from the $CIII_2$ focused maps. $CIII_2$ subunits are in green, and CoQ, heme b_H, and the side chain of MT-CYB His201 are shown as sticks and colored by atom, with carbon gray, nitrogen blue, oxygen red, and iron orange.

(D) Density for CoQ-10 binding in the distal Q_N site from the $CIII_2$ focused maps. $CIII_2$ subunits, CoQ, heme b_H, and the MT-CYB side chain His201 are shown as in (C).

(E) Density for CoQ binding in the distal Q_P site from the $CIII_2$ focused maps. $CIII_2$ subunits, CoQ, and heme b_L are shown as in (C).

(F) Slice through the membrane domains of CI and $CIII_2$ within the closed structure of the SC looking from the mitochondrial matrix. Amphipol-lipid belt is shown at low contour in red, and CI (blue) and $CIII_2$ (green) are shown at a higher contour. Each CIII protomer is colored a different shade of green. CoQ active sites are marked: solid wedges for location on the matrix leaflet of the membrane and dashed wedges for inter-membrane space leaflet. Black and yellow ovals indicate lipid binding pockets of CI; black and yellow circle indicates lipid binding pocket of NDUFA11; black and yellow X indicates barrier to CoQ diffusion caused by contact site between CI and $CIII_2$ in the membrane. Yellow arrow illustrates the shortest path for CoQ diffusion from the CI Q-tunnel to the proximal Q_P site of $CIII_2$.

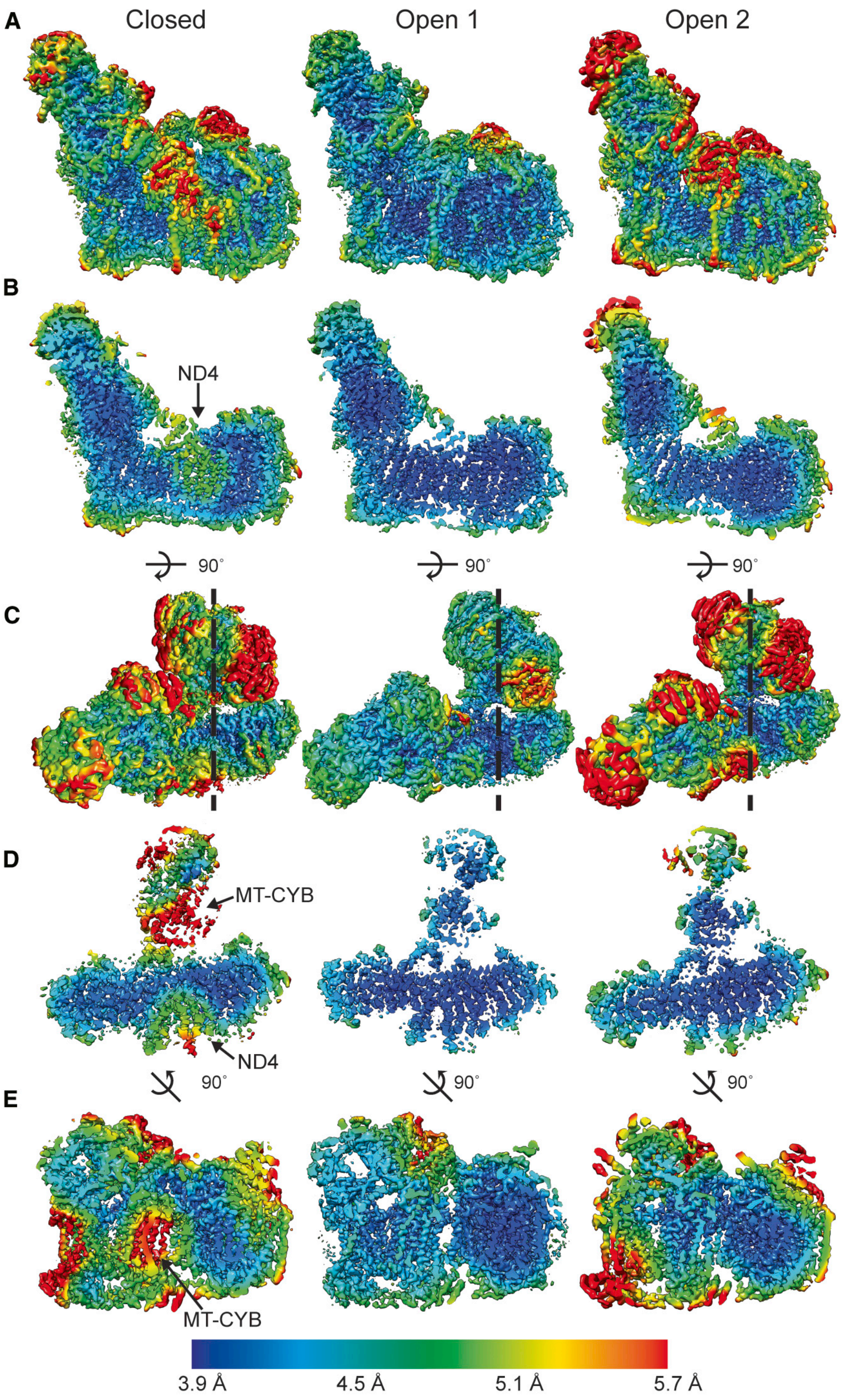

Figure 7. Local Resolution Maps of the SC I+III$_2$ Reconstructions

Local resolution maps of the three 4.2 Å SC I+III$_2$ structures shown with the closed state (left), open state 1 (middle), and open state 2 (right) throughout.

(A) CI side view.

(B) Slice through CI from the same view as in (A).

(C) View from the mitochondrial matrix.

(D) Slice through the membrane domains of CI and CIII$_2$ from the same view as in (C).

(E) Slice through the membrane domains of CI and CIII$_2$ viewed from the "heel" of CI looking at the position indicated by the dashed lines in (C).

See also Figure S7.

on BN-PAGE gels (Figure 1D). The characterization of amphipol-stabilized SC I+III$_2$ NADH:cyt *c* oxidoreductase activity demonstrates that it is possible to study the transfer of electrons from NADH to cyt *c*, both in the absence or presence of added CoQ analogs (Figure 2). The rates of NADH oxidation and cyt *c* reduction observed when the SC was supplemented with 10 μM DQ were lower than what has been observed for CI and CIII$_2$ turnover in membranes (Letts et al., 2016a; Ripple et al., 2013) (Figures 2E and 2F). However, the activity of both complexes increased to native levels upon the addition of lipid and detergent (Figures 2H and 2I). With the purity and inhibitor sensitive activity achieved here (Figures 1, 2, and S3), this preparation can be used for the study of SC I+III$_2$ by diverse biophysical methods.

This preparation is not without its limitations. Because of the lack of a sealed membrane compartment, there is no $\Delta\psi$ or ΔpH present in the system. This is significant, as CI normally operates near equilibrium with the proton motive force (Ripple et al., 2013), and as the redox poise of the *b*-hemes in CIII$_2$ is affected by both $\Delta\psi$ and ΔpH (Kim et al., 2012). Also, because of the nature of the amphipol-based stabilization of the SC in solution, CoQ-10 and to a lesser extent DQ are trapped in the SC particle, and their diffusion is constrained to the lipid-amphipol belt (Figure 6F). CoQ-10 is highly hydrophobic; hence, when extracted into an aqueous solution, CoQ-10 will partition into the hydrophobic environment of the deep lipid pockets present in the SC. Together with the affinities of CI and CIII$_2$ for CoQ-10, a concentrating effect of CoQ-10 within the extracted SC is expected. Hence, it does not follow from CoQ-10 trapping in this context that CoQ-10 is not free to diffuse and exchange with the bulk membrane pool in the hydrophobic environment of the IMM.

Nevertheless, the fact that CoQ is "trapped" by the amphipol-stabilized SC I+III$_2$ was advantageous, as it allowed us to test the hypothesis that CoQ-10 trapping by SC I+III$_2$ *in situ* would increase the rate of the ETC. This system allowed us to determine

how CoQ trapping affects CI and $CIII_2$ activity and to place what we learned into a structural context. Given the arrangement of CI and $CIII_2$ in the SC (Figure 6F), it is likely that only one side of $CIII_2$ would be readily supplied with $CoQH_2$ from CI and that the symmetry of $CIII_2$ would be functionally broken. Nonetheless, given the Hill coefficients of 2 for both the Q_P- and Q_N-site $CIII_2$ inhibitors (Figure S3), it is clear that the CoQ sites in the distal $CIII_2$ Q-cavity also play an important role in turnover. Rate limitation of CI by $CIII_2$ can be understood in the context of the SC I+III_2 structure (Figure 6F). Trapping the CoQ between CI and $CIII_2$ would limit the ability of $CIII_2$ to oxidize $CoQH_2$ to a single Q_P site. In the context of the structure, the ~2-fold increase in $CIII_2$ activity may be due mainly to a re-establishment of two accessible Q_P sites caused by the free exchange of DQH_2 in the LD mixture (Table S3). This indicates that $CIII_2$ within the amphipol-stabilized SC I+III_2 particles is operating at near maximal activity in the experimental context but that only one Q-cavity has access to reduced $CoQH_2$. The rate coupling in these contexts is nearly 2.0 (Table S3), demonstrating that CI activity is limited by $CIII_2$.

Thus, this argues against the view that trapping of CoQ-10 by SCs may increase the flux through the ETC but instead indicates that CI can function more effectively if CoQ is able to exchange with the membrane pool and is not dependent solely on the proximal $CIII_2$ Q_P site for regeneration of oxidized CoQ. The native arrangement within the IMM may allow both CI and $CIII_2$ to operate at the highest rates via exchange with the bulk pool and not via CoQ trapping. Given the structure of the SC, the only way for CoQ trapping to result in higher rates would be if the turnover of CI and the single CIII protomer were well matched. Because of the nature of the Q-cycle, $CIII_2$ must oxidize two $CoQH_2$ for each CoQ reduced by CI. Given the complexity of the Q-cycle mechanism—involving electron bifurcation, conformational transitions of UQCRFS1, and three simultaneously bound substrates—this strict requirement for rate matching may be difficult to accommodate. The mismatch in rates between CI and $CIII_2$ turnover may in part explain why there is an ~3-fold excess of $CIII_2$ compared with CI in mitochondrial membranes (Schägger and Pfeiffer, 2001).

We demonstrate here that the CI closed-to-open state transition involves not only a rotation of the CI peripheral and membrane arms and the ordering of the NDUFS2 β1-β2 and ND3 TMH1-TMH2 loops (Figure 4G) but also a notable rotation of ND6's TMH3 by ~100° that is driven by the conversion of a π-bulge into an α-helix (Figures 4H and 4I). This ND6 TMH3 π-to-α transition was also seen in mouse mitochondrial CI between active and deactive forms (Agip et al., 2018). Although the ovine closed state is likely to be identical to the mouse active form, this is not necessarily the case for the ovine open states and the mouse deactive form. The deactive form of CI is obtained upon incubating the enzyme at 37°C without substrates and then converts back to active upon turnover (Vinogradov, 1998). No significant amount of deactive form was detected biochemically in the ovine enzyme as prepared (data not shown). This is consistent with mouse CI, which is ~90% in the active form as purified (Agip et al., 2018). Given that our preparation was largely in the active state, but many SC particles were in open states, it is likely that CI undergoes conversion between open and closed states while active as part of its catalytic cycle. In this case, the deactive form would represent one of the open-like states with likely more extensive unfolding of loops around the Q-site. A direct link between closed-to-open transitions and the catalytic cycle was also proposed in a recent cryoEM study of *Y. lipolytica* CI (Parey et al., 2018). Given that the ND6 TMH3 π-bulge is conserved in the structures of yeast and bacterial CI (Baradaran et al., 2013; Zickermann et al., 2015), this rotation is likely a conserved feature of CI turnover and may be a novel way of achieving efficient conformational coupling by the enzyme. Although the rotation was not observed in the *Y. lipolytica* or bacterial structures to date, this may be due to limited resolution or crystal contacts.

By careful classification of the SC particles, we show that there are state-dependent differences in local resolution (Figure 7). In the closed state of CI, conformational flexibility in the peripheral arm may be transmitted to ND4 in the membrane arm via interaction between NDUFA5 and NDUFA10 (Figure 7), possibly facilitating coupling of CoQ reduction and H^+ pumping during CI turnover. How this state-dependent conformational flexibility is further communicated to $CIII_2$ remains unclear. However, our structures of multiple states of CI within the SC at similar resolutions suggest that the state of CI affects $CIII_2$'s MT-CYB conformational flexibility (Figures 7D and 7E). The fact that CoQ was found at only three of the four possible binding sites within $CIII_2$ (Figures 6C–6E) also suggests a functionally relevant effect of CI on $CIII_2$. Overall, our data further support the hypothesis of functional symmetry breaking in the $CIII_2$ dimer by CI.

Our pure, biochemically defined system allowed us to demonstrate that in contrast to the long-proposed substrate-channeling role for the SC, CoQ trapping in the SC limits CI turnover and would reduce the overall rate of the ETC *in vivo*. If CoQ trapping is inefficient, what could be the functional role of the SCs? Possible roles in the stabilization of the individual complexes, the reduction of ROS production, and the prevention of non-specific protein aggregation such that the IMM retains suitable CI/$CIII_2$/CIV ratios remain feasible. The SCs may provide kinetic advantages by bringing the active sites into close proximity, but this advantage would exist even in the absence of CoQ trapping by the SCs, as it is only dependent on the distance between the active sites. Moreover, under high turnover, because of the structure of the mitochondrial membranes, individual CIs may outpace the capacity for CoQ pool equilibration, in which case the local $CoQH_2$ concentration may become higher than in the bulk membrane (Budin et al., 2018). By ensuring close association of CI and $CIII_2$ in SCs, but still allowing free exchange of CoQ with the bulk, the SC would prevent any local buildup of $CoQH_2$, while at the same time not limiting CI's activity by making it entirely dependent on the adjacent $CIII_2$ for the re-oxidation of $CoQH_2$. We show here, for the first time, functional crosstalk between CI and $CIII_2$ within the SC. Although more work is needed to confirm and characterize the nature of this coordination, our results and our new experimental framework have significant implications for the physiological roles of respiratory SCs, suggesting more subtle functionally relevant interactions between CI and $CIII_2$.

STAR★METHODS

Detailed methods are provided in the online version of this paper and include the following:

- KEY RESOURCES TABLE
- LEAD CONTACT AND MATERIALS AVAILABILITY
- EXPERIMENTAL MODEL AND SUBJECT DETAILS
- METHOD DETAILS
  - Purification of amphipol-stabilized SC I+III_2
  - Blue Native PAGE
  - Mass Spectrometry
  - Organic Phosphate Assay
  - Activity Assays
  - CryoEM grid preparation, optimization, and data acquisition
  - Image processing and 3D reconstruction
  - Atomic model building and refinement
- QUANTIFICATION AND STATISTICAL ANALYSIS
- DATA AND CODE AVAILABILITY
  - Data Resources

SUPPLEMENTAL INFORMATION

Supplemental Information can be found online at https://doi.org/10.1016/j.molcel.2019.07.022.

ACKNOWLEDGMENTS

We thank DIAMOND UK (eBIC) for the use of the Titan Krios. Data processing was done with the IST computer cluster. This work was supported in part by the European Union (EU) 2020 Research and Innovation Program (grant 701309).

AUTHOR CONTRIBUTIONS

Conceptualization, J.A.L. and L.A.S.; Methodology, J.A.L. and L.A.S.; Validation, J.A.L.; Formal Analysis, J.A.L. and G.D.; Investigation, J.A.L., K.F., and G.D.; Data Curation, J.A.L.; Writing – Original Draft, J.A.L.; Writing – Review & Editing, J.A.L. and L.A.S.; Visualization, J.A.L.; Supervision, M.S. and L.A.S.; Project Administration, J.A.L., M.S., and L.A.S.; Funding Acquisition, J.A.L., M.S., and L.A.S.

DECLARATION OF INTERESTS

The authors declare no competing interests.

Received: November 22, 2018
Revised: June 10, 2019
Accepted: July 15, 2019
Published: September 3, 2019

REFERENCES

Adams, P.D., Afonine, P.V., Bunkóczi, G., Chen, V.B., Davis, I.W., Echols, N., Headd, J.J., Hung, L.W., Kapral, G.J., Grosse-Kunstleve, R.W., et al. (2010). PHENIX: a comprehensive Python-based system for macromolecular structure solution. Acta Crystallogr. D Biol. Crystallogr. *66*, 213–221.

Afonine, P.V., Grosse-Kunstleve, R.W., and Adams, P.D. (2005). The Phenix refinement framework. http://www.phenix-online.org/papers/ccp4_july_2005_afonine.pdf.

Agip, A.A., Blaza, J.N., Bridges, H.R., Viscomi, C., Rawson, S., Muench, S.P., and Hirst, J. (2018). Cryo-EM structures of complex I from mouse heart mitochondria in two biochemically defined states. Nat. Struct. Mol. Biol. *25*, 548–556.

Acín-Pérez, R., Bayona-Bafaluy, M.P., Fernández-Silva, P., Moreno-Loshuertos, R., Pérez-Martos, A., Bruno, C., Moraes, C.T., and Enríquez, J.A. (2004). Respiratory complex III is required to maintain complex I in mammalian mitochondria. Mol. Cell *13*, 805–815.

Anderson, R.L., and Davis, S. (1982). An organic phosphorus assay which avoids the use of hazardous perchloric acid. Clin. Chim. Acta *121*, 111–116.

Baker, L.A., and Rubinstein, J.L. (2010). Radiation damage in electron cryomicroscopy. Methods Enzymol. *481*, 371–388.

Baradaran, R., Berrisford, J.M., Minhas, G.S., and Sazanov, L.A. (2013). Crystal structure of the entire respiratory complex I. Nature *494*, 443–448.

Bianchi, C., Genova, M.L., Parenti Castelli, G., and Lenaz, G. (2004). The mitochondrial respiratory chain is partially organized in a supercomplex assembly: kinetic evidence using flux control analysis. J. Biol. Chem. *279*, 36562–36569.

Blaza, J.N., Serreli, R., Jones, A.J.Y., Mohammed, K., and Hirst, J. (2014). Kinetic evidence against partitioning of the ubiquinone pool and the catalytic relevance of respiratory-chain supercomplexes. Proc. Natl. Acad. Sci. USA *111*, 15735–15740.

Blaza, J.N., Vinothkumar, K.R., and Hirst, J. (2018). Structure of the deactive state of mammalian respiratory complex I. Structure *26*, 312–319.e3.

Brandt, U., and Okun, J.G. (1997). Role of deprotonation events in ubihydroquinone:cytochrome c oxidoreductase from bovine heart and yeast mitochondria. Biochemistry *36*, 11234–11240.

Budin, I., de Rond, T., Chen, Y., Chan, L.J.G., Petzold, C.J., and Keasling, J.D. (2018). Viscous control of cellular respiration by membrane lipid composition. Science *362*, 1186–1189.

Calvaruso, M.A., Willems, P., van den Brand, M., Valsecchi, F., Kruse, S., Palmiter, R., Smeitink, J., and Nijtmans, L. (2012). Mitochondrial complex III stabilizes complex I in the absence of NDUFS4 to provide partial activity. Hum. Mol. Genet. *21*, 115–120.

Chazotte, B., and Hackenbrock, C.R. (1988). The multicollisional, obstructed, long-range diffusional nature of mitochondrial electron transport. J. Biol. Chem. *263*, 14359–14367.

Chen, V.B., Arendall, W.B., 3rd, Headd, J.J., Keedy, D.A., Immormino, R.M., Kapral, G.J., Murray, L.W., Richardson, J.S., and Richardson, D.C. (2010). MolProbity: all-atom structure validation for macromolecular crystallography. Acta Crystallogr. D Biol. Crystallogr. *66*, 12–21.

Chen, S., McMullan, G., Faruqi, A.R., Murshudov, G.N., Short, J.M., Scheres, S.H.W., and Henderson, R. (2013). High-resolution noise substitution to measure overfitting and validate resolution in 3D structure determination by single particle electron cryomicroscopy. Ultramicroscopy *135*, 24–35.

Covian, R., and Trumpower, B.L. (2008). Regulatory interactions in the dimeric cytochrome bc(1) complex: the advantages of being a twin. Biochim. Biophys. Acta *1777*, 1079–1091.

Cramer, W.A., Hasan, S.S., and Yamashita, E. (2011). The Q cycle of cytochrome bc complexes: a structure perspective. Biochim. Biophys. Acta *1807*, 788–802.

D’Imprima, E., Salzer, R., Bhaskara, R.M., Sánchez, R., Rose, I., Kirchner, L., Hummer, G., Kühlbrandt, W., Vonck, J., and Averhoff, B. (2017). Cryo-EM structure of the bifunctional secretin complex of *Thermus thermophilus*. eLife *6*, 403.

Davies, K.M., Blum, T.B., and Kühlbrandt, W. (2018). Conserved in situ arrangement of complex I and III_2 in mitochondrial respiratory chain supercomplexes of mammals, yeast, and plants. Proc. Natl. Acad. Sci. USA *115*, 3024–3029.

Emsley, P., Lohkamp, B., Scott, W.G., and Cowtan, K. (2010). Features and development of Coot. Acta Crystallogr. D Biol. Crystallogr. *66*, 486–501.

Enríquez, J.A. (2016). Supramolecular organization of respiratory complexes. Annu. Rev. Physiol. *78*, 533–561.

Estornell, E., Fato, R., Pallotti, F., and Lenaz, G. (1993). Assay conditions for the mitochondrial NADH:coenzyme Q oxidoreductase. FEBS Lett. *332*, 127–131.

Fedor, J.G., and Hirst, J. (2018). Mitochondrial supercomplexes do not enhance catalysis by quinone channeling. Cell Metab. *28*, 525–531.e4.

Fiedorczuk, K., Letts, J.A., Degliesposti, G., Kaszuba, K., Skehel, M., and Sazanov, L.A. (2016). Atomic structure of the entire mammalian mitochondrial complex I. Nature *538*, 406–410.

Formosa, L.E., Dibley, M.G., Stroud, D.A., and Ryan, M.T. (2018). Building a complex complex: Assembly of mitochondrial respiratory chain complex I. Semin. Cell Dev. Biol. *76*, 154–162.

Glaeser, R.M., and Han, B.G. (2017). Opinion: hazards faced by macromolecules when confined to thin aqueous films. Biophys. Rep. *3*, 1–7.

Glaeser, R.M., Han, B.G., Csencsits, R., Killilea, A., Pulk, A., and Cate, J.H.D. (2016). Factors that influence the formation and stability of thin, Cryo-EM specimens. Biophys. J. *110*, 749–755.

Goddard, T.D., Huang, C.C., Meng, E.C., Pettersen, E.F., Couch, G.S., Morris, J.H., and Ferrin, T.E. (2018). UCSF ChimeraX: meeting modern challenges in visualization and analysis. Protein Sci. *27*, 14–25.

Gu, J., Wu, M., Guo, R., Yan, K., Lei, J., Gao, N., and Yang, M. (2016). The architecture of the mammalian respirasome. Nature *537*, 639–643.

Guarás, A., Perales-Clemente, E., Calvo, E., Acín-Pérez, R., Loureiro-Lopez, M., Pujol, C., Martínez-Carrascoso, I., Nuñez, E., García-Marqués, F., Rodríguez-Hernández, M.A., et al. (2016). The CoQH2/CoQ ratio serves as a sensor of respiratory chain efficiency. Cell Rep. *15*, 197–209.

Guerrero-Castillo, S., Baertling, F., Kownatzki, D., Wessels, H.J., Arnold, S., Brandt, U., and Nijtmans, L. (2017). The assembly pathway of mitochondrial respiratory chain complex I. Cell Metab. *25*, 128–139.

Guo, R., Zong, S., Wu, M., Gu, J., and Yang, M. (2017). Architecture of human mitochondrial respiratory megacomplex $I_2III_2IV_2$. Cell *170*, 1247–1257.e12.

Gupte, S., Wu, E.S., Hoechli, L., Hoechli, M., Jacobson, K., Sowers, A.E., and Hackenbrock, C.R. (1984). Relationship between lateral diffusion, collision frequency, and electron transfer of mitochondrial inner membrane oxidation-reduction components. Proc. Natl. Acad. Sci. USA *81*, 2606–2610.

Gutierrez-Cirlos, E.B., and Trumpower, B.L. (2002). Inhibitory analogs of ubiquinol act anti-cooperatively on the Yeast cytochrome bc1 complex. Evidence for an alternating, half-of-the-sites mechanism of ubiquinol oxidation. J. Biol. Chem. *277*, 1195–1202.

Hackenbrock, C.R., Chazotte, B., and Gupte, S.S. (1986). The random collision model and a critical assessment of diffusion and collision in mitochondrial electron transport. J. Bioenerg. Biomembr. *18*, 331–368.

Hatefi, Y., and Stiggall, D.L. (1978). Preparation and properties of NADH: cytochrome *c* oxidoreductase (complex I–III). Methods Enzymol. *53*, 5–10.

Hatefi, Y., Jurtshuk, P., and Haavik, A.G. (1961). Studies on the electron transport system. XXXI. DPNH-cytochrome c reductase II. Biochim. Biophys. Acta *52*, 119–129.

Hatefi, Y., Haavik, A.G., Fowler, L.R., and Griffiths, D.E. (1962). Studies on the electron transfer system. XLII. Reconstitution of the electron transfer system. J. Biol. Chem. *237*, 2661–2669.

Hirst, J. (2013). Mitochondrial complex I. Annu. Rev. Biochem. *82*, 551–575.

Horvath, S.E., and Daum, G. (2013). Lipids of mitochondria. Prog. Lipid Res. *52*, 590–614.

Huang, L.S., Cobessi, D., Tung, E.Y., and Berry, E.A. (2005). Binding of the respiratory chain inhibitor antimycin to the mitochondrial bc1 complex: a new crystal structure reveals an altered intramolecular hydrogen-bonding pattern. J. Mol. Biol. *351*, 573–597.

Hunte, C., Koepke, J., Lange, C., Rossmanith, T., and Michel, H. (2000). Structure at 2.3 A resolution of the cytochrome bc(1) complex from the yeast Saccharomyces cerevisiae co-crystallized with an antibody Fv fragment. Structure *8*, 669–684.

Iwata, S., Ostermeier, C., Ludwig, B., and Michel, H. (1995). Structure at 2.8 A resolution of cytochrome c oxidase from Paracoccus denitrificans. Nature *376*, 660–669.

Iwata, S., Lee, J.W., Okada, K., Lee, J.K., Iwata, M., Rasmussen, B., Link, T.A., Ramaswamy, S., and Jap, B.K. (1998). Complete structure of the 11-subunit bovine mitochondrial cytochrome bc_1 complex. Science *281*, 64–71.

Keller, A., Nesvizhskii, A.I., Kolker, E., and Aebersold, R. (2002). Empirical statistical model to estimate the accuracy of peptide identifications made by MS/MS and database search. Anal. Chem. *74*, 5383–5392.

Kim, N., Ripple, M.O., and Springett, R. (2012). Measurement of the mitochondrial membrane potential and pH gradient from the redox poise of the hemes of the bc1 complex. Biophys. J. *102*, 1194–1203.

Kimanius, D., Forsberg, B.O., Scheres, S.H.W., and Lindahl, E. (2016). Accelerated cryo-EM structure determination with parallelisation using GPUs in RELION-2. eLife *5*, e18722.

Kröger, A., and Klingenberg, M. (1973a). The kinetics of the redox reactions of ubiquinone related to the electron-transport activity in the respiratory chain. Eur. J. Biochem. *34*, 358–368.

Kröger, A., and Klingenberg, M. (1973b). Further evidence for the pool function of ubiquinone as derived from the inhibition of the electron transport by antimycin. Eur. J. Biochem. *39*, 313–323.

Lapuente-Brun, E., Moreno-Loshuertos, R., Acín-Pérez, R., Latorre-Pellicer, A., Colás, C., Balsa, E., Perales-Clemente, E., Quirós, P.M., Calvo, E., Rodríguez-Hernández, M.A., et al. (2013). Supercomplex assembly determines electron flux in the mitochondrial electron transport chain. Science *340*, 1567–1570.

Lenaz, G., Tioli, G., Falasca, A.I., and Genova, M.L. (2016). Complex I function in mitochondrial supercomplexes. Biochim. Biophys. Acta *1857*, 991–1000.

Letts, J.A., and Sazanov, L.A. (2015). Gaining mass: the structure of respiratory complex I-from bacterial towards mitochondrial versions. Curr. Opin. Struct. Biol. *33*, 135–145.

Letts, J.A., and Sazanov, L.A. (2017). Clarifying the supercomplex: the higher-order organization of the mitochondrial electron transport chain. Nat. Struct. Mol. Biol. *24*, 800–808.

Letts, J.A., Degliesposti, G., Fiedorczuk, K., Skehel, M., and Sazanov, L.A. (2016a). Purification of ovine respiratory complex I results in a highly active and stable preparation. J. Biol. Chem. *291*, 24657–24675.

Letts, J.A., Fiedorczuk, K., and Sazanov, L.A. (2016b). The architecture of respiratory supercomplexes. Nature *537*, 644–648.

Lopez-Fabuel, I., Le Douce, J., Logan, A., James, A.M., Bonvento, G., Murphy, M.P., Almeida, A., and Bolaños, J.P. (2016). Complex I assembly into supercomplexes determines differential mitochondrial ROS production in neurons and astrocytes. Proc. Natl. Acad. Sci. USA *113*, 13063–13068.

Merritt, E.A. (2012). To B or not to B: a question of resolution? Acta Crystallogr. D Biol. Crystallogr. *68*, 468–477.

Milenkovic, D., Blaza, J.N., Larsson, N.G., and Hirst, J. (2017). The enigma of the respiratory chain supercomplex. Cell Metab. *25*, 765–776.

Mileykovskaya, E., and Dowhan, W. (2014). Cardiolipin-dependent formation of mitochondrial respiratory supercomplexes. Chem. Phys. Lipids *179*, 42–48.

Nett, J.H., Hunte, C., and Trumpower, B.L. (2000). Changes to the length of the flexible linker region of the Rieske protein impair the interaction of ubiquinol with the cytochrome bc1 complex. Eur. J. Biochem. *267*, 5777–5782.

Parey, K., Brandt, U., Xie, H., Mills, D.J., Siegmund, K., Vonck, J., Kühlbrandt, W., and Zickermann, V. (2018). Cryo-EM structure of respiratory complex I at work. eLife *7*, 213.

Perkins, D.N., Pappin, D.J., Creasy, D.M., and Cottrell, J.S. (1999). Probability-based protein identification by searching sequence databases using mass spectrometry data. Electrophoresis *20*, 3551–3567.

Persson, E.M., Gustafsson, A.S., Carlsson, A.S., Nilsson, R.G., Knutson, L., Forsell, P., Hanisch, G., Lennernäs, H., and Abrahamsson, B. (2005). The effects of food on the dissolution of poorly soluble drugs in human and in model small intestinal fluids. Pharm. Res. *22*, 2141–2151.

Pettersen, E.F., Goddard, T.D., Huang, C.C., Couch, G.S., Greenblatt, D.M., Meng, E.C., and Ferrin, T.E. (2004). UCSF Chimera—a visualization system for exploratory research and analysis. J. Comput. Chem. *25*, 1605–1612.

Pryde, K.R., and Hirst, J. (2011). Superoxide is produced by the reduced flavin in mitochondrial complex I: a single, unified mechanism that applies during both forward and reverse electron transfer. J. Biol. Chem. *286*, 18056–18065.

Ripple, M.O., Kim, N., and Springett, R. (2013). Mammalian complex I pumps 4 protons per 2 electrons at high and physiological proton motive force in living cells. J. Biol. Chem. *288*, 5374–5380.

Rosenthal, P.B., and Henderson, R. (2003). Optimal determination of particle orientation, absolute hand, and contrast loss in single-particle electron cryomicroscopy. J. Mol. Biol. *333*, 721–745.

Sazanov, L.A. (2015). A giant molecular proton pump: structure and mechanism of respiratory complex I. Nat. Rev. Mol. Cell Biol. *16*, 375–388.

Schägger, H., and Pfeiffer, K. (2000). Supercomplexes in the respiratory chains of yeast and mammalian mitochondria. EMBO J. *19*, 1777–1783.

Schägger, H., and Pfeiffer, K. (2001). The ratio of oxidative phosphorylation complexes I-V in bovine heart mitochondria and the composition of respiratory chain supercomplexes. J. Biol. Chem. *276*, 37861–37867.

Schägger, H., Hagen, T., Roth, B., Brandt, U., Link, T.A., and von Jagow, G. (1990). Phospholipid specificity of bovine heart bc1 complex. Eur. J. Biochem. *190*, 123–130.

Schägger, H., de Coo, R., Bauer, M.F., Hofmann, S., Godinot, C., and Brandt, U. (2004). Significance of respirasomes for the assembly/stability of human respiratory chain complex I. J. Biol. Chem. *279*, 36349–36353.

Sharpley, M.S., Shannon, R.J., Draghi, F., and Hirst, J. (2006). Interactions between phospholipids and NADH:ubiquinone oxidoreductase (complex I) from bovine mitochondria. Biochemistry *45*, 241–248.

Shinzawa-Itoh, K., Seiyama, J., Terada, H., Nakatsubo, R., Naoki, K., Nakashima, Y., and Yoshikawa, S. (2010). Bovine heart NADH-ubiquinone oxidoreductase contains one molecule of ubiquinone with ten isoprene units as one of the cofactors. Biochemistry *49*, 487–492.

Smith, P.M., Fox, J.L., and Winge, D.R. (2012). Biogenesis of the cytochrome bc1 complex and role of assembly factors. Biochim. Biophys. Acta *1817*, 276–286.

Sousa, J.S., Mills, D.J., Vonck, J., and Kühlbrandt, W. (2016). Functional asymmetry and electron flow in the bovine respirasome. eLife *5*, 805.

Subrahmanian, N., Remacle, C., and Hamel, P.P. (2016). Plant mitochondrial Complex I composition and assembly: a review. Biochim. Biophys. Acta *1857*, 1001–1014.

Sun, F., Huo, X., Zhai, Y., Wang, A., Xu, J., Su, D., Bartlam, M., and Rao, Z. (2005). Crystal structure of mitochondrial respiratory membrane protein complex II. Cell *121*, 1043–1057.

Tsukihara, T., Aoyama, H., Yamashita, E., Tomizaki, T., Yamaguchi, H., Shinzawa-Itoh, K., Nakashima, R., Yaono, R., and Yoshikawa, S. (1996). The whole structure of the 13-subunit oxidized cytochrome *c* oxidase at 2.8 A. Science *272*, 1136–1144.

Vinogradov, A.D. (1998). Catalytic properties of the mitochondrial NADH-ubiquinone oxidoreductase (complex I) and the pseudo-reversible active/inactive enzyme transition. Biochim. Biophys. Acta *1364*, 169–185.

Wu, M., Gu, J., Guo, R., Huang, Y., and Yang, M. (2016). Structure of mammalian respiratory supercomplex $I_1III_2IV_1$. Cell *167*, 1598–1609.e10.

Zhang, K. (2016). Gctf: real-time CTF determination and correction. J. Struct. Biol. *193*, 1–12.

Zheng, S.Q., Palovcak, E., Armache, J.P., Verba, K.A., Cheng, Y., and Agard, D.A. (2017). MotionCor2: anisotropic correction of beam-induced motion for improved cryo-electron microscopy. Nat. Methods *14*, 331–332.

Zhu, J., Vinothkumar, K.R., and Hirst, J. (2016). Structure of mammalian respiratory complex I. Nature *536*, 354–358.

Zickermann, V., Wirth, C., Nasiri, H., Siegmund, K., Schwalbe, H., Hunte, C., and Brandt, U. (2015). Structural biology. Mechanistic insight from the crystal structure of mitochondrial complex I. Science *347*, 44–49.

STAR★METHODS

KEY RESOURCES TABLE

REAGENT or RESOURCE	SOURCE	IDENTIFIER
Biological Samples		
Ovine hearts	C Humphrey's & Sons (Chelmsford, UK)	N/A
Chemicals, Peptides, and Recombinant Proteins		
NADH	Sigma-Aldrich	Cat# 1246440005; CAS 606-68-8
Decylubiquinone	Santa Cruz Biotechnology	Cat# 358659; CAS 55486-00-5
Cytochrome *c* from bovine heart	Sigma-Aldrich	Cat# 30398; CAS 9007-43-6
BSA	Cell signaling technologies	Cat# 9998S
LMNG	Anatrace	Cat# NG310
Amphipol A8-35	Anatrace	Cat# A835
DOPC	Sigma-Aldrich	Cat# P6354; CAS 4235-95-4
Cardiolipin	Sigma-Aldrich	Cat# 21979; CAS 200-578-6
Critical Commercial Assays		
NativePAGE 3-12% Bis-Tris Gel	Invitrogen	Cat# BN1003
NativePAGE Cathode Buffer Additive (20X)	Invitrogen	Cat# BN2002
NOVEX 4-20% Tris-Glycine Gel	Invitrogen	Cat# XP04205
Pierce BCA Protein Assay Kit	Thermo Fisher Scientific	Cat# 23227
MonoQ 5/50 GL	GE Healthcare	Cat# 17-5166-01
Q-Sepharose Fast Flow	GE Healthcare	Cat# 17-0510-01
Superose 6 prep grade, XK 6/100	GE Healthcare	Cat# 90-1003-36
Deposited Data		
Ovine CI model	Fiedorczuk et al., 2016	PDB: 4HEA
Bovine $CIII_2$ model	Huang et al., 2005	PDB: 1PPJ
Coordinates of ovine CI Peripheral arm	This study	PDB: 6Q9D
CryoEM map of ovine CI Peripheral Arm	This study	EMDB: 4480
Coordinates of ovine CI Membrane Arm	This study	PDB: 6Q9B
CryoEM map of ovine CI Membrane Arm	This study	EMDB: 4479
Coordinates of ovine $CIII_2$	This study	PDB: 6QE9
CryoEM map of ovine $CIII_2$	This study	EMDB: 4481
Coordinates of ovine SC I+III_2 closed class	This study	PDB: 6QBX
CryoEM map of ovine SC I+III_2 closed class	This study	EMDB: 4493
Coordinates of ovine SC I+III_2 open class 1	This study	PDB: 6QC3
CryoEM map of ovine SC I+III_2 open class 1	This study	EMDB: 4495
Coordinates of ovine SC I+III_2 open class 2	This study	PDB: 6QC2
CryoEM map of ovine SC I+III_2 open class 2	This study	EMDB: 4494
Coordinates of ovine SC I+III_2 open class 3	This study	PDB: 6QC4
CryoEM map of ovine SC I+III_2 open class 3	This study	EMDB: 4496
Coordinates of ovine isolated CI class	This study	PDB: 6QA9
CryoEM map of ovine isolated CI class	This study	EMDB: 4482
Coordinates of ovine CI FRC closed class	This study	PDB: 6QC5
CryoEM map of ovine CI FRC closed class	This study	EMDB: 4497
Coordinates of ovine CI FRC open class 1	This study	PDB: 6QC6
CryoEM map of ovine CI FRC open class 1	This study	EMDB: 4498
Coordinates of ovine CI FRC open class 2	This study	PDB: 6QC8

(*Continued on next page*)

Continued		
REAGENT or RESOURCE	SOURCE	IDENTIFIER
CryoEM map of ovine CI FRC open class 2	This study	EMDB: 4500
Coordinates of ovine CI FRC open class 3	This study	PDB: 6QC7
CryoEM map of ovine CI FRC open class 3	This study	EMDB: 4499
Coordinates of ovine CI FRC open class 4	This study	PDB: 6QC9
CryoEM map of ovine CI FRC open class 4	This study	EMDB: 4501
Coordinates of ovine CI FRC open class 5	This study	PDB: 6QCA
CryoEM map of ovine CI FRC open class 5	This study	EMDB: 4502
Coordinates of ovine CI FRC open class 6	This study	PDB: 6QCF
CryoEM map of ovine CI FRC open class 6	This study	EMDB: 4505
CryoEM map of ovine CI FRC poor closed class	This study	EMDB: 4506
CryoEM map of ovine CI FRC poor open class	This study	EMDB: 4507
Software and Algorithms		
FEI EPU	FEI	https://www.fei.com/software/epu/
MotionCor2	Zheng et al., 2017	http://msg.ucsf.edu/em/software/motioncor2.html
Gctf	Zhang, 2016	https://www.mrc-lmb.cam.ac.uk/kzhang/Gctf/
RELION-2.0	Kimanius et al., 2016	https://www2.mrc-lmb.cam.ac.uk/relion
UCSF Chimera	Pettersen et al., 2004	https://www.cgl.ucsf.edu/chimera/
UCSF ChimeraX	Goddard et al., 2018	http://www.rbvi.ucsf.edu/chimerax/
Coot	Emsley et al., 2010	https://www2.mrc-lmb.cam.ac.uk/personal/pemsley/coot/
PHENIX	Adams et al., 2010	https://www.phenix-online.org/
MolProbity	Chen et al., 2010	http://molprobity.biochem.duke.edu/
PyMol	Schrödinger, LLC.	https://pymol.org/2/
PRISM 5	GraphPad Software	https://www.graphpad.com/scientific-software/prism/
REP	D'Imprima et al., 2017	https://github.com/rkms86/REP
Other		
EM grid R 0.6/1 on 300 mesh Cu	Quantifoil	Item# N1-C11nCu30-01

LEAD CONTACT AND MATERIALS AVAILABILITY

Further information and requests for resources and reagents should be directed to and will be fulfilled by the Lead Contact, Leonid A. Sazanov (sazanov@ist.ac.at)

EXPERIMENTAL MODEL AND SUBJECT DETAILS

Ovis aries (sheep) hearts were purchased from C Humphreys & Sons (Chelmsford, UK).

METHOD DETAILS

Purification of amphipol-stabilized SC I+III$_2$

Mitochondria were isolated and membranes prepared as previously described (Letts et al., 2016a). Complex (C)I purification in LMNG was initially performed as previously reported (Letts et al., 2016a), consisting of a single anion-exchange step followed by SEC. In short, 5% (w/v) LMNG was added drop-wise to the washed mitochondrial membranes to a final concentration of 1% LMNG, followed by stirring for ~30 min at 4°C and centrifugation at 48,000 *g* for 45 min. The supernatant was filtered (0.45 μm pore size polyethersulfone membrane) and loaded onto a pre-equilibrated 45 mL Q-Sepharose FF anion-exchange column (GE Healthcare). The Q-Sepharose buffers (A and B) contained 20 mM Tris-HCl, pH 7.4, 10% (v/v) glycerol, 1 mM EDTA, 1 mM DTT and 0.04% LMNG; additionally, buffer B contained 1 M NaCl; the Q-Sepharose column was pre-equilibrated at 5% buffer B in buffer A. After application of the mitochondrial extract, the Q-Sepharose column was washed with 50 mL of 5% buffer B, then with a 30-mL linear gradient of 5%–22%, and finally with 150 mL of 22% buffer B. CI was then eluted with a 300-mL linear gradient

of 22%–30.5% buffer B (CI elution gradient; Figure S1A). Any remaining protein was then eluted with 100% buffer B. The Q-Sepharose gradient was run overnight at 1.0 mL/min at 4°C. CI-containing fractions were pooled based on NADH:FeCy activity and concentrated to 1.5-2.0 mL. Once it was determined that this Q-Sepharose protocol resulted in a significant proportion of CI in the high-salt wash (Figure S1A), the CI elution gradient was extended to 22%–60% buffer B over 450 mL, resulting in the isolation of two distinct peaks containing NADH:FeCy (CI) activity (Figure S1B). As a further optimization, the elution gradient was changed to 22%–50% over 250 mL. This resulted in the abundant isolation of SC I+III_2 (Figure 1A). It was also essential to increase the flow rate over the column to 3.0 mL/min throughout to minimize the amount of time the protein was on the Q-Sepharose column.

Samples eluted from the Q-Sepharose column were then applied either to the same column again and run with a similar gradient to separate CI and $CIII_2$ (Figure S1C), or to a Superose 6 prep grade XK 6/100 size exclusion chromatography column equilibrated in buffer S (20 mM HEPES, pH 7.4, 2 mM EDTA, 10% glycerol, 50 mM NaCl and either 0.05% LMNG (Figure S1D) or no LMNG for the amphipol samples (Figure 1B)), and eluted overnight at 0.35 mL/min at 4°C for either step. For the amphipol sample, amphipol A8-35 was added to a final concentration of 0.3% (w/v) immediately following elution from the Q-Sepharose gradient. This mixture was then tumbled at 4°C for ~30 min before concentrating to ~1.5-2.0 mL and loading onto the Superose 6 column. Subsequent SEC runs were done using the same column either in 0.5% LMNG (Figure S1E) or in no LMNG (Figure 1C) buffer S. After elution, the fractions containing NADH:FeCy activity were pooled and concentrated. These samples were either used directly for cryoEM grid preparation or made to 30% glycerol and stored in liquid nitrogen. Samples stored in this way maintained high activity over several months.

Blue Native PAGE

Samples diluted with 4X sample buffer (50 mM BisTris-HCl, pH 7.2, 50 mM NaCl, 10% w/v glycerol, and 0.001% Ponceau S) were loaded onto a NativePAGE 3%–12% Bis-Tris Gel (Invitrogen) and run in the cold room at 4°C. The cathode buffer was 50 mM Tricine, 50 mM BisTris-HCl, pH 6.8 plus 1X NativePAGE Cathode Buffer Additive (Invitrogen) and the anode buffer was 50mM Tricine, 50 mM BisTris-HCl, pH 6.8. Gels were run at 150 V for ~60 min and then the voltage was increased to 250 V for the remainder of the run ~90 min.

Mass Spectrometry

Mass spectrometry (MS) analysis was performed essentially as previously described (Letts et al., 2016a). Briefly, polyacrylamide gel slices (1–2 mm) containing bands of the purified proteins (Figure S1H) were prepared for mass spectrometric analysis by manual *in situ* enzymatic digestion. The excised protein gel pieces were placed in wells of a 96-well microtiter plate and destained with 50% (v/v) acetonitrile and 50 mM ammonium bicarbonate, reduced with 10 mM DTT, and alkylated with 55 mM iodoacetamide. After alkylation, proteins were digested with 6 ng/μL trypsin (Promega, UK) overnight at 37°C. The resulting peptides were extracted in 2% (v/v) formic acid, 2% (v/v) acetonitrile. The digest was analyzed by nano-scale capillary LC-MS/MS using an Ultimate U3000 HPLC (ThermoScientific Dionex, San Jose, CA) to deliver a flow of ~300 nL/min. A C18 Acclaim PepMap100 5 μm, 100 μm x 20-mm nanoViper (ThermoScientific Dionex) trapped the peptides prior to separation on a C18 Acclaim PepMap100 3 μm, 75 μm x 250 mm nanoViper (ThermoScientific Dionex). Peptides were eluted with a gradient of acetonitrile. The analytical column outlet was directly interfaced via a nanoflow electrospray ionization source, with a hybrid dual pressure linear ion trap mass spectrometer (Orbitrap Velos, ThermoScientific). Data-dependent analysis was carried out, using a resolution of 30,000 for the full MS spectrum, followed by 10 MS/MS spectra in the linear ion trap. MS spectra were collected over an m/z range of 300-2000. MS/MS scans were collected using the threshold energy of 35 for collision-induced dissociation. LC-MS/MS data were then searched against a protein database (mammalian subset of UniProt KB) using the Mascot search engine program (Matrix Science, UK) (Perkins et al., 1999). Database search parameters were set with a precursor tolerance of 10 ppm and a fragment ion mass tolerance of 0.8 Da. One missed enzyme cleavage was allowed, and variable modifications for oxidized methionine and carbamidomethyl cysteine were included. MS/MS data were validated using the Scaffold program (Proteome Software) (Keller et al., 2002). All data were additionally interrogated manually. See Table S1.

Organic Phosphate Assay

Measurement of organic phosphate content was performed according to the protocol of Anderson and Davis without modification (Anderson and Davis, 1982). Lipid was extracted from protein samples (~20 μL) by addition of 200 μL of 2:1 chloroform/MeOH followed by vortexing. Then, 50 μL of 125 mM NaCl was added, and the samples were vortexed again. The phases were separated by centrifugation for 3 min at ~13,000 x g, and the lower chloroform phase was desiccated and used for measurement. See Table S2.

Activity Assays

CI NADH:FeCy activity was measured by spectroscopic observation of NADH oxidation at 340 nm wavelength using a Shimadzu UV-2600 UV-VIS spectrophotometer with CPS-100 thermoelectrically temperature-controlled cell positioner and modified by Rank Brothers for continuous sample stirring. SC I+III_2 NADH:cyt *c* oxidoreductase activity was measured at 340 nm for NADH oxidation and 550 nm for cyt *c* reduction using either the same Shimadzu UV-2600 UV-VIS spectrophotometer as above or a Spectramax M2e Plate-Reader (Molecular Devices). The buffer used for NADH:FeCy activity was 20 mM HEPES, pH 7.4, 50 mM NaCl, 2 mM EDTA, 0.1% DDM, 100 μM NADH and 1 mM potassium ferricyanide (KFeCy). NADH:Cyt *c* oxidoreductase activity of the isolated SC I+III_2 were done in the standard buffer (SB): 100 mM HEPES, pH 7.4, 50 mM NaCl, 10% glycerol and 4 μM KCN, 50 U/mL SOD; or

lipid-detergent (LD) buffer: SB plus 0.1% CHAPS, 0.1% LMNG, 0.25 mg/mL 4:1 DOPC:CL and 10 μM BSA. Both NADH oxidation activity (340 nm, top) and cyt *c* reduction activity (550 nm, bottom) were monitored in separate experiments for each condition. Activity is reported as Units (1 U = 1 μmol substrate per min per mg of SC I+III_2). Where Michaelis-Menten curve fitting is appropriate, the V_{max} and apparent K_m are shown on the plots ± standard error. Where the concentration-activity curves are not hyperbolic, the maximum activity (Act_{max}) is shown, mean ± SEM. SC I+III_2 was added to a final concentration of 1.3-10 nM depending on the experimental conditions and amount of activity. Measurements done using the Shimadzu UV-2600 UV-VIS spectrophotometer were carried out using disposable 2 mL polystyrene cuvettes (LLG Labware) with constant stirring. Measurements done using the Spectramax M2e Plate-Reader were done using 200 μL reaction buffer in 96-well plates with 10-20 s of stirring before beginning to record. All activity measurements were performed at 30°C and initiated by addition of NADH. Concentrations of NADH stocks were determined using the Shimadzu UV-2600 UV-VIS spectrophotometer and the known extinction co-efficient of 6.22 mM^{-1} cm^{-1}. These standards were then used to generate a standard curve on the Spectramax M2e Plate-Reader under experimental conditions in the presence of 100 μM cyt *c*. Standard curves for oxidized and reduced (by dithionite) cyt *c* were also generated in a similar fashion *in situ* under experimental conditions in the presence of 100 μM NADH. All protein concentrations were determined with the Pierce bicinchoninic acid (BCA) assay kit using BSA standards (Thermo Fisher, Waltham, MA). All samples for protein concentration determination were diluted at least 10-fold into 20 mM HEPES, pH 7.4, 0.1% DDM, and 50 mM NaCl buffer to reduce interference from glycerol in the sample buffers. See Figures 2, 3, and S2 and Table S3.

CryoEM grid preparation, optimization, and data acquisition

Initial grid preparation using the amphipol samples resulted in only broken particles. We hypothesized that this was due to instability of the SC at the large air-water interface present during blotting (Glaeser and Han, 2017; Glaeser et al., 2016) and found it necessary to add back detergent to reduce the surface tension and preserve the particles. Using the polyoxyether detergent Brij-35 generated high-quality grids with a high-density of particles in the holes, without solubilizing SCs. The final optimal blotting conditions were from application of 2.7 μL aliquots of ~2 mg/mL amphipol stabilized SC I+III_2 (in 250 mM NaCl, 20 mM HEPES, pH 7.7, 0.02% Brij-35) to glow-discharged holey carbon grids (Quantifoil R0.6/1 CU) followed by blotting for 30 s at 4°C, 95% humidity and flash freezing in liquid ethane using an FEI Vitrobot IV. CryoEM data acquisition was performed on a 300 kV FEI Titan Krios electron microscope with a Falcon II camera. Automated data collection was performed with the FEI EPU package. Micrographs were recorded at a nominal magnification of 100,000 X, resulting in a pixel size of 1.40 Å. Defocus values varied from −1.5 to −3.0 μm. The dose rate was ~50 electrons per pixel per second. Exposures of 2 s were dose-fractionated into 34 frames, leading to a dose of 1.5 electrons per $Å^2$ per frame and a total accumulated dose of 51 electrons per $Å^2$. A total of 1,854 micrographs were collected. See Table S4.

Image processing and 3D reconstruction

All processing steps were done using RELION 2.0 and 2.1 (Kimanius et al., 2016) unless otherwise stated. MotionCor2 (Zheng et al., 2017) was used for whole-image drift correction of each micrograph. Contrast transfer function (CTF) parameters of the corrected micrographs were estimated using Gctf and refined locally for each particle (Zhang, 2016). Automated particle picking in Relion resulted in ~400 k particles after manual curating. The particles were extracted using 400^2 pixel box and sorted by reference-free 2D classification followed by re-extraction at 512^2 pixel box and initial 3D classification with a regularization parameter T of 4 and a 30 Å low-pass filtered initial model generated from the PDB coordinates of CI and $CIII_2$ fit into the low resolution reconstruction of the ovine respirasome (Letts et al., 2016b). This initial processing resulted in ~250 k particles of good quality, which separated into several classes containing SC I+III_2, isolated CI or isolated $CIII_2$ (Figure S4C). As the SEC trace of the amphipol-stabilized SC I+III_2 was monodisperse (Figure 1), we concluded that the isolated CI and $CIII_2$ particles were generated during the grid preparation and plunge-freezing process. The isolated CI and SC I+III_2 particles were separated and further classified to remove poor particles. At this stage, several distinct classes of the SC and isolated CI were identified and refined, resulting in reconstructions with nominal resolutions of 4.2 Å for three states and 4.6 Å for a fourth state according to the gold-standard FSC criteria.

Nonetheless, it was clear that the relative orientation between the two arms of CI and between CI and $CIII_2$ in SC I+III_2 was variable, which resulted in lower resolution at the edges of the CI peripheral arm and $CIII_2$ (Figure 7E; Figure S7) and likely limited the nominal resolution of the reconstructions. To improve the maps, focused refinements were independently performed around three regions of the SC: the CI peripheral arm, the CI membrane arm and $CIII_2$ (Figure S6). For the CI peripheral arm and CI membrane arm refinements, isolated CI particles were also included. After initial 3D classification, all of the CI-containing particles (i.e., isolated CI and SC I+III_2) were combined and aligned via the peripheral arm of CI (Figure S5A). An additional round of 3D classification focused on the peripheral arm was performed to remove any remaining poor-quality particles. Interestingly, a small class of particles was identified that lacked the NADH-binding N-module of the peripheral arm (Figure S5A). The N-module is added to CI in the final stages of assembly (Formosa et al., 2018; Guerrero-Castillo et al., 2017) and its association may be dynamically controlled in response to ROS (Guarás et al., 2016). A class of particles lacking the N-module was also observed in the bovine respirasome structure (Sousa et al., 2016), suggesting that a sub-population of CI lacking the N-module is a common feature in mammalian mitochondria.

When we used the best class of 178,121 particles for 3D-refinement around the CI peripheral arm, we achieved a reconstruction at 3.8 Å, which allowed for atomic modeling of the CI peripheral arm (Figures S5A and S5D; Table 1). This class of 178,121 particles also defined the set of high-quality CI-containing particles that we used for all further CI 3D classifications (see below). Next, we aligned these particles via the CI membrane arm and performed an additional 3D classification to define a best-matching set of CI membrane

arm particles (Figure S5C). We further refined the best classes from this classification (174,334 particles) to generate a reconstruction at 3.9 Å resolution, again allowing for atomic modeling of the CI membrane arm (Figures S5C and S5D; Table 1). As these sets of particles contained both isolated CI and SC I+III_2, we performed the focused refinement for $CIII_2$ with the original set of 120,596 particles identified in the original SC 3D classification (Figure S5B). After an additional round of 3D classification, we identified a set of 102,314 particles that, once refined, generated a 3.9 Å reconstruction around $CIII_2$ (Figures S5B and S5D). It was found that re-centering and re-boxing the particles around the CI peripheral arm and $CIII_2$ using the program REP (D'Imprima et al., 2017) improved map quality. This was not the case for the membrane arm which sits very near to the center of mass of the SC.

Finally, to better separate particles based on the difference between the CI membrane and peripheral arms, we developed a focus-revert-classify strategy in which the particles were first aligned by the peripheral arm of CI and then separated by 3D-classification with a mask around the membrane arm of CI without angular or translational searches (Figure 5). By pre-aligning particles by the peripheral arm, the small differences in the positions of the membrane arms should be maximized, thus facilitating efficient separation during 3D-classification without rotational or translational searches (Figure 5A). The peripheral arm was chosen for initial alignment as it contains all of the FeS cluster co-factors of CI, which generate strong peaks in the cryoEM density map. This strategy using ten classes resulted in the separation of at least seven distinct states of CI: one closed state and six open states (Figure S6A). The closed state and open states 1-3 each had significantly fewer particles than the original four SC states identified, but refined to similar or improved nominal resolutions, indicating that a more homogeneous set of particles for each state had been identified by this classification strategy. The nominal resolution of the seven classes ranged from 4.1-6.5 Å according to the gold-standard FSC criteria. Two additional classes at worse than 7.0 Å resolution were also generated but not analyzed further due to their poorer quality. Since no rotational or translational searches were performed, one class consisted of particles that did not align well with any of the other classes or each other and hence averaged out resulting in very weak and noisy density.

All local resolution maps were generated using BlockRes in Relion2.0 (Kimanius et al., 2016).

Atomic model building and refinement

Starting models for isolated ovine CI (Fiedorczuk et al., 2016) and bovine $CIII_2$ (Iwata et al., 1998), corrected for the ovine sequence, were used. These models were split and fit into the highest-resolution focused refinement maps for separate atomic model building of the CI peripheral arm, CI membrane arm and $CIII_2$. Improved density in many regions relative to the previous maps of isolated CI allowed for the building of improved atomic models in COOT (Emsley et al., 2010). Real-space refinement of the model was done in PHENIX (Adams et al., 2010) and group atomic displacement parameters (ADPs) were refined in reciprocal space. Due to the unique features of cryoEM density (Coulomb potential density) relative to the electron density generated from X-ray diffraction data, such as frequent missing side chains for acidic amino acids caused by radiation damage (Baker and Rubinstein, 2010), we implemented a novel refinement protocol. First, we investigated whether the default values for group ADP refinements established for X-ray diffraction may be too stringent given the uncertainty in side chain position caused by radiation damage. Due to the moderate resolution of the reconstructions, individual (per-atom) ADP refinement was not performed, but, rather, group ADPs (one value for main chain atoms and another for side chain atoms) were assigned. ADP refinement in PHENIX is done using local sphere restrains for each atom a_i with three adjustable parameters: the sphere radius, all atoms within this radius of atom a_i will be used to constrain its ADP value (default value 5 Å); the distance power, this parameter determines the degree to which the distance between two atoms a_i and a_j within the sphere radius influences the restraint (X-ray refinement default value: 1.69); and the average power, which determines the degree to which the ADP is restrained by the average value of ADP between atom a_i and a_j (X-ray refinement default value: 1.03) (Afonine et al., 2005). As has been pointed out previously, the ADP can represent the convolution of two types of uncertainty: 1) the dispersion of the atom position in the molecules (which may be the result of temperature-dependent atomic vibrations or static disorder in a crystal lattice) and 2) the uncertainty of the experimenter's knowledge about the atom position (Merritt, 2012). The second type of uncertainty can be taken care of by occupancy refinement, but that is problematic at current cryoEM data resolutions. Therefore, we choose to optimize ADP refinement specifically for cryoEM data. We found that the default values underestimated the ADPs for side chains with poor density (such as Asp and Glu) when the density for the main chain atoms was well defined. We therefore tested an array of APD refinement parameters and found that decreasing the sphere radius to 2.5 Å and increasing the distance power to 2.3 without changing the average power resulted in more reasonable, higher group ADPs for side chains with poorly defined cryoEM density while maintaining overall consistency throughout the structure. Another complication arising from refinement of models with poor side chain density for specific residues is that, unless the ADP is calculated before coordinate refinement, the refinement will attempt to place the side chain into the nearest main chain density displacing the main chain atoms and distorting the local geometry. We therefore implemented a refinement protocol in which, after manual model building in COOT, a single macro cycle of ADP refinement was performed in PHENIX (using the new defaults above) before coordinate refinement was performed. This allowed the ADP for side chains with poor cryoEM density to rise and hence reduce their propensity to displace main chain atoms during global minimization. The single cycle of group ADP refinement was followed by three cycles of global minimization, followed by an additional cycle of group ADP refinement and finally three cycles of global minimization. This refinement protocol resulted in models with improved geometry, reasonable group ADP values and better correlation with the cryoEM maps. Additionally, the ADP values of the model agreed qualitatively more with the local resolution of the cryoEM reconstructions (Figure S7). The script to run the protocol is available from authors on request.

In previous work on isolated ovine CI, subunit NDUFA11 (B14.7), TMH4 of ND6, the C-terminal TMH16 of ND5 and the C-terminal half of the ND5 lateral helix were disordered (Fiedorczuk et al., 2016). Given that these regions were clearly resolved in the focused maps of the CI membrane arm (Figure S5C), we could now build their model at atomic resolution. Additionally, in the previous structure, subunit NDUFB8 (ASHI) was built mostly as poly-alanine (Fiedorczuk et al., 2016). In contrast, we now present it in full atomic detail here. We also made significant improvements to the models of subunits NDUFB4 (B15), NDUFB9 (B22) and NDUFB7 (B18). Besides these major improvements, we also achieved minor improvements throughout the structure due to the high quality of the focused refinements (Figure S5). Overall, the CI model presented here in the closed SC I+III_2 is > 95% atomic and the remaining unbuilt residues are almost entirely found at disordered regions on the N- and C-termini of supernumerary subunits (Table S5). The overall structure of $CIII_2$ is similar to what has been previously reported for isolated $CIII_2$ and in the respirasome (Guo et al., 2017; Huang et al., 2005; Iwata et al., 1998; Letts et al., 2016b; Wu et al., 2016). One major difference in our structure is the lack of density for the single TM helix subunit UQCR11. UQCR11, which is located at the periphery of the Q-binding cavities on either side of the $CIII_2$ dimer, is added late in $CIII_2$ assembly (Smith et al., 2012), is not required for $CIII_2$ activity and is easily lost by chromatographic de-lipidation of the complex (Huang et al., 2005; Hunte et al., 2000; Schägger et al., 1990). Although UQCR11 is considered an authentic subunit of $CIII_2$, like many of the respiratory chain supernumerary subunits, its role is not well understood (Smith et al., 2012). UQCR11 was identified in our mass spec analysis (Table S1), which suggests that it is either sub-stoichiometric or disordered.

Due to low resolution, the focus-revert-classify reconstructions at $\geq$ 7.0 Å were not used in detailed structural comparisons, and poly-alanine models were only rigid-body fit into the reconstructions at 5.7-6.5 Å. We refined atomic models only for the four sub-4.5 Å reconstructions: the closed state and open states 1-3 (Figure 5B; Figure S6A).

QUANTIFICATION AND STATISTICAL ANALYSIS

All reported p values are from two-tailed Student's t test for independent measures of unequal variance. Resolution estimations of cryoEM density maps are based on the 0.143 Fourier Shell Correlation (FSC) criterion (Chen et al., 2013; Rosenthal and Henderson, 2003).

DATA AND CODE AVAILABILITY

Data Resources

The accession numbers for the atomic coordinates reported in the paper are PDB: 6Q9D, 6Q9B, 6QE9, 6QBX, 6QC3, 6QC2, 6QC4, 6QA9, 6QC5, 6QC6, 6QC8, 6QC7, 6QC9, 6QCA and 6QCF.

The accession numbers for the EM density maps reported in this paper are EMDB: 4480, 4479, 4481, 4493, 4495, 4494, 4496, 4482, 4497, 4498, 4500, 4499, 4501, 4502, 4505, 4506 and 4507.

Molecular Cell, Volume *75*

Supplemental Information

Structures of Respiratory Supercomplex I+III$_2$

Reveal Functional and Conformational Crosstalk

James A. Letts, Karol Fiedorczuk, Gianluca Degliesposti, Mark Skehel, and Leonid A. Sazanov

SUPPLEMENTAL INFORMATION

Structures of Respiratory Supercomplex I+III$_2$ Reveal Functional and Conformational Crosstalk

James A. Letts, Karol Fiedorczuk, Gianluca Degliesposti, Mark Skehel and Leonid A. Sazanov

LIST OF SUPPLEMENTAL INFORMATION

Supplemental Figures

Figure S1. Initial isolation and separation of SC I+III$_2$, Related to Figure 1

Figure S2. Effect of PO_4 buffer on isolated SC I+III$_2$ oxidoreductase activity, Related the Figure 2

Figure S3. Inhibition of SC I+III$_2$ activities by known CI and CIII$_2$ inhibitors, Related to Figure 3

Figure S4. Initial cryo-EM processing steps, Related to Figure 4

Figure S5. Focused refinements, Related to STAR Methods

Figure S6. Focus-Revert-Classify strategy to separate different classes of CI, Related to Figure 5

Figure S7. Examples of match between local resolution and model ADPs for isolated CI and SC I+III$_2$, Related to Figure 7 and STAR Methods

Supplemental Tables

Table S1. Mass Spectrometry identification for subunits of ovine SC I+III$_2$, CI and CIII$_2$, Related to Figure 1

Table S2. Lipid content of isolated SC I+III$_2$ particles compared to mitochondrial membranes, Related to STAR methods

Table S3. K_{cat} for CI and CIII$_2$ within the SC and Rate Coupling, Related to Figures 2 and 3

Table S4. Cryo-EM data collection, Related to Figure 4

Table S5. SC I+III$_2$ closed model overview, Related to STAR Methods

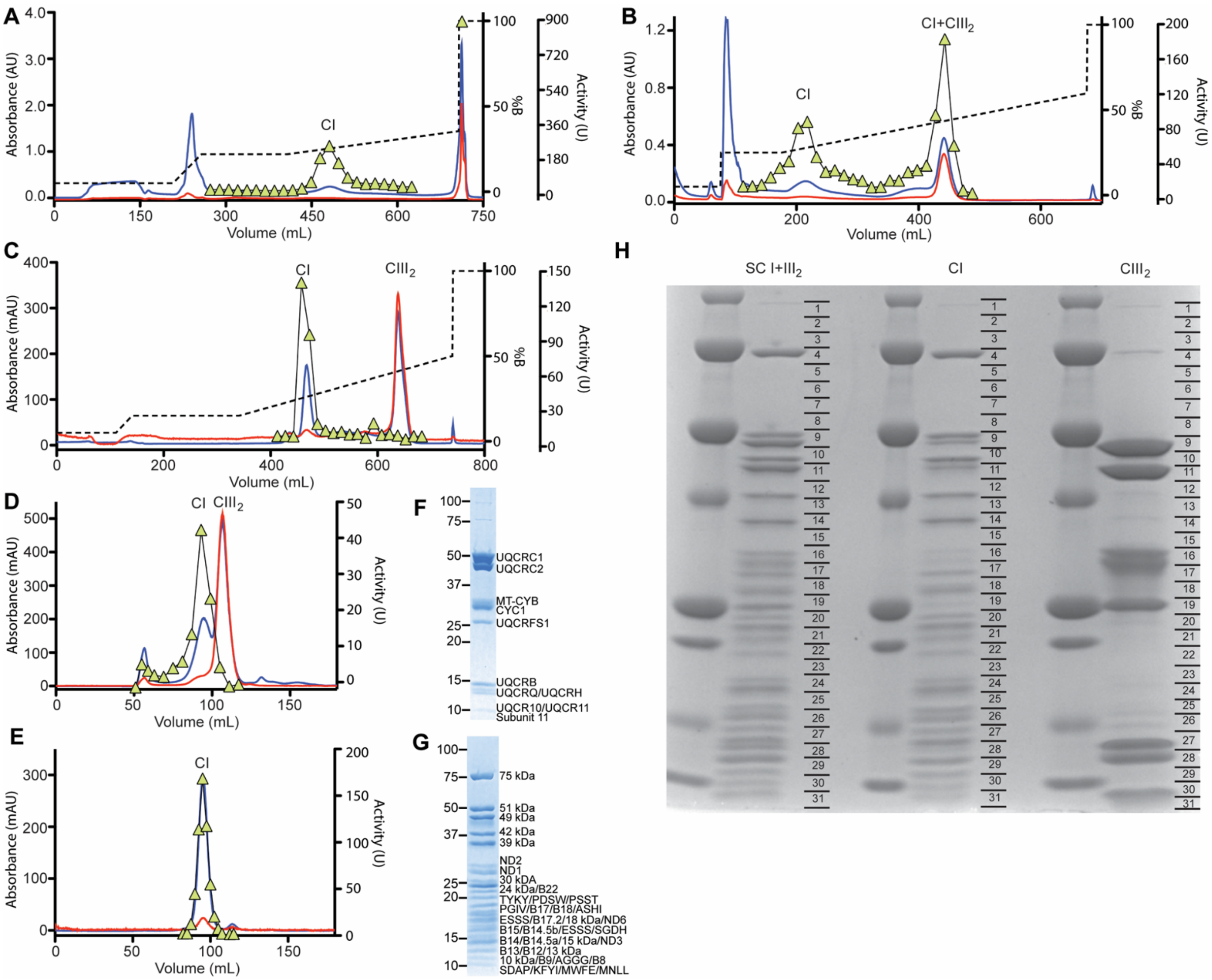

Figure S1. Initial isolation and separation of SC I+III$_2$, Related to Figure 1

(A) Q-Sepharose anion-exchange column chromatogram of LNMG-extracted washed mitochondrial membranes. In all panels the chromatograms show: A_{280}, blue line; A_{420}, red line; percent buffer B gradient (containing 1M NaCl), dashed black line; and CI NADH:FeCy activity, lime green triangles. Unlike purifications in DDM, high CI NADH:FeCy activity was seen in the high-salt wash. (B) Altering the gradient profile resulted in a second peak of NADH:FeCy activity associated with high A_{420} signal, indicating the presence of heme groups. (C) Re-running the second peak from (B) over anion-exchange resulted in the separation of peaks containing NADH:FeCy activity and most of A_{420} signal alone. (D) Running the second peak from (B) over size exclusion chromatography column also resulted in the separation of peaks containing NADH:FeCy activity and most of A_{420} signal alone. (E) Running of peak containing NADH:FeCy activity from (C) resulted in highly enriched sample of CI. (F) SDS-PAGE gel of A_{420} containing peak from (C) with CIII$_2$ subunits identified by mass spectrometry (MS). (G) SDS-PAGE gel of NADH:FeCy activity containing peak from (E) with CI subunits identified by MS. (H) SDS-PAGE showing SC I+III$_2$ sample in amphipol A8-35 (**Figure 1**C), isolated CI (**Figure S1**E) and isolated CIII$_2$ (**Figure S1**C). Each lane was cut into 31 approximately even bands (indicated on right-hand side of each lane), which were each subjected to MS analysis for protein identification. The results of MS analysis are shown in **Table S1** and the corresponding band for each subunit is indicated. The total amount of protein loaded in the gel was not controlled

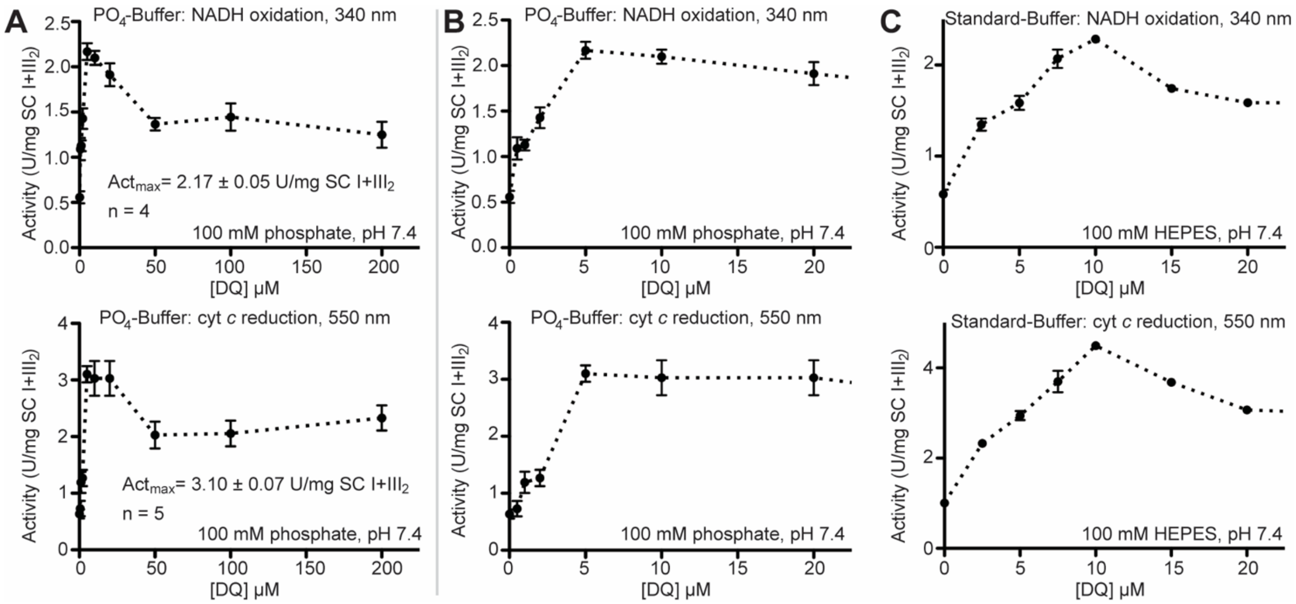

Figure S2. Effect of PO_4 buffer on isolated SC I+III_2 oxidoreductase activity, Related the Figure 2

(A) [DQ]-activity curves monitoring NADH oxidation (top) and cyt *c* reduction (bottom) in 100 mM PO_4 buffer with 100 µM NADH and 100 µM cyt *c*. (B) Expansion of x-axis (0-20 µM DQ) for [DQ]-activity curves from **Figure S2**A, NADH oxidation (top) and cyt *c* reduction (bottom). (C) Expansion of x-axis (0-20 µM DQ) for [DQ]-activity curves from **Figure 2**D, NADH oxidation (top) and cyt *c* reduction (bottom). Activity is reported as Units (1 U = 1 µmol substrate/min per mg of SC I+III_2). Data plotted as mean ± SEM. Regardless of the broader high-activity range in the phosphate buffer, the overall cyt *c* reduction activity was lower compared to HEPES. This is likely due to the known strong dependence of the interaction between $CIII_2$ and cyt *c* on ionic strength. Therefore, HEPES buffer was used throughout for further activity measurements.

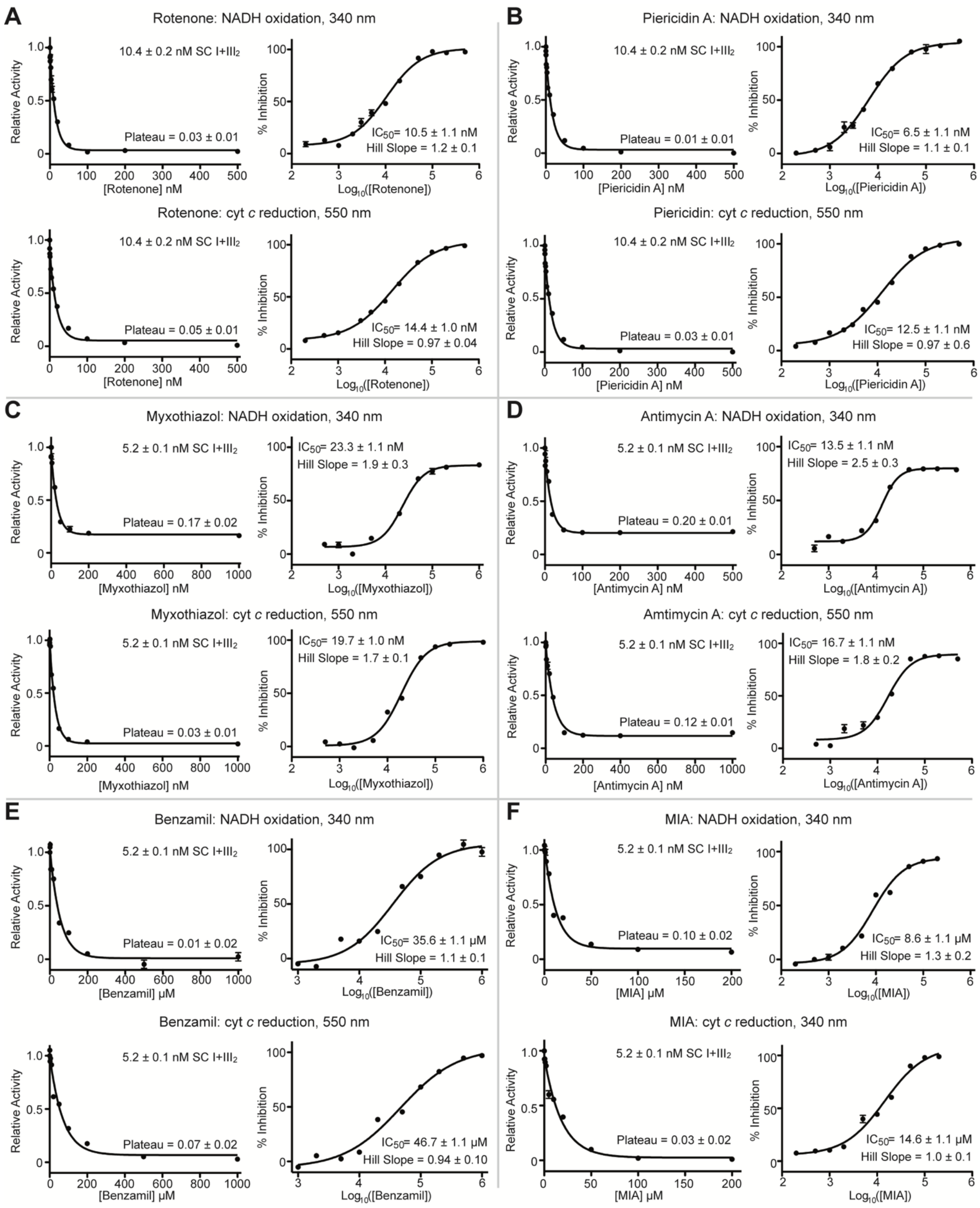

A
Rotenone: NADH oxidation, 340 nm
10.4 ± 0.2 nM SC I+III2
Plateau = 0.03 ± 0.01
IC50= 10.5 ± 1.1 nM
Hill Slope = 1.2 ± 0.1
[Rotenone] nM
Log10([Rotenone])
Rotenone: cyt c reduction, 550 nm
10.4 ± 0.2 nM SC I+III2
Plateau = 0.05 ± 0.01
IC50= 14.4 ± 1.0 nM
Hill Slope = 0.97 ± 0.04
B
Piericidin A: NADH oxidation, 340 nm
10.4 ± 0.2 nM SC I+III2
Plateau = 0.01 ± 0.01
IC50= 6.5 ± 1.1 nM
Hill Slope = 1.1 ± 0.1
[Piericidin A] nM
Log10([Piericidin A])
Piericidin: cyt c reduction, 550 nm
10.4 ± 0.2 nM SC I+III2
Plateau = 0.03 ± 0.01
IC50= 12.5 ± 1.1 nM
Hill Slope = 0.97 ± 0.6
C
Myxothiazol: NADH oxidation, 340 nm
5.2 ± 0.1 nM SC I+III2
Plateau = 0.17 ± 0.02
IC50= 23.3 ± 1.1 nM
Hill Slope = 1.9 ± 0.3
[Myxothiazol] nM
Log10([Myxothiazol])
Myxothiazol: cyt c reduction, 550 nm
5.2 ± 0.1 nM SC I+III2
Plateau = 0.03 ± 0.01
IC50= 19.7 ± 1.0 nM
Hill Slope = 1.7 ± 0.1
D
Antimycin A: NADH oxidation, 340 nm
5.2 ± 0.1 nM SC I+III2
Plateau = 0.20 ± 0.01
IC50= 13.5 ± 1.1 nM
Hill Slope = 2.5 ± 0.3
[Antimycin A] nM
Log10([Antimycin A])
Amtimycin A: cyt c reduction, 550 nm
5.2 ± 0.1 nM SC I+III2
Plateau = 0.12 ± 0.01
IC50= 16.7 ± 1.1 nM
Hill Slope = 1.8 ± 0.2
E
Benzamil: NADH oxidation, 340 nm
5.2 ± 0.1 nM SC I+III2
Plateau = 0.01 ± 0.02
IC50= 35.6 ± 1.1 μM
Hill Slope = 1.1 ± 0.1
[Benzamil] μM
Log10([Benzamil])
Benzamil: cyt c reduction, 550 nm
5.2 ± 0.1 nM SC I+III2
Plateau = 0.07 ± 0.02
IC50= 46.7 ± 1.1 μM
Hill Slope = 0.94 ± 0.10
F
MIA: NADH oxidation, 340 nm
5.2 ± 0.1 nM SC I+III2
Plateau = 0.10 ± 0.02
IC50= 8.6 ± 1.1 μM
Hill Slope = 1.3 ± 0.2
[MIA] μM
Log10([MIA])
MIA: cyt c reduction, 340 nm
5.2 ± 0.1 nM SC I+III2
Plateau = 0.03 ± 0.02
IC50= 14.6 ± 1.1 μM
Hill Slope = 1.0 ± 0.1
Relative Activity
% Inhibition

Figure S3. Inhibition of SC I+III_2 activities by known CI and $CIII_2$ inhibitors, Related to Figure 3

Relative inhibition vs. concentration (left) and percent inhibition vs. $\log_{10}$([inhibitor]) (right) are shown for inhibition of both NADH-oxidation (top) and cyt *c*-reduction (bottom) for each inhibitor. Inhibition was measured with either ~10 nM or ~5 nM SC I+III_2 as indicated for each panel. (A) The CI inhibitor rotenone gave an IC_{50} of 10.5 ± 1.1 nM for NADH oxidation and 14.4 ± 1.0 nM for cyt *c* reduction, with Hill coefficients of 1.2 ± 0.1 and 0.97 ± 0.04 respectively, consistent with the single known rotenone binding site. (B) The CI inhibitor piericidin A gave an IC_{50} of 6.5 ± 1.1 nM for NADH oxidation and 12.5 ± 1.1 nM for cyt *c* reduction, with Hill coefficients of 1.1 ± 0.1 and 0.97 ± 0.6 respectively. (C) The $CIII_2$ Q_P-site inhibitor myxothiazol inhibited 97 % of the cyt *c*-reduction activity at 1 μM with an IC_{50} of 19.7 ± 1.0 nM. However, myxothiazol only inhibited ~83 % of the NADH-oxidation activity, indicating that slow oxidation of NADH is possible under these conditions likely via exchange of DQH_2 and DQ between the SC particles and bulk pool (**Figure 3**L). The IC_{50} for the NADH-oxidation activity is consistent with that for the cyt *c*-reduction activity, at 23.3 ± 1.1 nM and both activities show consistent Hill coefficients of 1.9 ± 0.3 and 1.7±0.1, respectively. (D) The $CIII_2$ Q_N-site inhibitor antimycin A. Although strong inhibition is observed, neither the NADH-oxidation nor the cyt *c*-reduction activities show complete inhibition. Similar to myxothiazol, antimycin A inhibits ~80 % of the NADH-oxidation activity at 500 nM antimycin A, with IC_{50} of 13.5 ± 1.1 nM and a Hill coefficient of 2.5 ± 0.3. Antimycin A blocks less of the cyt *c*-reduction activity compared to myxothiazol, with ~88 % inhibition at 1 μM antimycin A, but the IC_{50} of 16.7 ± 1.1 nM and Hill coefficient of 1.8 ± 0.2 are consistent with the inhibition of NADH-oxidation. Inhibition of $CIII_2$ with antimycin A is known to result in increased ROS production at the Q_P site. Due to the large amount of SOD present in our reaction buffer (50 U/mL), it is unlikely that the source of residual cyt *c* reduction would be via direct reduction of cyt *c* by superoxide. More likely an electron from $CoQH_2$ is able to transfer through $CIII_2$, via UQCRFS1 and CYC1, to cyt *c*, reducing cyt *c* at the native CYC1 binding site. The $CoQ^{\bullet-}$ thus generated at the Q_P-site would be converted back to CoQ through reaction with O_2. However, more experiments are needed to confirm this hypothesis. One limitation of our isolated system, due to the lack of sealed membrane, is the absence of any membrane potential ($\Delta\psi$) or proton gradient (ΔpH). Therefore, although we can measure the oxidase activity of CI we are unable to measure its coupled H^+-pumping activity. This precludes direct demonstration that CI in the amphipol-stabilized SC I+III_2 maintains strong coupling between electron transfer and H^+-pumping. However, coupling can be indirectly measured using known amiloride Na^+/H^+-antiporter inhibitors that also inhibit CI. (E) The ameloride inhibitor benzamil gave an IC_{50} for NADH-oxidation of 35.6 ± 1.1 μM and (F) the ameloride inhibitor MIA gave an IC_{50} of 8.6 ± 1.1 μM. While these data are indicative of coupling in CI, they must be interpreted with caution as the binding site of the amilorides has not been definitively determined. Data are represented as mean ± SEM.

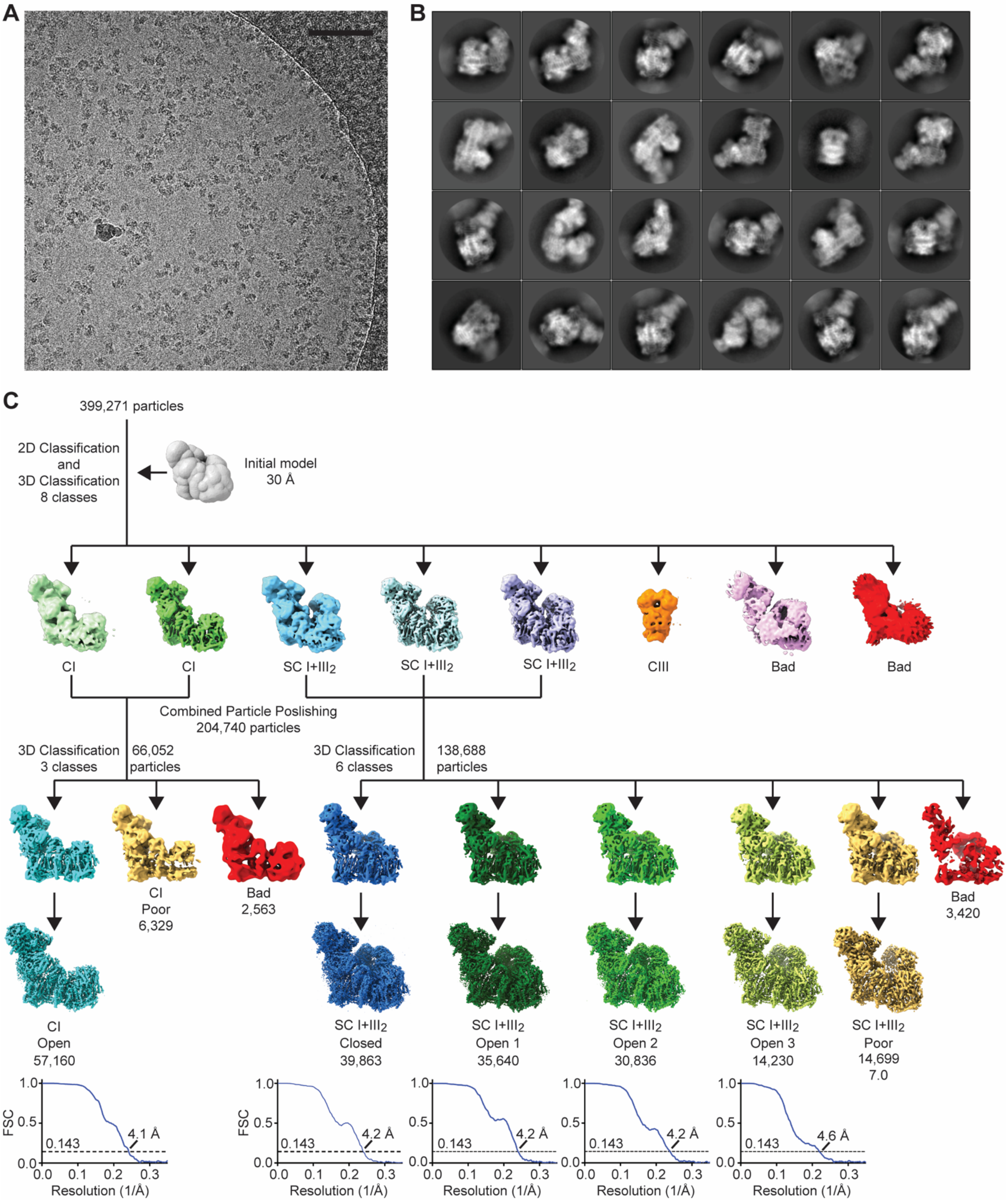

A
B
C
399,271 particles
2D Classification
and
3D Classification
8 classes
Initial model
30 Å
CI
CI
SC I+III2
SC I+III2
SC I+III2
CIII
Bad
Bad
Combined Particle Poslishing
204,740 particles
3D Classification
3 classes
66,052
particles
3D Classification
6 classes
138,688
particles
CI
Poor
6,329
Bad
2,563
Bad
3,420
CI
Open
57,160
SC I+III2
Closed
39,863
SC I+III2
Open 1
35,640
SC I+III2
Open 2
30,836
SC I+III2
Open 3
14,230
SC I+III2
Poor
14,699
7.0
FSC
0.143
4.1 Å
4.2 Å
4.2 Å
4.2 Å
4.6 Å
Resolution (1/Å)

Figure S4. Initial cryo-EM processing steps, Related to Figure 4

(A) Representative micrograph of the 1,788 micrographs collected. Scale bar is 100 nm (B) Representative 2D class averages obtained from reference-free classification in Relion (Kimanius et al., 2016). (C) Initial classification and refinement procedures used in this study. The gold-standard FSC curves are shown below their respective final reconstructions.

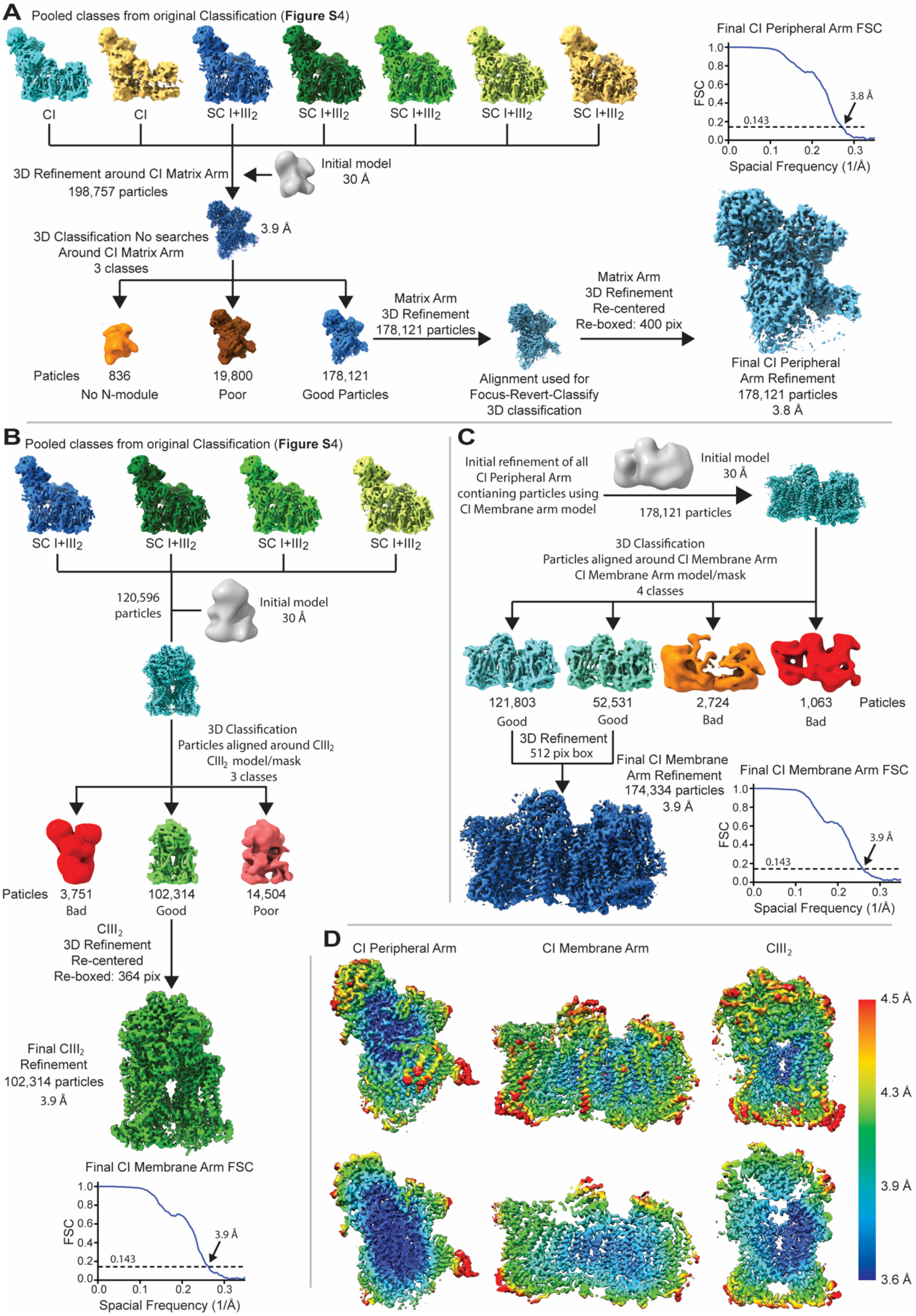
A Pooled classes from original Classification (Figure S4)
CI
CI
SC I+III2
SC I+III2
SC I+III2
SC I+III2
SC I+III2
Initial model 30 Å
3D Refinement around CI Matrix Arm 198,757 particles
3.9 Å
3D Classification No searches Around CI Matrix Arm 3 classes
Paticles 836 No N-module
19,800 Poor
178,121 Good Particles
Matrix Arm 3D Refinement 178,121 particles
Alignment used for Focus-Revert-Classify 3D classification
Matrix Arm 3D Refinement Re-centered Re-boxed: 400 pix
Final CI Peripheral Arm Refinement 178,121 particles 3.8 Å
Final CI Peripheral Arm FSC
FSC
Spacial Frequency (1/Å)
0.143
3.8 Å
B Pooled classes from original Classification (Figure S4)
SC I+III2
SC I+III2
SC I+III2
SC I+III2
120,596 particles
Initial model 30 Å
3D Classification Particles aligned around CIII2 CIII2 model/mask 3 classes
Paticles 3,751 Bad
102,314 Good
14,504 Poor
CIII2 3D Refinement Re-centered Re-boxed: 364 pix
Final CIII2 Refinement 102,314 particles 3.9 Å
Final CI Membrane Arm FSC
FSC
Spacial Frequency (1/Å)
0.143
3.9 Å
C Initial refinement of all CI Peripheral Arm contianing particles using CI Membrane arm model
Initial model 30 Å
178,121 particles
3D Classification Particles aligned around CI Membrane Arm CI Membrane Arm model/mask 4 classes
121,803 Good
52,531 Good
2,724 Bad
1,063 Bad
Patitcles
3D Refinement 512 pix box
Final CI Membrane Arm Refinement 174,334 particles 3.9 Å
Final CI Membrane Arm FSC
FSC
Spacial Frequency (1/Å)
0.143
3.9 Å
D CI Peripheral Arm
CI Membrane Arm
CIII2
4.5 Å
4.3 Å
3.9 Å
3.6 Å

Figure S5. Focused refinements, Related to STAR Methods

(A) All CI-containing particles from the initial classifications (Figure S4) were pooled and aligned by the CI peripheral arm. An additional round of 3D-classification around the CI peripheral arm was performed to remove any remaining poorly aligning particles. The final set of 178,121 particles was then aligned, followed by re-centering and re-boxing of the particles focused on the CI peripheral arm using REP (D'Imprima et al., 2017). The final 3D-reconstruction had a nominal resolution of 3.8 Å according to the gold-standard FSC method. (B) All good $CIII_2$ containing particles (SC) from the initial 3D-classifiactions (Figure S4C) were combined and aligned around $CIII_2$. An additional round of 3D classification was performed in order to remove any remaining poorly aligning particles. The final set of 102,314 particles was then aligned, followed by re-centering and re-boxing of the particles focused on $CIII_2$ peripheral arm using REP. All alignments were performed locally, so that overall orientation of $CIII_2$ *vs* SC was preserved. The final 3D-reconstruction had a nominal resolution of 3.9 Å according to the gold-standard FSC method. (C) The set of good CI peripheral arm containing particles were aligned by the CI membrane arm and then further classified to remove any remaining poorly aligning particles. The good CI membrane arm classes were combined into a final set containing 174,334 particles; re-centering failed to improve resolution or density features and hence was not performed for the final reconstruction. The final CI membrane arm reconstruction had a nominal resolution of 3.9 Å according to the gold-standard FSC method. (D) Local resolution maps of the CI peripheral arm (left), membrane arm (middle) and $CIII_2$ (right) showing side views (top) and slices into the protein cores (bottom).

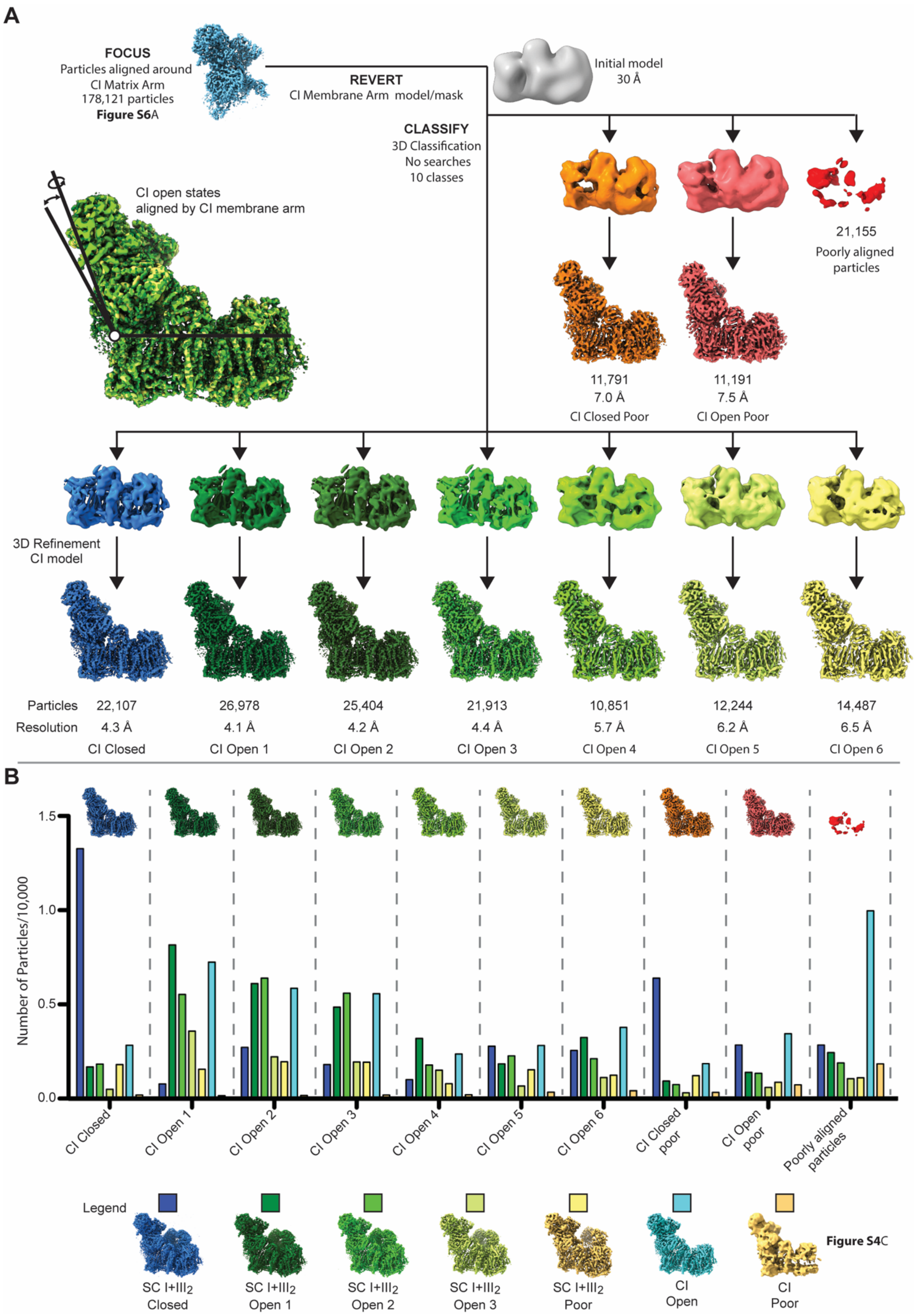

A
FOCUS
Particles aligned around
CI Matrix Arm
178,121 particles
Figure S6A
REVERT
CI Membrane Arm model/mask
Initial model
30 Å
CLASSIFY
3D Classification
No searches
10 classes
CI open states
aligned by CI membrane arm
21,155
Poorly aligned
particles
11,791
7.0 Å
CI Closed Poor
11,191
7.5 Å
CI Open Poor
3D Refinement
CI model
Particles 22,107 26,978 25,404 21,913 10,851 12,244 14,487
Resolution 4.3 Å 4.1 Å 4.2 Å 4.4 Å 5.7 Å 6.2 Å 6.5 Å
CI Closed
CI Open 1
CI Open 2
CI Open 3
CI Open 4
CI Open 5
CI Open 6
B
Number of Particles/10,000
1.5
1.0
0.5
0.0
CI Closed
CI Open 1
CI Open 2
CI Open 3
CI Open 4
CI Open 5
CI Open 6
CI Closed poor
CI Open poor
Poorly aligned particles
Legend
SC I+III2 Closed
SC I+III2 Open 1
SC I+III2 Open 2
SC I+III2 Open 3
SC I+III2 Poor
CI Open
CI Poor
Figure S4C

Figure S6. Focus-Revert-Classify strategy to separate different classes of CI, Related to Figure 5

(A) Particles aligned by the CI peripheral arm (**Figure S5**A) were classified into 10 classes using a mask for the CI membrane arm without rotational or translational searches. This resulted in the separation of seven classes of CI, which differed mainly in the angle between the membrane arm and peripheral arms (inset). The nominal resolution of these classes ranged from 4.1-6.5 Å according to the gold-standard FSC method. (B) Comparison of the original SC and isolated CI classes (**Figure 4**C) to focus-revert-classify classes with a histogram of particle counts shared between the original classes (see legend schematic) and each focus-revert-classify CI class (top, colored as in A).

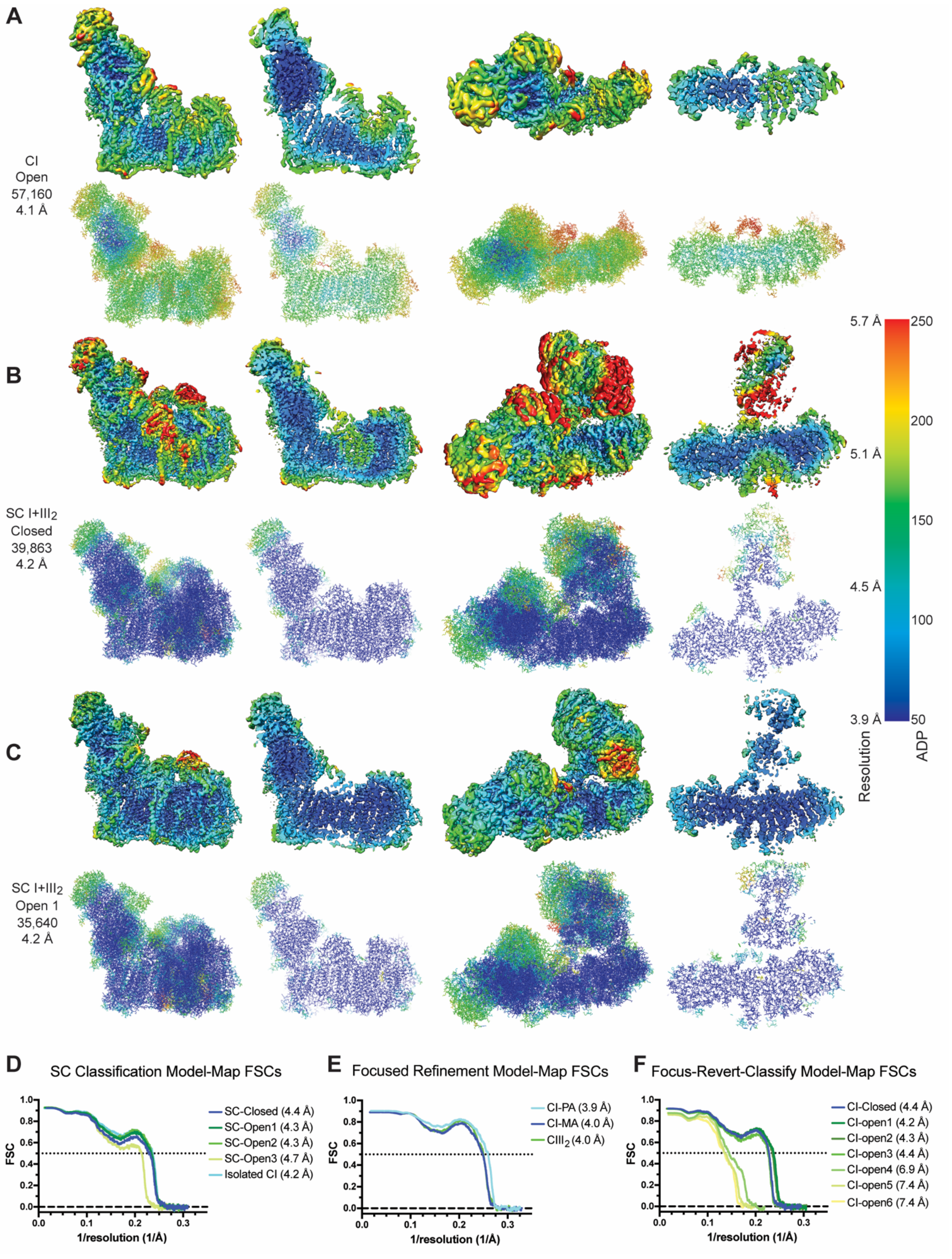
A
CI
Open
57,160
4.1 Å
B
SC I+III$_2$
Closed
39,863
4.2 Å
C
SC I+III$_2$
Open 1
35,640
4.2 Å
5.7 Å
5.1 Å
4.5 Å
3.9 Å
Resolution
250
200
150
100
50
ADP
D
SC Classification Model-Map FSCs
SC-Closed (4.4 Å)
SC-Open1 (4.3 Å)
SC-Open2 (4.3 Å)
SC-Open3 (4.7 Å)
Isolated CI (4.2 Å)
E
Focused Refinement Model-Map FSCs
CI-PA (3.9 Å)
CI-MA (4.0 Å)
CIII$_2$ (4.0 Å)
F
Focus-Revert-Classify Model-Map FSCs
CI-Closed (4.4 Å)
CI-open1 (4.2 Å)
CI-open2 (4.3 Å)
CI-open3 (4.4 Å)
CI-open4 (6.9 Å)
CI-open5 (7.4 Å)
CI-open6 (7.4 Å)
FSC
1/resolution (1/Å)

Figure S7. Examples of match between local resolution and model ADPs for isolated CI and SC I+III_2, Related to Figure 7 and STAR Methods

(A) Local resolution maps (top) and refined models (bottom) for isolated CI. Side view and slice through CI (left) and matrix view and slice through membrane domain (right) are shown. (B) As in (A) for the SC I+III_2 closed state structure. (C) As in (A) for the SC I+III_2 open 1 state structure. The maps are colored by local resolution and the models are shown as sticks and colored by ADP. (D) FSC curves between the models and final maps for the initial SC classification (see **Figure S**4). (E) FSC curves between the models and final maps for the focused refinements (see **Figure S**5). (F) FSC curves between the models and final maps for the focus-revert-classify (FRC) classes (see **Figure S**6). The resolution at FSC = 0.5 is shown for each curve in the legend as well as in **Table** 3. Models for the low-resolution (>5Å) FRC classes (CI-open4, CI-open6, and CI-open7) are all poly-alanine and were only fit into the maps as rigid bodies without additional refinement.

SUPPLEMENTARY TABLES

Table S1. Mass Spectrometry identification for subunits of ovine SC I+III$_2$, CI and CIII$_2$, Related to Figure 1

Subunit		Mature subunit size		SC I+III$_2$				CI				CIII$_2$			
Bovine name	Human name	#aa	MW (kDa)	# unique peptides	% coverage	% total spectrum	Slice with highest spectrum count	# unique peptides	% coverage	% total spectrum	Slice with highest spectrum count	# unique peptides	% coverage	% total spectrum	Slice with highest spectrum count
CI core subunits															
ND1	ND1	318	35.9	6	15	0.04	18	4	11	0.04	18	3[a]	11	0.008	18
ND2	ND2	347	39.1	2	8	0.007	18	4	19	0.02	17	3	12	0.01	17
ND3	ND3	115	13.1	1	13	0.03	27	1	13	0.02	27	1	13	0.003	27
ND4	ND4	459	52	6	18	0.04	14	6	17	0.05	14	2	6	0.006	14
ND4L	ND4L	98	10.8	-[b]	-	-	-	-	-	-	-	-	-	-	-
ND5	ND5	606	68.4	17	31	0.19	11	13	28	0.26	11	12	19	0.08	12
ND6	ND6	175	19.1	3	18	0.02	25	2	5	0.02	25	1	5	0.009	25
75 kDa	NDUFS1	704	76.9	49	77	1.5	4	41	59	1.6	4	27	42	0.51	4
49 kDa	NDUFS2	430	49.1	23	51	0.32	10	20	45	0.52	10	19	37	0.13	10
30 kDa	NDUFS3	228	26.4	21	65	0.30	18	16	55	0.33	18	12	40	0.18	18
PSST	NDUFS7	179	20.1	8	36	0.26	22	7	36	0.27	22	4	18	0.07	21
TYKY	NDUFS8	176	20.2	11	51	0.12	21	7	42	0.11	21	7	42	0.05	21
51 kDa	NDUFV1	444	48.5	33	82	0.59	9	24	60	0.67	9	45	30	0.17	8
24 kDa	NDUFV2	217	23.8	15	68	0.14	19	13	58	0.17	20	11	56	0.098	20
CI Supernumerary Subunits															
MWFE	NDUFA1	70	8.2	3	36	0.007	30	2	34	0.002	30	-	-	-	-
B8	NDUFA2	98	10.9	7	43	0.11	29	7	43	0.14	29	6	43	0.08	29
B9	NDUFA3	83	9.2	5	87	0.07	30	2	22	0.06	30	2	22	0.03	29
B13	NDUFA5	115	13.1	7	72	0.09	28	4	31	0.06	29	4	31	0.06	28
B14	NDUFA6	127	14.9	7	39	0.06	27	8	51	0.10	27	4	33	0.05	27
B14.5a	NDUFA7	112	12.4	10	54	0.13	27	10	54	0.15	27	6	53	0.07	27
PGIV	NDUFA8	171	20	8	52	0.14	24	2	43	0.061	24	7	14	0.16	23
39 kDa	NDUFA9	344	39	23	59	0.48	14	24	59	0.64	14	21	56	0.22	14
42 kDa	NDUFA10	320	36.8	24	68	0.39	12	22	58	0.42	12	15	42	0.18	12
B14.7	NDUFA11	140	14.6	7	64	0.11	27	7	64	0.11	27	3	30	0.02	27
B17.2	NDUFA12	145	17.1	15	88	0.30	25	11	66	0.19	26	7	41	0.05	25
B16.6	NDUFA13	143	16.6	15	79	0.36	26	13	79	0.18	26	9	50	0.10	25
SDAP	NDUFAB1	88	10.1	4	36	0.01	30	2	24	0.003	30	-	-	-	-
MNLL	NDUFB1	57	6.9	1	30	0.002	31	1	30	0.001	31	-	-	-	-
AGGG	NDUFB2	72	8.5	2	8	0.003	29	2	8	0.003	29	1	7	0.001	Am[c]
B12	NDUFB3	97	11	4	45	0.06	28	3	28	0.02	29	3	28	0.02	28
B15	NDUFB4	128	15	11	70	0.11	26	11	70	0.08	27	7	51	0.05	26
SGDH	NDUFB5	143	16.7	10	37	0.17	25	9	37	0.21	25	6	26	0.07	25
B17	NDUFB6	127	15.4	7	51	0.07	24	7	45	0.07	24	3	29	0.01	24
B18	NDUFB7	136	16.3	11	72	0.17	24	10	60	0.23	24	9	59	0.05	23
ASHI	NDUFB8	158	18.8	9	60	0.07	24	7	53	0.1	23	5	44	0.01	23
B22	NDUFB9	178	21.6	8	56	0.13	20	5	28	0.06	21	4	20	0.05	20
PDSW	NDUFB10	175	20.8	11	59	0.19	21	11	54	0.19	20 (21)	9	53	0.13	21
ESSS	NDUFB11	125	14.4	8	52	0.20	24	6	45	0.15	24	3	23	0.03	24
KFYI	NDUFC1	49	5.8	1	20	0.006	30	1	20	0.001	29	1	20	0.004	31

B14.5b	NDUFC2	120	14.2	9	60	0.09	26	8	59	0.12	27	5	32	0.05	26
18 kDa (AQDQ)	NDUFS4	133	15.3	11	62	0.20	25	7	46	0.21	25	5	41	0.03	25
15 kDa (PFFD)	NDUFS5	105	12.4	6	47	0.05	27	6	44	0.06	27	3	14	0.007	26
13 kDa	NDUFS6	96	10.6	8	79	0.09	28	4	44	0.02	27	6	54	0.01	28
10 kDa	NDUFV3	75	8.4	1	23	0.002	29	1	23	0.007	29	1	23	0.002	28
CIII$_2$															
Core 1	UQCRC1	446	49.3	27	59	1.0	9	18	46	0.34	9	28	57	1.6	9
Core 2	UQCRC2	439	46.6	31	61	0.69	11	23	59	0.39	11	34	91	1.2	11
Cyt *b*	MT-CYB	379	42.8	2	9	0.02	16	1	2	0.004	16	2	5	0.07	16
Cyt *c1*	CYC1	241	27.3	12	39	0.18	18	7	32	0.12	16	12	43	0.84	17
Rieske	UQCRFS1	196	21.6	14	30	0.25	19	10	29	0.09	19	15	35	0.42	20
14 kDa	UQCRB	110	13.4	11	57	0.22	27	9	57	0.13	27	11	57	0.53	27
QP-C	UQCRQ	81	9.6	7	84	0.05	28	1	16	0.03	Am[c]	9	78	0.25	28
11 kDa	UQCRH	78	9.2	4	38	0.008	28	-	-	-	-	4	48	0.02	28
8 kDa	Subunit 11	78	7.9				31				31				31
7.2 kDa	UQCR10	63	7.3	3	50	0.04	31	2	38	0.04	31	4	52	0.06	31
6.4 kDa	UQCR11	56	6.5	1	21	0.001	31	-	-	-	-	1	21	0.002	31

[a]Subunits that are not expected in the sample are shown with data in grey, but in most cases are still detectable at lower levels.
[b]A dash indicates that no peptides were detected for the subunit.
[c]Ambiguous

Table S2. Lipid content of isolated SC I+III$_2$ particles compared to mitochondrial membranes, Related to STAR methods

SC I+III$_2$ Preparation	[Protein] mg/mL (n = 3-6)	[SC I+III$_2$] μM	[PO$_4$]$_{org}$ μM (n = 3-6)	[Lipid] μM[a]	nmole Lipid / mg Protein	Lipid molecules / SC I+III$_2$
1	9.1 ± 0.3[b]	6.3 ± 0.2	874 ± 85	728 ± 71	80 ± 8	116 ± 12
2	8.1 ± 0.4	5.6 ± 0.3	897 ± 55	748 ± 45	92 ± 7	133 ± 11
3	12.5 ± 0.3	8.6 ± 0.2	1298 ± 49	1082 ± 41	87 ± 4	126 ± 6
4	16.0 ± 0.2	11.0 ± 0.1	1537 ± 25	1281 ± 21	80 ± 2	116 ± 2
5	10.5 ± 0.6	7.2 ± 0.4	1023 ± 80	853 ± 67	81 ± 8	118 ± 12
				**Average:**	**84 ± 6**	**122 ± 8**
Suspended Mitochondrial Membranes	14.1 ± 0.6	---	2597 ± 198	2164 ± 165	153 ± 13	---

[a]Assuming ratio of 4:1 phospholipid:cardiolipin
[b]Mean ± SEM, throughout

Table S3. K_{cat} for CI and $CIII_2$ within the SC and Rate Coupling, Related to Figures 2 and 3

Condition	K_{cat} (s^{-1})<br>NADH oxidized per CI	 Cyt *c* reduced per $CIII_2$	 $CoQH_2$ oxidized per $CIII_2$	 Rate coupling[a] (cyt *c* reduced / NADH oxidized)
No DQ	9.9 ± 0.2	17.9 ± 0.2	8.9 ± 0.1	1.80 ± 0.05
10 μM DQ	53.4 ± 1.5	102 ± 2	50.8 ± 0.7	1.90 ± 0.06
100 μM DQ	45.2 ± 1.2	80.3 ± 1.7	40.1 ± 0.8	1.78 ± 0.06
100 μM DQ, 10 μM BSA	59.0 ± 1.5	70.6 ± 3.4	35.3 ± 1.7	1.20 ± 0.06
100 μM DQ, Lipid-Detergent (LD) buffer	188 ± 3	226 ± 2	113 ± 1	1.21 ± 0.02
Ratio of activity LD-buffer/10 μM DQ	3.52 ± 0.11	2.23 ± 0.04	1.11 ± 0.02[b]	

[a]The expected ratio for perfect rate coupling is 2.0 cyt *c* reduced / NADH oxidized

[b]This ratio was calculated between LD-buffer per Q_P site and 10 μM DQ per $CIII_2$ indicating that the increase in $CIII_2$ activity in LD-buffer may result from increased access to the $CIII_2$ active sites already present

Table S4. Cryo-EM data collection, Related to Figure 4

Data Collection and processing	
Microscope	FEI Titan Krios (at eBIC, Diamond UK)
Camera	FEI Falcon II
Magnification	100,000x
Voltage (kV)	300
Electron exposure ($e^-/Å^2$)	51
Defocus range (μm)	~-1.5 to -3.0
Pixel size (Å)	1.4
Software	RELION 2.1

Table S5. SC I+III$_2$ closed model overview, Related to STAR Methods						
Subunit Name Human / Bovine	Chain ID	#aa built / #aa total	Un-modelled residues	% atomic model	TMHs	Co-factors / Notes
**CI core subunits**						
ND1	D1	318 / 318	-	100	8	-
ND2	D2	347 / 347	-	100	11	antiporter-like
ND3	D3	115 / 115	-	100	3	TMH1-TMH2 loop becomes ordered in open/closed transition
ND4	D4	459 / 459	-	100	14	antiporter-like
ND4L	4L	98 / 98	-	100	3	-
ND5	D5	606 / 606	-	100	16	antiporter-like
ND6	D6	175 / 175	-	100	5	TMH3 undergoes π-to-α helix conversion in open-closed transition
NDUFS1 / 75 kDa	S1	688 / 704	1-5, 694-704	97.7	-	N1b (2Fe[75])<br>N4 (4Fe[74]C)<br>N5 (4Fe[75]H)
NDUFS2 / 49 kDa	S2	430 / 430	-	100	-	N1a (2Fe[24])<br>β1- β2 loop undergoes conformational change in open-closed transition
NDUFS3 / 30 kDa	S3	208 / 228	1-6, 215-228	91.2	-	-
NDUFS7 / PSST	S7	156 / 179	1-23	87.1	-	-
NDUFS8 / TYKY	S8	176 / 176	-	100	-	N6a (4Fe[TY]1)
NDUFV1 / 51 kDa	V1	429 / 444	1-9, 439-444	96.6	-	N6b (4Fe[TY]2)
NDUFV2 / 24 kDa	V2	212 / 217	1-4, 217	97.7	-	FMN<br>N3 (4Fe[51])
**CI Supernumerary Subunits**						
NDUFA1 / MWFE	A1	70 / 70	-	100	1	-
NDUFA2 / B8	A2	82 / 98	1-12, 95-98	83.7	-	Thioredoxin fold
NDUFA3 / B9	A3	74 / 83	1-9	89.2	1	-
NDUFA5 / B13	A5	111 / 115	1-4	96.5	-	-
NDUFA6 / B14	A6	114 / 127	1-13	89.8	-	LYR protein
NDUFA7 / B14.5a	A7	96 / 112	73-88	85.7	-	-
NDUFA8 / PGIV	A8	171 / 171	-	100	-	-
NDUFA9 / 39 kDa	A9	298 / 344	187-194, 253-277, 332-344	86.6	-	NADPH
NDUFA10 / 42 kDa	AJ	319 / 320	320	99.7	-	Nucleoside kinase family
NDUFA11 / B14.7	AK	140 / 140	-	100	4	-
NDUFA12 / B17.2	AL	123 / 145	1-22	84.8	-	-
NDUFA13 / B16.6	AM	139 / 143	1-4	97.2	1	Identical to GRIM-19
NDUFAB1 / SDAP	AA	80 / 88	1-4, 85-88	90.9	-	acyl carrier protein, phosphopantetheine acyl-chain
NDUFAB1 / SDAP	AB	87 / 88	1	98.9	-	acyl carrier protein, phosphopantetheine acyl-chain
NDUFB1 / MNLL	B1	52 / 57	1-5	91.2	1	-
NDUFB2 / AGGG	B2	64 / 72	1-4, 69-72	88.9	1	-

NDUFB3 / B12	B3	73 / 97	1-10, 84-97	75.3	1	-
NDUFB4 / B15	B4	128 / 128	-	100	1	-
NDUFB5 / SGDH	B5	139 / 143	1-4	97.2	1	-
NDUFB6 / B17	B6	95 / 127	37-66, 126-127	74.8	1	-
NDUFB7 / B18	B7	119 / 136	120-136	87.5	-	Double Cx_9C CHC domain
NDUFB8 / ASHI	B8	157 / 158	1	99.4	1	-
NDUFB9 / B22	B9	176 / 178	1, 178	98.9	-	LYR protein
NDUFB10 / PDSW	BJ	171 / 175	1-2, 174-175	97.7	-	-
NDUFB11 / ESSS	BK	102 / 125	1-21, 124-125	81.6	1	-
NDUFC1 / KFYI	C1	46 / 49	47-49	93.9	1	-
NDUFC2 / B14.5b	C2	119 / 120	1	99.2	2	-
NDUFS4 / 18 kDa (AQDQ)	S4	126 / 133	1-7	94.7	-	-
NDUFS5 / 15 kDa (PFFD)	S5	99 / 105	100-105	94.4	-	Quadruple CX_9C 2 CHCH domains
NDUFS6 / 13 kDa	S6	89 / 96	1-7	92.7	-	Zn^{2+}
NDUFV3 / 10 kDa	V3	41 / 75	1-34	54.7	-	-
**CI Total**		8117 / 8514		95.3		
**CIII Protomer 1**						
UQCRC1 / Core 1	a1	439 / 446	1, 223-228	98.4	-	Peptidase M16 fold
UQCRC2 / Core 2	a2	414 / 439	1-19, 228-333	94.3	-	Peptidase M16 fold
MT-CYB / Cyt *b*	b1	378 / 379	1	99.7	8	Two *b*-type hemes
CYC1 / Cyt *c1*	c1	240 / 241	241	99.6	1	covalently bound *c*-type heme
UQCRFS1 / Rieske	f1	196 / 196	-	100	1	2Fe2S cluster
UQCRB / 14 kDa	d1	100 / 110	1-10	90.9	-	-
UQCRQ / QP-C	q1	73 / 81	74-81	90.1	1	-
UQCRQH / 11 kDa	h1	65 / 78	1-12, 78	83.3	-	-
UQCRFS1N / 8 kDa	x1	78		0.0	-	poly-Ala
UQCR10 / 7.2 kDa	i1	55 / 63	1-4, 60-63	87.3	1	-
**CIII Protomer 2**						
UQCRC1 / Core 1	a3	444 / 446	1, 446	99.6	-	Peptidase M16 fold
UQCRC2 / Core 2	a4	413 / 439	1-19, 228-334	94.1	-	Peptidase M16 fold
MT-CYB / Cyt *b*	b2	378 / 379	1	99.7	8	Two *b*-type hemes
CYC1 / Cyt *c1*	c2	239 / 241	1, 240	99.2	1	covalently bound *c*-type heme
UQCRFS1 / Rieske	f2	195 / 196	1	99.5	1	2Fe2S cluster
UQCRB / 14 kDa	d2	101 / 110	1-8, 110	91.8	-	-
UQCRQ / QP-C	q2	75 / 81	1, 77-81	92.6	1	-
UQCRQH / 11 kDa	h2	65 / 78	1-12, 78	83.3	-	-
UQCRFS1N / 8 kDa	x2	78		0.0	-	poly-Ala
UQCR10 / 7.2 kDa	i2	57 / 63	1-2, 59-63	90.5	1	-
**$CIII_2$ Total**		3927 / 4222		93.0		
